# Emotional Dysregulation in Children and Adolescents With Psychiatric Disorders. A Narrative Review

**DOI:** 10.3389/fpsyt.2021.628252

**Published:** 2021-10-25

**Authors:** Frank W. Paulus, Susanne Ohmann, Eva Möhler, Paul Plener, Christian Popow

**Affiliations:** ^1^Department of Child and Adolescent Psychiatry, Psychosomatics and Psychotherapy, Saarland University Medical Center, Homburg, Germany; ^2^Department of Child and Adolescent Psychiatry, Medical University of Vienna, Vienna, Austria; ^3^Austrian Society of Cognitive Behavioral Therapy (OeGVT), Vienna, Austria; ^4^Department of Child and Adolescent Psychiatry and Psychotherapy, Regional Psychiatric Hospital, Mauer, Austria

**Keywords:** emotional dysregulation, psychiatric disorders, treatment, psychopathology, children, adolescents, mental disorder

## Abstract

**Background:** Emotional dysregulation (ED) is a transdiagnostic construct defined as the inability to regulate the intensity and quality of emotions (such as, fear, anger, sadness), in order to generate an appropriate emotional response, to handle excitability, mood instability, and emotional overreactivity, and to come down to an emotional baseline. Because ED has not been defined as a clinical entity, and because ED plays a major role in child and adolescent psychopathology, we decided to summarize current knowledge on this topic based on a narrative review of the current literature.

**Methods:** This narrative review is based on a literature search of peer-reviewed journals. We searched the databases ERIC, PsycARTICLES, PsycINFO and PSYNDEX on June 2, 2020 for peer reviewed articles published between 2000 and 2020 in English language for the preschool, school, and adolescent age (2–17 years) using the following search terms: “emotional dysregulation” OR “affect dysregulation,” retrieving 943 articles.

**Results:** The results of the literature search are presented in the following sections: the relationship between ED and psychiatric disorders (ADHD, Mood Disorders, Psychological Trauma, Posttraumatic Stress Disorder, Non-suicidal Self-Injury, Eating Disorders, Oppositional Defiant Disorder, Conduct Disorder, Disruptive Disruptive Mood Dysregulation Disorder, Personality Disorders, Substance Use Disorder, Developmental Disorders, Autism Spectrum Disorder, Psychosis and Schizophrenia, and Gaming Disorder), prevention, and treatment of ED.

**Conclusion:** Basic conditions of ED are genetic disposition, the experience of trauma, especially sexual or physical abuse, emotional neglect in childhood or adolescence, and personal stress. ED is a complex construct and a comprehensive concept, aggravating a number of various mental disorders. Differential treatment is mandatory for individual and social functioning.

## Introduction

Emotions are strong and visible feelings that allow us to adapt to our environment. These feelings emerge in reaction to pleasant or unpleasant internal or external stimuli, helping us to react even before we rationally may analyze and deal with the stimulus.

Emotion regulation (ER) is the ability to recognize, evaluate, modify, and manage emotions in a personal and socially acceptable way, in order to maintain mental control over strong feelings, and arrive at adaptive functioning (1–4). This is achieved by applying various goal oriented, adaptive strategies, e.g., acceptance, problem solving, and reappraisal (5). One frequently cited definition (1, pp. 27–8) states that ER “consists of the extrinsic and intrinsic processes responsible for monitoring, evaluating and modifying emotional reactions, especially their intensive and temporal features, to accomplish one's goals.” Another attempt to define ER (2) emphasizes awareness, understanding, accepting emotions, and the ability to implement strategies that modulate emotional responses in a flexible and appropriate way, while considering situational demands.

Social cognition comprises the mental operations that enable social interactions. Social cognition and interactions need well-functioning ER abilities and (later on) intact theory of mind (TOM) capacities. Newborn infants and toddlers learn ER through the interaction with their sensitive and reliable attachment persons. ER helps in initiating, inhibiting and modulating actions that are triggered by emotions. TOM, the ability to reason about one's own and others' mental states, develops later, and is necessary to understand and predict the actions of other persons. It is learned by experiencing and analyzing synchronuous and asynchronuous social interactions.

Neurobiologically, the basic emotions are represented subcortically with projections throughout the brain, reaching the cingulate cortex, hippocampus, amygdala, and the insular cortex (6), and are modulated by forebrain structures. Glutamatergic/GABA-ergic balance plays an important role in emotional control (7). The “limbic cerebellum” is also involved in ER, and congential malformations or later acquired lesions may lead to ED (8). ER neural circuits include rostral and subgenual regions of the anterior cingulate, the orbitofrontal and the dorsomedial prefrontal cortex (PFC), and regions involved in executive and attentional control, the dorsal anterior cingulate, ventrolateral PFC, and dorsolateral PFC (9, 10). Besides genetically determined malformations, genetic polymorphisms [e.g., (11)], and functional connectivity problems may cause structural damage. Acute and chronic stress may have long-term consequences. Especially longer lasting stressful life events alter the CNS structures and functionality, leading not only to persisting neurological, social and behavioral dysfunctions but also contribute to the development of pre-disorders later in life.

The ability to regulate emotions develops in early childhood within a process starting at birth (12). Babies learn from interacting with their caregivers 1. To differentiate their primary emotional states (neutral, pleasurable, and not pleasurable), and 2. That these states are variable in intensity, and can be modified, later on by using self-control, self-soothing or distracting. Learning to recognize and understanding emotions in the interaction with a sensitive caregiver is a prerequisite for later self-regulation (13–15). At the age of 3 years, children already understand their emotions (16). Various processes, such as the development of executive functions and language influence the development of ER (17–19). Next to the encoding of internal emotional cues, ER involves accessing of coping resources, using a broad range of regulation strategies (20). The development of these strategies is complex, involving genetics, epigenetics, cognition, social experiences, and learning (20, 21). Children acquire their primary regulation strategies that include help seeking, avoiding, redirecting attention, suppressing impulses, and problem solving, by the age of seven (20). Later on, ER becomes more and more self-controlled.

Internal and external factors determine the efficacy of ER: internal factors comprise neuroregulatory reactivity, temperament, cognitive abilities attachment and related positive internal working models; external factors are related to caregiving style, behavioral models, and experience (22).

Children express negative emotions in order to regulate their own emotions and to appropriately communicate with others. Under psychopathologic conditions, one or more negative emotions (such as sadness, panic, anger) are experienced either overly intense or exceedingly long, and fail to be adaptive (23). As such, healthy social–emotional functioning is contingent on being able to dynamically respond to contextual demands in a culturally appropriate way (19).

Emotion Dysregulation (ED), a trans-nosologic condition, manifests as maladaptive processing of external or internal stimuli when ER strategies and processes are impaired (24). Clinically, hyperarousal, mood instability, irritability, aggression, and temper tantrums are observed (25). Reactions appear excessive to social norms, and inappropriate or detrimental to a person's interests (26). They are often influenced by internalizing or externalizing problems or comorbid disorders, such as anxiety, autoaggression, borderline personality disorder (BPD), post traumatic stress disorder (PTSD), uni- or bipolar affective disorders (21, 25, 27–29). ED reflects a limited set of problematic strategies to understand or accept one's own emotional states, and disposing of a relatively limited set of strategies for dealing with one's own emotional states (2). ED has received considerable attention in the last decades because of its negative effects on emotional development, cognitive and behavioral adaptation, self-efficacy, social relationships and functioning, and quality of life (27, 30).

Attempting to provide an overview on the various aspects of ED in children and adolescents with psychiatric disorders, focusing on clinical characteristics, prevention, and therapy, we explored the scientific literature for relevant contributions in the last 20 years.

## Methods

**Figure 1:**
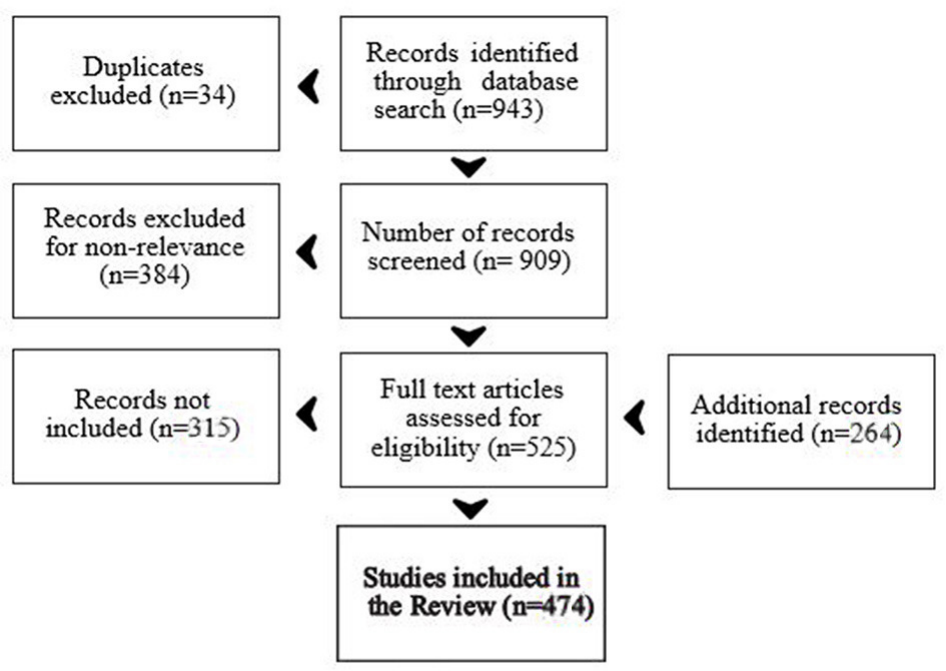
Processing of records.

This narrative review is based on a thorough literature research in peer-reviewed journals. We searched the literature databases ERIC, PsycARTICLES, PsycINFO and PSYNDEX on June 29^*th*^, 2020 for peer reviewed articles on ED in children and adolescents, published in English language between January, 2000 and June, 2020 related to children and adolescents (2–17 years). Using the search terms, “emotional dysregulation” OR “affect dysregulation,” we retrieved 943 articles (cf Figure 1). After removing duplicates, 909 articles remained and were screened by title and abstract for the appropriateness of the contents. We thus excluded 384 articles: 1. dealing with subjects outside the target age range (≥ 2 years ≤ 17), 2. related to ED or affect dysregulation of the parents and the resulting parent-child interaction, 3. anecdotical or single case reports, 4. articles without ED or affect dysregulation being the main focus, such as articles on Disruptive Mood Disorder Dysregulation (DMDD), emotions and regulation of emotions without reference to ED, dysregulation of other functions, and articles on ED as future research objective without current data, 6. articles mainly dealing with neurobiology of the emotions, neuro-pathophysiology, and stress. Thus, excluding 384 articles, we thoroughly screened 525 articles for their relevance in terms of content and subjective importance, and retained 210 articles of primary research (excluding another 315 articles), and retrieved 264 additional articles seen as “necessary,” and retrieved during the course of the writing process. The additional, secondary references were retrieved in addition, related to 1. references on subtopics, 2. references cited in the initially retrieved references and estimated as important, 3. our own knowledge of the literature, 4. recommended by the two reviewers and the editor.

This sums up to a total of 474 articles cited in this narrative review (cf. Figure 1 for a summary of article handling).

## Results

In the following, we describe various neuropsychiatric disorders that are related to or influenced by ED. We **first** describe the clinical picture, then the neuropsychiatric background and various facettes of the disorder, and the impact of ED for the psychopathology of the disorder.

### Psychopathology and ED

#### ADHD

ADHD is a common, mostly inherited neuropsychiatric disorder with various degrees of severity (31, 32) and three predominant presentations that may change ove time: inattentive, hyperactive – impulsive, and combined. Since 1995 (33), ED has been recognized as a key problem in children and later in adults with ADHD. Social, behavioral, and educational problems may already be present in early childhood (34), and continue into adulthood with severe clinical, personal, and vocational sequelae (35–37). A few studies have shown that ED occurs independent of the ADHD subtype (38, 39), whereas others (40, 41) found an increased incidence of ED in children with the combined type.

An underdeveloped working memory (18) and problems of impulse inhibition may contribute to ED. Thereby emotional impulsiveness and problems of impulse inhibition are associated with greater emotional and behavioral dysregulation (42, 43). Children with ADHD express more negative affect, a higher emotional instability, and difficulties in regulating and expressing their emotions (44–46). They also have difficulties in recognizing and understanding the emotions of others (44, 46). In addition, young children with ADHD have an attention bias toward positive emotions (47).

Children with ADHD perform worse in a go/no-go task when meaningful stimuli are provided in parallel (48). They also exhibit more parasympathetic dysregulation and less sympathetic reactivity, although López-Martín et al. (49) found no differences in autonomous activation during go/no-go task performance comparing children with and without ADHD. Children with ADHD need stronger activation of inhibition-related neural mechanisms in order to achieve a similar performance, especially in emotional contexts. This explains why children with ADHD have difficulties in controlling their behavior and emotions in an emotionally burdened situation. Furthermore, in boys, Seymour et al. found an association between ED unique subregion expansion in the right globus pallidus, putamen and amygdala (50).

ER mediates the association between ADHD and social skills in youth (51). Less distinct emotional and social competence could explain the higher degree of peer rejection in children with ADHD (52). In addition, the common sleep problems in children with ADHD will further aggravate their attentional and emotional dysregulation (53). Medication with methylphenidate reduces ED by reducing impulsivity in children with ADHD (39, 54, 55).

#### Mood Disorders

The spectrum of affective mood disorders comprises unipolar, bipolar, schizo-affective, dysthymic, cyclothymic, and adjustment disorder with depressive reaction and has been conceptualized a disorder of ER. Mood disorders run in families but may also be triggered by negative experiences. The severity of mood disorders may vary, and typical manifestations are named major disorders (31, 32). Dysregulation of positive and/or negative affect in affective disorders include under- or overreactivity to stimuli, abnormalities in the time flow of an emotional response, for example, in maintaining or enhancing positive affect or in limiting sadness (56). FMRI studies showed an exaggerated amygdala response to negative, and attenuated amygdala responses in reaction to positive stimuli (57).

##### Depression

Depressive symptoms are linked to a disrupted regulation of negative emotions (58, 59). Some studies indicate that ER difficulties precede the onset of depressive symptoms (58, 60). Depressive symptoms have also been associated with overregulated negative affect (61). Children with depressive symptoms do not experience more difficulties than their peers in regulating positive affect but return more slowly to their emotional baseline following a depressive reaction to negative feedback (62).

Children and adolescents with depressive symptoms are more likely to engage in rumination than using active ER strategies, such as problem solving, distracting or cognitive reappraisal (63). Symptoms of depression might overstrain a child's ER capacities. Peer rejection and failing to down-regulate negative and up-regulate positive emotions may lead to diminished self-confidence and failing to perceive and control one's own feelings, thus aggravating depressive symptoms (21, 64).

##### Bipolar Disorder

Children diagnosed with bipolar disorder (BD) fail to remit depressive symptoms and affective instability (65). Structural abnormalities in the orbito-frontal and subgenual white matter are consistent with neuro-biological models that implicate dysregulated affective systems and impulsiveness in BD (66). Mood dysregulation centered around limbic overactivity and relative prefrontal underactivity, indicate decreased prefrontal influence on limbic structures mediating mood regulation (67). Functional dysconnectivity of the Inferior Frontal gyrus is involved in ER and accounts for trait abnormalities in children with BD (68). Severe dysregulation of affect and behavior is associated with difficulties in falling asleep and sleeping through (69, 70). In addition, ED is associated with a greater impairment of episodic memory (71).

Kim et al. (72) found abnormal gaze patterns as a a potential endophenotype for difficulties of labeling emotions in patients with BD. Difficulties ascertaining the correct emotional tone of a spoken sentence may possibly contribute to ED in youth with BD (73). Non-verbal emotion labeling deficits such as misinterpreting facial expressions may reflect general abnormalities in emotion processing and contribute to poor emotion regulation skills (15, 74). Children with BD require higher levels of emotional intensity to accurately interpret emotional expressions and have difficulties in differentiating subtle variations in the intensity of facial expressions (75).

##### Cyclothymia

Cyclothymia manifests as early-onset, enduring reactive mood fluctuations. ED is one of its core features, manifesting with extreme mood instability and reactivity (76). In children and adolescents, cyclothymic temperament is one of the strongest predictors of BD (77) that, as mentioned above, is also strongly connected to ED. Akiskal et al. (78) described the prototypical emotional symptoms of cyclothymia in adolescents as intermittently intense emotionality (sullen, irritable, restless, boastful) that shifts from one phase to another.

Compared to other manifestations of affective disorders, such as depressed, hyperthymic, or irritable mood, cyclothymia is associated with the most severe emotional and behavioral problems, such as sleep anxiety, separation sensitivity, eating disorders in girls, and antisocial-aggressive behavior in boys. The association of cyclothymia, sleep anxiety, and antisocial-aggressive behavior increases with age and is related to both, internalizing and externalizing disorders (79).

##### Suicidality

Suicidality is strongly associated with psychiatric disorders and ED. Linehan's (80) biosocial theory suggests that ED is a key factor in maintaining suicidality: adolescents experiencing that their exaggerated emotional states are not well-accepted by their environment will experience feelings of guilt and shame. Suicidal ideation is then a strategy to reduce or avoid these overwhelming negative emotions (81). Interestingly, poor positive affect is a stronger predictor of suicidality than increased negative affect (82). Depression is the most common psychiatric condition leading to suicide (83). Difficulties in regulating emotions may contribute to increased reactivity toward interpersonal stressors in depressed and suicidal adolescents (84).

#### Psychological Trauma

Early, recurrent, severe, chronic interpersonal, developmental, cumulative traumatic experiences (defined by number, duration and severity of trauma), and poly-victimization, are associated with ED [e.g., (85, 86)], often presenting with severe dysregulation of physical, affective, behavioral, cognitive, and interpersonal functioning [e.g., (87)]. These symptoms are merely related to dysfunctional coping strategies [e.g., (85, 88)].

Interpersonal trauma experience (sexual, physical and emotional abuse) and post-traumatic stress is associated with various psychiatric comorbidities and psychosocial, developmental, and physical impairment [e.g., (87, 89)], and a reduced ability to understand and regulate emotions [e.g., (87, 90)]. Difficulties in regulating emotions is a consequence of trauma, as well as a predictor of psychopathology (91).

In traumatized children and adolescents, ED

- increases the risk of psychopathology (89)
- is a feature of developmental trauma disorder (DTD) (85, 92, 93)
- is related to increased negative affect (90), and negative mental health outcomes (94)
- mediates the relationship between childhood trauma and the resulting internalizing/externalizing behavior problems [e.g., (86, 95–97)], between severe trauma experience and the resulting symptomatology (90, 98), and between maltreatment of children and their aggression toward peers (99)
- is a core feature accounting for the increased risk of acute and life-time impairment [e.g., (87)], and an important target for therapeutic interventions (87)
- mediates or moderates treatment related changes (100)

The DSM-5 classification relates three disorders to childhood trauma and maltreatment: PTSD, Reactive Attachment Disorder, characterized by low social-emotional responsiveness and ED, and Disinhibited Social Engagement Disorder, characterized by active approaches to and interactions with unfamiliar adults (101). Abusing and neglectful parents mostly suffer themselves from severe harmful personal experiences (102).

##### Child Misuse, Maltreatment, Neglect

Child misuse, maltreatment and neglect, later reported by about a third of adults (103), relate to significant harm, lead to impaired health or development, is responsible for about 45% of mental disorders in childhood (104), with effects persisting into adulthood (105). There is also a transgenerational risk of later abusive parenting (106). Warmingham et al. (107) described three often overlapping subgroups: 1. chronic multi-subtype maltreatment (57%), 2. only neglect in a single developmental period (31%), and 3. single subtype of maltreatment (emotional maltreatment, physical or sexual abuse) occurring in a single developmental period (12%).

According to the self-trauma theory (108, 109), a child's coping strategies with stressful experiences depend on intact ER skills. If a trauma occurs during the development of ER and interpersonal skills, it may interfere with the normal development, leading to an increased risk of becoming emotionally overwhelmed “by trauma reminders and future stressful events”. According to the betrayal trauma theory (110), post-traumatic symptoms, including ED, may develop if a child is repeatedly maltreated, physically or sexually abused or neglected by an originally trusted close person. Then, the post-traumatic symptoms (e.g., dissociation) develop in order to protect the child's attachment to his/her significant person because during this developmental phase, significant persons are not allowed to fail.

##### Betrayal Trauma and ED

Trauma negatively influences the ability to regulate anger and affect (111). Longitudinal studies demonstrated that higher levels of ED are associated with aggressive behavior over time [e.g., (112, 113)]. Symptoms of ED relate more to reactive (i.e., “hot,” impulsive aggression in response to perceived provocation or threats), than to proactive aggression (i.e., “cold,” instrumental, goal-oriented aggression) (114).

Adolescents exposed to betrayal trauma had severe difficulties with ER, reported more severe PTSD symptoms, and expressed a more aggressive communication style (115). ER difficulties mediated the relationship between betrayal trauma and negative interpersonal communication skills.

ED [undercontrolled/ ambivalent or overcontrolled/ unresponsive regulation (116)] resulting from adverse childhood experiences (ACE), and especially neglect (117), may manifest in response to stress (118) as biased perceptions of threat (119), increased responsivity to negative information with increased reactivity of the autonomous nervous system, based upon gene-environment interactions (117), and leading to increased irritability (120), poor problem solving skills (121), more negative emotionality (122), poor academic performance (123), and internalizing or externalizing reactions (89, 124, 125).

##### Internalizing and Externalizing Reactions

Internalizing reactions and victimization are associated with depression, anxiety disorders, addictive behaviors, painful medical conditions (126), self-harm (127), and PTSD (89). Externalizing reactions are associated with pervasive anger, aggression, impulsiveness, risky sexual behavior, intimate partner violence, and conduct problems, accentuating victim-perpetrator dynamics, and precipitating culprid-victim thinking and juvenile delinquency (122, 124, 128). Internalizing and externalizing problems may also appear as comorbid conditions (129), are mediated by ED (97), and may trigger suicidality (130) and early death (131). Witnessing violence (132) and harsh discipline (133) may lead to externalizing and aggressive behavior. Irritability may also be related to antisocial, borderline and narcissistic personality disorders, most frequently represented among delinquent and incarcerated youth (134) (cf. chapter Personality Disorders). Resilience in maltreated children is, however, rare (123).

##### Maternal Influences

Trent et al. (135), studying inpatient psychiatric patients with depressive symptoms and childhood exposure to maternal threatening behaviors, observed that maternal threatening behavior was related to the severity of depressive symptoms in those children who had more pronounced deficits in emotional clarity [which is defined by Gratz & Roemer as “the extent to which individuals are confused about the specific emotions they are experiencing” (2)]. Pat-Horenczyk et al. (28) confirmed maternal ER mediating the association between maternal PTSD and children's ED in a community sample of traumatized Israeli mothers and children.

##### Substance Misuse in Traumatized Patients

Substance misusing and socially disadvantaged mothers showed less observed emotional availability for their 12–42 months old children than mothers without social disadvantage and substance abuse (136). The former were also more often traumatized in childhood and exhibited more often borderline personality traits, such as higher levels of emotional distress and poor ER. Mothers having experienced childhood adversities had difficulties in providing appropriate caregiving when exposed to highly stressful conditions (136). At the same time, childhood trauma increases the risk of later substance abuse because of a limited access to ER strategies [e.g., (137)]. Furthermore, traumatic experiences during childhood are indirectly associated with suicidal ideation through non-acceptance of emotional responses, limited access to functional ER strategies, and lacking emotional awareness (138).

##### Sexual Abuse and Sex Trafficking

Especially sexual abuse is associated with post traumatic symptoms, such as dissociation, significant impairment of psycho-social adjustment and self-regulatory abilities, and ED. Because disclosure of maltreatment would lead to serious consequences within the family, the child remains helpless, cannot stop maltreatment, even not express his/her emotions, and has to adapt to the dysfunctional environment (139). The consequences are impaired emotion recognition and management, the development of maladaptive coping strategies, such as dissociation, self-injurious behavior (140, 141), and internalizing or externalizing behavior, interrupting the emotional development and the developing of constructive peer relationships, causing peer rejection, with sequelae persisting into adulthood (95, 139, 141–143). 30% of sexually abused children are <7 years old (144), putting this most vulnerable group at extreme risk for later (transgenerational) malfunctioning, and severe health-related problems. Because especially small children need sensitive and functional caregivers, they may dissociate their experiences of abuse and blame themselves. This will lead to ED, sleep difficulties, and poor attachment behavior. Later on, females are at increased risk of developing sexual anxiety and re-victimization in romantic relationships (145). Sexual assaults on adolescent girls will lead to PTSD, complex PTSD, and life-impairing disturbances in self-organization, ED, negative self-concept, and interpersonal problems, leading in about 40% to continued traumatization (146).

Research on the impact of developmental trauma on juvenile victims of sex trafficking is limited. Greenbaum (147) summarized the (limited) knowledge about child sex trafficking. Using qualitative research methods, Hopper et al. (148), analyzed hospital charts of sex trafficking youth and found that already existing ED and behavioral problems increased the vulnerability for sex trafficking. Thus, a vicious cycle of ED in traumatized children may be detected: on one hand, ED can be seen as a consequence of trauma (91), on the other hand, children and adolescents with ED are at risk to be exposed to traumatizing situations.

##### Trauma and ED

The neurological regions of interest for the regulation of emotions are the prefrontal cortex and the amygdala (149). If the maturation of the associated pathways is delayed until early adulthood, this asynchronous development may lead to problems of ER and decision-making. Neurobiologically, stressful situations in early life lead to early and persistent changes in the amygdala circuitry and function (150). According to Cohen and colleagues, these functional changes do not seem to recover even after the stressor is eliminated, and to persist despite developmental changes in the prefrontal regions for regulating emotions. In a study on 553 children aged 10–12 years, Fishbein et al. (151) found that exposure to personal stressors affected at least one neurocognitive function: community stressors were related to problems of recognizing emotions and problem-solving abilities, neglect was related to problems of recognizing emotions and deficits of intellectual abilities, and physical abuse was related to disturbed problem-solving abilities. Cicchetti et al. (152) found decreased afternoon cortisol levels in children experiencing early physical and sexual abuse in 168 school aged maltreated children, indicating persistent neuro-endocrine dysregulation of the HPA axis. Maltreated children present with neuro-endocrine dysregulation of the HPA axis only if they experienced physical or sexual abuse in the first 5 years of life, and if they suffered from depression or other internalizing problems (152).

Early experiences of maltreatment and neglect, leading to ED is extremely common (98%!) in adopted children (153, 154). There is also a high comorbidity of ADHD and conduct and attachment disorders, and in about 2/3 with continued, severe personal and social difficulties despite happy placements. 38% do not achieve a stable adoption. Studies of the hypothalamic-pituitary-adrenal (HPA) axis showed that 6 months after adoption, morning cortisol levels were improved but, also post adoption, dysregulation of the HPA axis was associated with more emotional and behavioral problems (129), possibly increasing the risk of negative developmental outcomes. Therefore, early interventions, even involving out of home care are justified to enable a sustainable development of vulnerable children if no change of the detrimental environment may be expected (155). The effect of such serious measures will not be paramount but will at least represent an opportunity (153), especially if appropriate care is established before the age of 6 months (156).

#### Posttraumatic Stress Disorder

PTSD is a disorder of ED (94, 157), and represents an individual's attempt to achieve an emotional equilibrium following severe traumatic experience(s) (157). Intrusions emerge from emotional under-regulation, whereas emotional numbing, avoidance, and dissociation are indicative of emotional over-regulation (158). ED is a critical risk factor for developing [e.g., (89, 94, 159–162)] and maintaining PTSD [e.g., (89, 160–162)].

Examining neural underpinnings of ED in pediatric PTSD, Wolf & Herringa (163) found that adolescents with severe PTSD showed abnormal function and connectivity in prefrontal–amygdala circuits. These changes are related to threat processing and fear regulation. Adolescents with PTSD demonstrated an age-related decline of dorsomedial PFC activation, inversely related to the severity of PTSD, and an age-related decrease of the PFC - amygdala connectivity. The authors suggested abnormal developmental processes to influence key emotional pathways of pediatric PTSD.

##### Age Dependent Characteristics

Infants and toddlers are especially vulnerable to traumatic experiences, and therefore at high risk of developing severe PTSD, ED, internalizing and/or externalizing symptoms, and long-term impairment (89, 164–166). PTSD in toddlers differs from PTSD in older children and adults in relation to the severity and number of symptoms, e.g., toddlers express less avoidance or numbing [e.g., (167, 168)].

Difficulties of ER, escpecially emotional clarity (2), play an essential role in trauma-related psychopathology. Viana et al. (169) showed that lower emotional clarity, a sub-dimension of ED, indirectly influences the severity of PTSD because of an increased sensitivity for anxiety. Viana et al. (170) also found lower emotional clarity in traumatized adolescents to be related to suicidal ideation at higher (and not lower) levels of distress tolerance.

Younger age at traumatization (below the age of 14 years) relates to increased psychopathology, including ED and PTSD (159, 171). ED, negative self-concept, interpersonal problems and core PTSD symptoms represent moderately correlated dimensions in traumatized adolescents (146). There is an ED related link between

- Violence exposure and PTSD (172),
- Depressive symptoms and PTSD (173),
- Traumatic exposure and reactive aggression (174).

Assessing the inter-generational impact of ED, Powers et al. (94) investigated 105 African American mother-child dyads and found PTSD significantly associated with childhood trauma experience, maternal depressive symptoms, ED, and maternal child abuse. The authors considered ED a trans-diagnostic treatment target across the life span, and recommended treating maternal ED in order to reduce traumatizing of the next generation. PTSD includes various heterogeneous symptom clusters - specific factors, such as type of trauma exposure or ED - influencing the severity of symptoms within these clusters and leading to distinct clinical phenotypes of PTSD (175). Empirically, there are various facets of ED, such as lack of emotional awareness, lack of clarity of emotions, difficulties of controlling behavior, achieving goal-directed behavior, non-acceptance of emotional responses, and limited access to problem-solving strategies. These facets act as indirect pathways through which trauma is associated with specific DSM−5 PTSD symptom clusters, such as intrusion, avoidance, negative alterations and arousal (175).

##### PTSD and Behavior

PTSD predicts aggressive and delinquent behavior in youth, especially in those who are also experiencing high levels of ED (174). Miller and Marsee (176) compared two groups of incarcerated boys, a low reactivity group with symptoms of emotional numbing and callous-unemotional (CU) traits, and a high reactivity group with symptoms of hyper-arousal and ED. Frequent violent offending, CU traits, and proactive aggression correlated with emotional numbing and combined hyper-arousal symptoms. Delinquent adolescents experienced high levels of ED (141, 175), girls more than boys (141, 177). ED also predicted a higher risk for subsequent offending behavior (177). Especially interpersonal trauma exposure corresponds to negative effects on youth's psychological functioning, severe PTSD symptoms [e.g., (146, 175, 178, 179)], poor ER strategies [e.g., (95)], and diffiulties in self-organization [e.g., (146)]. Delinquent adolescents who experienced sexual abuse may show higher rates of PTSD (141).

##### Poly-Victimization

Experiencing repeated traumatic events is an important predictor for developing PTSD [e.g., (94, 180)]: Lehmann et al. (101) found a strong association between the number of self–reported potentially traumatic events and the development of PTSD in a sample of adolescents raised in foster care. Youth living in foster care had an increased risk of developing PTSD (101, 181, 182). PTSD and ED were significant predictors of depressive symptom trajectories, more pronounced in females than in males (182). The risk of more severe ER difficulties, ED, and PTSD is higher in poly-victimized adolescents, having experienced multiple types of interpersonal and non-interpersonal childhood traumata (101, 175, 183–185), girls again being more severely affected than boys (101, 185).

Charak et al. (186) investigated a large sample of incarcerated adolescents, and studied associations between poly-victimization, ED, DSM-5 PTSD symptoms, and related behavioral health problems, including alcohol/drug misuse, anger, irritability, depression, anxiety, somatic complaints, and suicide ideation. The authors distinguished three distinct sub-groups:

1. violent environment (such as being exposed to natural disasters, accidents, war, physical abuse or assault, witnessing physical violence, unexpected death of a beloved person, facing a dead body (excluding at funerals), painful medical treatment, and acts of violence
2. poly-victimization (such as, serious injuries, psychological abuse, domestic violence, family members being badly injured or sick, parental drug use, unexpected death of someone close, removal from parental custody or parental threats of abandonment, neglect, and sexual abuse); psychopathology on all four DSM-5 PTSD symptoms clusters, as well as depression/anxiety, somatic complaints, and suicidality
3. mixed adversity (such as, a parent being incarcerated, or someone they knew had attempted suicide, or was severely injured or ill, experience of physical abuse). Youth in the “mixed adversity class” reported about exposure to traumatic events (such as severe accidents), and were less likely having been exposed to violent victimization.

There is a positive relationship between developing and maintaining PTSD, and avoiding trauma-related emotions, thoughts and activities (187). Woodward et al. (188) confirmed this theory for traumatized inpatient adolescents, showing positive associations of emotional non-acceptance and greater distraction- coping in relation to more severe PTSD.

##### Influence of Comorbid Disorders

Various psychiatric disorders increase the risk of being exposed to traumatic events (189). For Dvir et al. (87), bipolar disorder is the best example of psychopathology involving ED. Biederman et al. (189) indicated that children with Bipolar-I disorder are at a 20-fold risk to develop full or subthreshold PTSD compared to healthy children. In patients with early non-affective psychosis, Liu et al. (190) found maladaptive ER strategies (catastrophizing, ruminating, and blaming others), global ED, and poor cognitive insight as psychological risk factors for PTSD.

##### Developmental Trauma Disorder

The complexity of early childhood developmental trauma is not optimally covered by a PTSD diagnosis. Hence, Developmental Trauma Disorder (DTD) is a proposed diagnosis for children, who have experienced disrupted attachment and multiple or chronic exposure to developmentally disabling interpersonal traumata, such as emotional or sexual abuse, abandonment, threats to physical integrity. Seven levels of functioning are involved in DTD (88, 191):

1. Attachment (e.g., restricted attachment in the form of a distrustful behavior pattern toward attachment figures as well as toward protective social institutions).
2. Biology (e.g., stress hormones).
3. Cognition (e.g., depersonalization, derealization, confusion, sense of safety).
4. ER (impairments in the regulation of e.g., anger, fear, resignation, defeat).
5. Behavioral control (e.g., acting out, cutting, re-enacting).
6. Dissociation.
7. Self-concept (e.g., self-attribution, self-hate, self-blame). Most often, DTD involves complex traumatic experiences in childhood, usually corresponding to sexual, physical abuse or war experiences in early childhood (92).

#### Non-suicidal Self-Injury and Suicidality

According to the biosocial theory of Marsha Linehan (80), individuals use deliberate self-harm and self-injurious behavior as maladaptive ER strategies against overwhelming intense negative emotions [e.g., (127, 192)]. Because of their increased emotional reactivity and lability, and their immature prefrontal control, adolescents have a higher risk for engaging in extreme dysfunctional ER strategies, such as Non-Suicidal Self-Injury (NSSI) [e.g., (193)]. NSSI therefore typically begins in adolescence (192). NSSI:

1. is defined as direct and deliberate damage of body tissue without conscious suicidal intent [e.g., (192, 194–196)], and for not culturally sanctioned purposes (31).
2. is triggered by ED (80), and predominantly goes along with high levels of emotional distress (197).
3. assists in the escape, management, or regulation of emotion (198), in particular in the escape of negative emotional states, such as anger, depression, loneliness and frustration, and unwanted thoughts (199).
4. is maintained by positive and negative reinforcement in intra- and interpersonal domains (192).
5. has a serious impact on health and well-being [e.g., (200)], and
6. increases the risk of later suicide (201), especially if low emotional clarity (2) is linked to high distress tolerance (169).

##### NSSI and Borderline Personality Disorder

“The relationship between NSSI and BPD features in adolescence seems to be more controversial than in adulthood” (203, p. 24). Recurrent NSSI is a core feature of BPD (31) and often precedes suicidal behaviors in adolescents [e.g., (202)]. Specific aspects of ED, such as lack of emotional awareness, poor coping strategies, and non-acceptance of emotions, predict repeated NSSI [e.g., (2, 203)] and are highly prevalent among suicidal adolescents, regardless of their psychiatric diagnoses [e.g., (82)].

NSSI is included in the DSM-5 (31) as a condition requiring further study, and classified as an independent diagnostic entity since 2013. ED and NSSI are closely related [e.g., (193, 204–206)]. ED in NSSI is considered

- A risk factor [e.g., (141, 207)], and a core feature of NSSI [e.g., (208, 209)],
- The primary drive for NSSI in adolescents [e.g., (81)],
- A major factor for developing (200) and maintaining NSSI [e.g., (210)].

NSSI and BPD overlap in adolescents, 52% of adolescents practicing NSSI suffer from BPD (193). Underlying mechanisms for both disorders, although to a different degree, include affective instability (i.e., ED), and interpersonal instability (i.e., instability of attachment to significant others). Interpersonal instability in BPD is more generalized, extending beyond family functioning to peer relationships (205).

Sadeh et al. (211) investigated the relationship between BPD symptoms and NSSI: BPD affective dysregulation was associated with intra- (e.g., affect regulation, anti-dissociation and self-punishment) but not interpersonal functions (i.e., peer relations, autonomy) of NSSI. In contrast, BPD interpersonal dysfunction was associated with inter- rather than intrapersonal functions of NSSI. These data indicate that clusters of BPD symptoms show unique relationships with functions of NSSI in adolescents. Somma et al. (201) found a moderate association between self-reported features of BPD and NSSI in a sample of non-clinical adolescents, not fully explained by ED. The authors suggested that NSSI may represent just one of several dysfunctional ER strategies in adolescents at risk for BPD, and that NSSI in adolescence may not represent an exclusive ER strategy but may fulfill various other intra- and interpersonal needs.

Nakar et al. (212) found three distinct developmental trajectories for self-reported harmful behaviors in a community-based adolescent sample: self-injurious behavior, suicidal behavior, and substance misuse.

High-risk trajectories for the three behaviors greatly overlapped (80–90%), and this overlap was significantly associated with higher levels of BPD. The authors found a symptom shift, typically associated with BPD in adolescents: the high-risk trajectory of self-injurious behavior, and the high-risk trajectory of suicidal behavior had a high initial degree of engagement with decrease over time, while the high-risk trajectory of substance misuse had a medium initial degree of engagement with increase over time.

##### Influencing Factors

NSSI is maintained more frequently because of intrapersonal functions, such as affect regulation, self- punishment, and because of interpersonal functions, such as peer bonding, autonomy, that are especially relevant in youth with interpersonal difficulties (211, 213, 214).

Neurobiologically, adolescents with NSSI are less able to interpret social cues and regulate their emotions (215): female adolescents with NSSI but without BPD showed an increased activity in amygdala regions, the anterior cingulate cortex (ACC), and the inferior and middle orbitofrontal cortex, as well as a reduced sensitivity in the cuneus and right inferior frontal cortex during an emotional processing task.

ED moderates the longitudinal relationship between NSSI and disordered eating: Turner et al. (216) found a strong relationship between disorderd eating and later NSSI, together with high levels of ED but no significant moderating effect of ED for predicting concurrent NSSI and vice versa (217). Internalizing symptoms predicted NSSI in inpatient adolescent girls (218). Female adolescents with higher levels of depression had a higher risk of utilizing NSSI for regulating strong emotions (219). Both, ED and depressive symptoms, are related to the frequency of NSSI in adolescents [e.g., (127, 195)].

Environmental factors, such as stimuli that elicit emotional arousal, promote NSSI (209): there is a significant relationship between ED, interpersonal problems, and NSSI [e.g., (208, 220, 221)]. Interpersonal problems with the family and peers have independent negative effects on ED, with ED mediating the influence of interpersonal problems on the frequency and severity of NSSI. Higher levels of conflict and lacking support for ER in family and peer relationships went along with higher ED in adolescent girls hospitalized for psychiatric problems (208).

Children and adolescents with severe ED are at higher risk of NSSI when facing stressful life events (199). Especially child maltreatment increases the risk for NSSI (127, 222, 223). ED mediates the relationship between childhood maltreatment and the frequency of NSSI [e.g., (195, 223)]. Peh et al. (127) demonstrated ED to mediate the association between severity of child maltreatment and frequency of self-harm, while controlling for depressive symptoms in adolescent psychiatric outpatients. The authors considered exposure to childhood maltreatment as a distal, and ED as a more proximal associative factor, linking maltreatment exposure to self-harm.

A history of sexual abuse is strongly related to NSSI, particularly in girls. Chaplo et al. (141) investigated associations between sexual abuse and NSSI in traumatized delinquent youth on the basis of the dual mediating variables, ED and dissociation: higher levels of dissociation were associated with more frequent NSSI.

ER mediates the relationship between sexual orientation and NSSI in lesbian, gay and bisexual (LGB) adolescents. LGB youth are exposed to a greater risk of NSSI (224), probably related to bullying and peer harassment (225), and possibly parental rejection.

##### Suicidality

Suicidality is based on mental suffering, lacking self-respect, respect by others or of the feeling of being not loved by others (226). Erwin Ringel (227) described a presuicidal syndrome with 3 principal components, constriction, inhibited aggression turned toward the self, and suicidal fantasies (nowadays suicidal ideation, “escaping from a predicament”). The transition to suicide is characterized by the idea of hopelessness, feelings of anhedonia and severe anxiety, and direct planning of committing suicide (228). Suicidality is linked to depression but not exclusively (228). The risk of completing suicide increases with the number of suicide attempts, mental narrowing, and the emergence of a stressor, such as separation, loss of support. 90% of completed suicides are associated with psychiatric disorders, namely major depression and alcohol or substance abuse. The prevalences of suicide ideation, plans, and attempts are 2%, 0.6%, and 0.3% (229). The lifetime prevalence of suicidal ideation is 9.2%, and of attempted suicide 2.7% (230).

##### NSSI and ED

ER skills play a key role in the ability of adolescents to adequately identifying emotions, and helping them in selecting adequate coping strategies that may reduce suicidal ideation (231). Pan et al. (232) with the example of processing slightly angry faces, suggested that dysfunctions of the neural circuitry involved in processing emotions could smooth the path to suicidality in adolescents. Selby et al. (233) found ED to interfere with the ability to activate adequate emotional processing, and therefore would evoke suicidal thoughts. More specifically, ED is a well-established risk factor or even an underlying mechanism for suicidal ideation, suicide plans and suicide attempts in youth (130, 234, 235). The readiness for attempting suicide may be increased by impulsiveness and dysregulated behaviors (236). Comparing adolescents who attempted a number of suicides with those experiencing only one attempt, the former reported stronger deficits in ER and poorer impulse control (237).

#### Eating Disorders

Eating disorders (EatD) comprise anorexia nervosa (AN), with the subtypes, restrictive and binge-purging, bulimia nervosa (BN), and obesity (OB) (31, 238).

According to the affect regulation model, EatD serve as maladaptive coping strategies for pervasive emotional and behavioral dysregulation (239–242). ED is a key trans-diagnostic characteristic, arising from emotional vulnerability, combined with an invalidating familial environment that commonly can be targeted (243–245).

##### ED in Eating Disorders

Adolescents with EatD are significantly more impaired in their ability to regulate emotions compared to non-clinical samples [e.g., (244)]. In this study, patients with EatD scored higher in the Difficulties in Emotion Regulation Scale (DERS total score and subscale scores, “Non-Acceptance,” “Awareness,” “Strategies,” and “Clarity”). Symptoms were most strongly associated with “Strategies.” ED also plays a role in obesity, particularly among girls with self-reported loss-of-control (LOC) and binge eating (183). In contrast to individuals with adult-onset obesity, individuals with childhood-onset obesity showed a higher prevalence of EatD, particularly BN (246). The severity of an eating disorder relates to the severity of ED (241, 244, 247). High levels of parental ED are also associated with the severity of the children's EatD pathology (248). Maladaptive adolescent attentional bias toward anger and social threats predict a strong association between maladaptive parental responses to emotions and adolescent ED (249). There are specific emotional factors in developing and maintaining adolescent EatD:

- deficient ER strategies across a variety of domains (183, 241, 243, 250–257),
- “emotional” eating, defined as eating for emotional reasons in response to negative emotional states and in order to escape from negative affect (240, 251, 258, 259),
- poor emotional awareness (244, 250, 251, 257), and
- high and low approach on dysregulated positive emotions, such as avoiding positive affect and rewards (251).

Dysfunctional metacognitions represent another vulnerability factor for ED: Laghi et al. (257) found interaction effects between metacognitions and emotional functioning in binge eating adolescents. Metacognitions, the need to control thoughts, moderated the relationship between lack of emotional awareness and binge eating.

Jakovina et al. (256) found significantly higher levels of attachment related anxiety and avoiding strategies in adolescents with BN compared with controls, but only attachment related anxiety predicted BN symptoms, and was mediated by ER.

Monell et al. (244), comparing patients with various EatD subtypes, found only a few meaningful differences in relation to ED: patients with AN, binge-purging subtype showed more difficulties in controlling impulses than those with AN, restrictive subtype. Individuals with binge-eating disorders had higher impulsiveness scores than those with AN, restrictive or binge-purging subtypes, and EatD otherwise specified. The authors concluded that differences between EatD subtypes may depend on the study design, and possibly also on other factors than the eating disorder type. In contrast to Monell et al. (244), other authors [e.g., (255, 260)] found higher ER deficits in individuals with BN and binge-purging AN compared to individuals with restrictive AN. Across ages, Anderson et al. (255) found less self-reported acceptance of emotional responses, higher impulsiveness, fewer ER strategies and low emotional clarity (2) in patients with AN binge-purging type or BN, whereas patients with restrictive AN showed more goal-directed behaviors in stressful situations than those with BN, and a better awareness of emotions than in those with binge-purging AN.

##### Influence of Emotional Child Abuse

Emotional child abuse may induce the development of severe ED and severe eating disorder (EatD), including AN. A number of studies support the strong psycho-pathological relationship and long-term comorbidity of the two disorders (252, 261). ED mediates the relationship between emotional child abuse and AN. Nature and magnitude of this influence do not differ regardless of the AN subtype (252). AN is also associated with higher levels of comorbid depressive or anxiety disorders, OCD, PTSD, and interpersonal problems. Individuals with binge-purging AN had experienced more severe maltreatment, neglect (261), and sexual abuse (252) than those with restrictive AN. McDonald et al. (262) found binge-purging AN to co-occur more frequently with BD than with restrictive AN. Patients with BD and EatD usually are more impulsive and have more severe EatD. They also suffer more from alcohol and substance abuse, suicidality and mood instability than patients with BD only. Slane et al. (263) investigated monocygotic twins with dysregulated BN and comorbid alcohol use disorders at ages 17 and 25 years. They found non-shared environmental effects (i.e., factors that create differences in monocygotic twins) that did not influence the association between BN and alcohol use disorder.

#### Oppositional Defiant Disorder, Conduct Disorder, and Disruptive Mood Dysregulation Disorder

##### Oppositional Defiant Disorder

Oppositional Defiant Disorder (ODD) is a disruptive behavior disorder (DBD) of childhood and adolescence that can be described as recurrent, persistent, developmentally inappropriate patterns of anger, irritability, negativity, defiance, disobedience and deliberate hostility toward others, resulting in functional and social impairment (31). Children with ODD commonly experience dysregulated emotions such as temper tantrums, intense fears, inconsolable despair, problems to feel and express emotions, and a low tolerance to frustration (264), co-occurring with externalizing behavior problems (265, 266).

For these children, emotions seem uncontrollable or absent. They tend to think simplisticly, rigidly, and reactively, and are led by defiance and aggression (264). Adolescents with ODD and higher scores on the Child Behavior Checklist, Dysregulation Profile (CBCL-DP) possess poor abilities to regulate affect, behavior and cognition, and are more likely to present with auto-aggression (267). ODD is associated with impairments of social, academic, occupational and family relationships over the lifespan (268). Most studies concentrate on the association between ED and externalizing behavioral problems (269, 270). Studies on the relationship between ODD and ED are lacking, except for a few studies suggesting a strong association between ED and ODD (270–273). It is unclear if ODD criteria are uni-dimensional, if ODD is better conceptualized as an ER disorder (271), or if ODD is a multidimensional construct and better conceptualized as a disorder of mood regulation (273, 274).

##### Conduct Disorder

Conduct disorder (CD) is a behavioral and emotional disorder characterized by functional impairment that includes intentional violations of the rights of others, societal norms or rules (31). Children with CD typically show aggressive, antisocial behavior, and callous-unemotional (CU) traits including low prosocial emotions and behaviors, such as blunted affect, lack of guilt, physiological under-arousal, and lack of empathy (275). Antisocial behavior of children with ODD or CD have partly been explained by deficits of information processing and ER. The combination of misinterpreting social cues in a negatively biased and stereotypic way, limited strategies for coping with anger, and lack of behavioral control, especially difficulties in response inhibition, lead to inappropriate handling of distressing emotions and impulsive behavior (264, 273). Fehlbaum (275) investigated adolescents with CD in a controlled fMRI study.

Both groups were confronted with an emotional stimulus and a Stroop task with varying cognitive load. Adolescents with CD made significantly more errors, while reaction times were not significantly different compared to typically developing (TD) youths. In children with CD, left amygdala activity failed to be down-regulated in response to incongruent trials, and anterior insular activity increased during the Stroop task. The authors concluded that children with CD could not adequately process distracting emotional information and suppress impulsive thoughts, leading to antisocial behavior. They also concluded that rather their neurological problems than ED was responsible for their inappropriate behavior.

Mitchison et al. (273) examined the relationship between ED, ODD symptoms and conduct problems in preschool children: problematic behavior occurred more often at home than in the kindergarten setting, and there was a strong relationship between ED, ODD symptoms and conduct problems especially regarding lability/negativity. Boys had more severe ED problems than girls. Furthermore, ED was found to be a strong, gender-independent predictor of ODD symptoms and conduct problems. This is also supported by the work of Schoorl et al. (276). Multimodal extensive treatment is recommended including socio-therapy, individual and family psychotherapy, and medication. Methodologically sound controlled trials are still lacking (265).

##### Influence of Comorbid Disorders

ODD is very frequently (almost 50–60%) comorbid with ADHD (277). Children with ADHD and comorbid ODD showed significantly more negative emotional lability compared to children without ODD, involving impairment in the regulation of a variable, intense pattern of emotional responses (278).

There is an association between early traumatic experiences and later aggressive, impulsive and antisocial behavior [e.g., (279–281)].

Another study focused on CU traits, manifesting as a consequence of traumatic experience and resulting deficits in ER. Adolescents with high expression of CU traits showed impaired emotional responses and exhibited severe aggressive-dissocial behavior. Potentially traumatized adolescents with highly expressed CU traits showed significantly more external-dysfunctional ER strategies than traumatized adolescents with low CU expression (282).

Investigating incarcerated adolescents, Sevecke et al. (111) observed ED and psychopathic traits to occur only in boys. Hoskins et al. (283) found past trauma exposure in three quarters of first-time offending, court-involved, non-incarcerated Latino youth. Traumatized girls presented with more severe internalizing symptoms and affect dysregulation, traumatized boys with more externalizing symptoms.

ED and antisocial behavior are commonly observed in juvenile offenders, depending on the co-occurrence of emotional neglect. Physical abuse in incarcerated boys was related to ED only in those with co-occurring emotional neglect (117).

ED was associated with more severe aggressive behavior in urban adolescent boys and girls who witnessed community violence (284). Ford (264) developed a three-step model explaining the relationship between trauma and victimization in childhood:

1. “survival coping” characterized by dysregulation of emotions and deficient social information processing (77)
2. “oppositional-defiant” behavior, involving covert or overt aggression and PTSD
3. “victim coping” in a chronological sequence.

Plattner et al. (285), comparing delinquent adolescents and high-school students, observed that delinquents had higher levels of negative emotions (fear, sadness, and anger) as state and trait conditions, probably linked to childhood trauma experience. The duration of trauma exposure influenced trait emotions, and the severity of trauma (emotional abuse and witnessing violence) had an impact on state emotions. When stressed, delinquent adolescents showed more state emotions of sadness, anger, and a wider range of negative emotions.

There are also associations between deficient executive functions and ADHD: Landis et al. (286) reported that children with dysexecutive problems (operationalized by a questionnaire and a well-established survey tool) were classified as more inattentive and hyperactive. Both, hyperactivity and inattention, were associated with ED.

##### Disruptive Mood Dysregulation Disorder

A new and controversial disorder of children similar to ODD is Disruptive Mood Dysregulation Disorder (DMDD) (31). The disorder is characterized by severe, chronic, non-episodic irritability, frequent temper tantrums, and verbally or behaviorally expressed outbursts that are disproportionate to the trigger and inappropriate to their developmental level. Children with DMDD generally present annoyed, touchy, and persistently angry, with mood swings and irritability (287–289). They have poor ER abilities and frequently lose behavioral control, contributing to rising frustration and distress (290). It remains controversial whether DMDD is a unique entity or if it is closely related to ODD (290, 291). According to Dougherty et al. (292), a DMDD diagnosis is associated with concurrent and predictive indicators of emotional and behavioral dysregulation, and poor social functioning. They also noted that temperamental surgency, a construct reflecting high levels of activity, reward seeking, low shyness and impulsiveness, of 3-year old children predicted DMDD at the age of 6. Zepf et al. (293) reported about diminished cognitive flexibility in children with DMDD, assessed by a reversal learning task (294), and poor motor inhibition (295).

#### Personality Disorders

ED plays a substantial role in personality disorders (PD) with the majority of research focusing on borderline personality disorder (BPD). We only found a few recent studies, investigating ER problems in adolescents with PDs other than BPD. Compared with healthy controls, children with obsessive compulsive personality disorder exhibit more alexithymia (296), impulsivity, behavioral activation (297), and poorer effective ER strategies (296, 298).

##### Borderline Personality Disorder

BPD is a serious mental illness that includes ED and interpersonal problems. Based on the bio-psycho-social developmental model of BPD (80) there is a predisposition toward increased emotional sensitivity and intensity of responses to emotional stimuli with a slow return to baseline following emotional responses, and adverse social influences. ED mediates the relationship between BPD, emotional vulnerability (299), and over-mentalizing (excessive inaccurate mentalizing, i.e., excessive Theory of Mind, TOM) (300). In accordance with Linehan (80), Carpenter and Trull (301) conceptualized ED in BPD as consisting of four components: 1. emotional sensitivity, 2. heightened and labile negative affect, 3. deficits of appropriate regulation strategies, and 4. excessive maladaptive regulation strategies.

BPD and ADHD share a number of common features, such as impulsiveness, ED, deficits in attention and decision-making, comorbid major depression; brain volume reductions and impairments of connectivity in prefrontal, anterior cingulate and limbic areas (302, 303).

Results of studies assessing neural correlates of ED in adolescents with BPD are heterogeneous. Comparing female adolescents with BPD and healthy controls, Krauch et al. (304) found increased activation in the left posterior insula and left dorsal striatum as well as in the inferior frontal gyrus and parts of the mentalizing network. This suggests an enhanced emotional reactivity to interpersonal threat- or rejection-related situations early in the development of BPD.

Attachment, ED, and BPD are strongly interrelated [e.g., (80, 305, 306)]. Disordered attachment plays a significant role in the pathogenesis of BPD (307, 308). Maternal ED mediates the relationship between maternal BPD and child functioning (309). There is an interplay between disordered attachment and features of BPD mediated through ED (310). Secure attachment to the father functioned as a buffer against adolescent BPD via enhanced positive ER strategies, while negative ER strategies served as a pote correlate of clinically significant levels of BPD, weakening the protective effects of attachment and positive regulation strategies.

Genetic and environmental effects are likely to influence attachment patterns and personality disorders (311). In monocygotic twins, the associations between self-reported anxious attachment (i.e., fears of abandonment and difficulties in regulating worries about the availability of attachment persons) and PDs were mostly explained by genetic factors, while self-reported avoidant attachment (i.e., discomfort with close relationships and depending on others) was entirely influenced by non-shared environmental effects. Factor analyses revealed that anxious attachment loaded on ED, while avoidant attachment loaded on inhibitedness. Attachment anxiety correlated with affective lability and self-harm (characteristic for BPD), increased self-satisfaction (characteristic for narcissistic PD), oppositionality, submissiveness, and the lack of self-fulfillment (characteristic for identity problems). The authors suggested that probably different sets of genes contribute to the specific associations observed between these variables to explain why anxious attachment correlates with different psychopathologies. Neither avoidant nor anxious attachment showed any relationship with PD scales indexing dissocial behavior or compulsivity, suggesting that these dimensions of personality pathology are not related to attachment styles (311).

More severe features of BPD are significantly associated with increased hypermentalization, ED, and internalizing or externalizing symptoms (312). Hypermentalization and ED mediates the relationship between attachment coherence and features of BPD. Hypermentalization and ED are independently related to BPD. Kalpakci et al. (313) investigated relations between ER, hypermentalization (i.e., incorrect, over-inference of thoughts and feelings of self and others), and cognitive and affective empathy in inpatient adolescents with and without BPD. In both groups, ED was related to increased affective empathy.

There are differences between BPD and healthy controls in information processing (80, 314): patients with BPD showed alterations in early validation processes that determine the emotional response and trigger ED (315). Analyzing patterns of emotional responses to stimuli, patients with BPD showed significantly greater arousal and greater valence (more positive emotions) than healthy controls when looking at unpleasant and neutral images, but lower dominance (greater insecurity and discomfort) when looking at positive images. These results are similar to the pattern found in depressive patients (315).

Features of BPD were associated with significantly higher levels of experiential avoidance (i.e., the unwillingness to remain with uncomfortable thoughts, emotions, sensations, memories and urges by escaping or avoiding them), and difficulties in ER. Experiential avoidance partially mediated the relationship between difficulties in ER and features of BPD. The authors suggested a reciprocal relationship between ER and features of BPD - difficulties in ER being associated with both, experiential avoidance and features of BPD (316).

Trait impulsiveness and the three dimensions of ED (difficulties in controlling impulsive behaviors when distressed, limited access to effective ER strategies, and lack of emotional clarity) were significantly associated with BPD features in two independent non-clinical samples of Italian adolescents (317).

Aggressiveness most substantially differentiated between patients with and without BPD. Parents rating adolescents on BPD scales described them as presenting with more anger, hostility, and indirect aggression (318). ED and trait anger sequentially mediated the association between BPD and reactive aggression, generated by increased interpersonal threat sensitivity (319, 320). Banny et al. (321) observed features of BPD predicting increases in reactive (i.e., impulsive/dysregulated) relational aggression and proactive (i.e., premeditated/controlled) relational aggression, and decreases in proactive physical aggression in girls 1 year later. Measurements of systolic and diastolic blood pressure, and skin conductance reactivity supported the hypothesis that aggression is a strategy for girls with features of BPD to cope with overwhelming intense negative affect in the context of ED, more precisely in response to stressful peer interactions (threats or exclusion).

Yen et al. (318) compared suicidal adolescent inpatients with and without BPD. They found that suicidal patients with BPD had more Axis I co-morbidities, higher levels of aggression, and a greater likelihood of a history of serious suicide attempts. There were no significant differences in ED between the two groups. The authors suggested that affective dysregulation may be more trans-diagnostic and not specific for BPD, particularly in a high-risk sample of suicidal adolescents.

There is a close relationship between interpersonal trauma experience and PD (87). ED is a consequence of exposure to direct or indirect physical or sexual violence associated with posttraumatic stress symptoms [e.g., (184)] and BPD pathology [e.g., (322, 323)]. Ford and Courtois (324) provide an extensive summary of the role of trauma and ED in BPD. Buckholdt et al. (172) examined the mediating role of ED in the relation between exposure to violence and PTSD or BPD pathology in adolescents. They found that patients exposed to violence presented with more ED, which, in turn, was related to more PTSD and BPD pathology. ED mediated the association between exposure to violence and PTSD or BPD pathology.

For BPD and NSSI, please refer to the section on Eating Disorders. Frequently observed comorbidities of BPD are bipolar disorder (BD), ADHD, and disordered sleep. The number of BPD symptoms is correlated with the severity of BPD (325). The BPD factors, affective dysregulation, involving affective instability, fear of abandonment, and inappropriate anger, is associated with BPD chronicity and severity. In addition, threat sensitivity and impulsivity in the context of negative affect were related to a higher risk of BPD (65). Preliminary research indicates that patients with BPD and comorbid sleep disturbance have an increased risk of suicidality [e.g., (326)], suggesting impulsivity and ED as potential psychological mechanisms driving the insomnia – suicide link in BPD.

There is a strong negative relationship between personal life objectives, ED, and BPD. Patients with BPD have a lower feeling of meaning in life than mentally handicapped individuals without BPD. Marco et al. (327) designed a multiaxial model, consisting of the axes, “ED,” “emotional suppression,” “satisfaction and meaning in life” (subscale and overall score), and “personal life objectives.” The model explained more of the variance in BPD symptoms than the ED scales alone.

##### Narcissistic Personality Disorder

Narcissistic personality disorder with its various manifestations is associated with ED beyond personality characteristics and constitutes an important factor in the psychopathology of the disorder (328, 329). The latter authors studied 1,018 undergradutate students, based on the narcissitic admiration and rivalry concept model and the related questionnaire (330). They found that merely narcissitic rivalry was associated with problematic responses to and poor recognition of emotions, whereas persons with the admiration variant could regulate emotions more effectively.

##### Psychopaths

Higher levels of psychopathic traits were assiociated with increased brain tissue volumes in the left putamen, left ansa peduncularis, right superiomedial prefrontal cortex, left inferior frontal cortex, right orbitofrontal cortex, and right medial temporal regions, and reduced brain tissue volumes in the right middle frontal cortex, left superior parietal lobule, and left inferior parietal lobule (331).

#### Substance Use Disorder

Adolescents with ED are at high risk for substance use and misuse disorder (SUD) (332). Adolescents with ED start misuse earlier, and transition more rapidly into SUD. Longitudinal studies identified externalizing symptoms in early adolescence predicting SUD in late adolescence and early adulthood [e.g., (333)]. Differences in brain cortical thickness have been described in association with problems of ED, inhibition, and behavioral control in female adolescents with SUD (334). Disturbance of the endocannabinoid signaling in the amygdala-prefrontal cortical circuit may lead to abnormalities in the processing of emotionally salient information, learning, and memory (335). ADHD has been identified as a risk factor for early substance use [e.g., (336)]. Disturbances of the circadian rhythm and sleep are associated with affect dysregulation, increased drug and alcohol misuse, and other risky behaviors in adolescents (337). Poor emotional control is related to the frequency of alcohol, marijuana and cigarette use in adolescence, mediated by proximal influences like exposure to negative experiences and social motives for substance use (332). Consistent with these observations, affect dysregulation is associated with a history of misusing various substances, including marijuana, alcohol, cocaine and downers (338, 339). This may indicate a general tendency to engage early in risky behaviors, being more susceptible to peer influences, or attempting to control emotions by substance use (340).

Although negative sequelae have to be feared, adolescents engage in binge drinking because they are emotionally, behaviorally, or cognitively dysregulated (113). Cortical thickness - to be specific, thinner dorsolateral prefrontal cortex and inferior frontal cortex in early adolescence - is predictive of binge drinking and externalizing symptoms in late adolescence (341). Theory and research about emotionally labile youth suggests that lacking of internal regulation resources is frequently associated with exposure to external maladaptive coping strategies, such as alcohol misuse (342). This is consistent with self-medication theories where consuming alcohol serves as a coping strategy for overcoming negative emotions (343).

Marijuana use (occasional, heavily increasing, chronic) is associated with ED, nicotine and alcohol abuse and dependency (344). Adolescents with poor ER strategies may be prone to regular use of marijuana (345, 346) that may impair cognitive abilities and emotional reactivity. Marijuana and alcohol use are associated with white matter disorganization, which in turn predicts ED (347, 348).

Nicotine dependence among adolescents is a widespread health concern (349). ED predicts adolescent smoking behaviors (342, 350), and SUD (351). High levels of distress combined with ED may predict smoking-naive adolescents to develop positive expectations about social acceptance with smoking. This may promote the decision to start smoking [e.g., (352, 353)]. Longitudinal data suggest that low levels of ER predict initial adolescent attempts to start smoking, as well as the transition to regular smoking (350). The higher the ED the higher the risk of smoking (338). Following the self-medication model of Khantzian (343), poor regulation of negative affect (especially of anger) increases the vulnerability to smoking and SUD [e.g., (354, 355)]. Adolescents may engage in cigarette smoking or substance use, in order to cope with anger-related distress (355). Personal motives, such as reducing negative affect, are among the most common reasons for cigarette smoking [e.g., (356–358)], a process that - besides nicotine dependency - reinforces and maintains smoking behavior (359). Padovano et al. (360) found high positive or negative arousal related to smoking relapse, again highlighting the importance of affective dysregulation as a risk factor for adolescent smoking. Therefore, smoking prevention and intervention programs for high risk adolescents should include practicing cognitive and behavioral ER strategies [e.g., Contextual Emotion-Regulation Therapy; (361)].

Recent studies identified ED as a mediator between drug abuse and SUD (184, 362). As an example, the (weak) opioid agonist, tramadol, is misused in order to enhance positive mood and to perceive pleasant emotions (363). Ghorbani et al. (137) suggested that lacking ER strategies may be related to heroin craving in individuals with heroin dependence who experienced a history of CT.

Prenatal cocaine exposure is associated with long-term dysregulation of arousal (364). Neuroimaging data confirm these observations by findings that prenatal cocaine exposure has deleterious long-term effects on the arousal regulation system (365).

#### Developmental Disorders Including Autism Spectrum Disorder

Autism spectrum disorder (ASD) is defined as a neurodevelopmental disorder, characterized by impaired social communication and interaction as well as repetitive behaviors or restricted interests (31). ASD also goes along with various emotional and behavioral difficulties, such as ED (366–368). Although ED is not considered a core feature of ASD, the prevalence of ED in the context of ASD is up to 50 or 60% (369, 370), significantly higher than in other clinical disorders or in children with normotypical development (291, 369–376). In general, ER problems manifest especially in a more frequent use of maladaptive ER strategies, such as avoiding (377), or a less frequent or ineffective utilization of adaptive strategies for regulating emotional states (98, 367, 368, 378, 379). ED often leads to increased problems of social adaptation, school related problems (380, 381), higher rates of social and general anxiety (382–384), and other difficulties (385–387). Moreover, ED is associated with higher rates of repetitive behavior, communication and social skills deficiencies (370, 371). Owing to the intensifying effect of ED on ASD specific symptoms, various studies aimed at identifying predictors of this co-occurrence (366, 370, 371, 375, 380, 388–390).

Gadow et al. (380) described a modulating effect of dopaminergic polymorphisms on ED in the context of ASD. Fenning et al. (388) suggested problems of internalizing and generalizing parental ER strategies to promote ED in ASD patients. This assumption is supported by observations that parental scaffolding helps children in overcoming frustrating situations and in improving ER. Children with ASD seem to benefit less of this support (372, 388). Samson et al. (98, 379) found that therapy programs focusing on teaching adaptive ER strategies improved both, ED and ASD-related symptoms. There are also observations that pharmacotherapy with the ß-blocking agent, propranolol, acting on the consolidation of anxiety memories, improves problem-solving skills and ED in children with ASD (274). Thus, approaching ED may improve the complex association of ASD and ED (390–392).

The diagnosis of ED may be facilitated by using specific inventories. Mazefsky et al. developed and evaluated the Emotion Dysregulation Inventory for assessing deficient ER in the context of ASD (391, 392). Future research should focus on this interesting topic because specific diagnostic and therapeutic instruments are still scarce (370).

#### Psychosis and Schizophrenia

Schizophrenia is considered a “severe and persisting brain disorder” (393) with psychotic symptoms that may occur as a single episode or (in the majority of cases) as recurrent chronic disorder. The lifetime prevalence of schizophrenia is ~1%, children and adolescents having a lower risk of 0.018% (393–395). Schizophrenia usually goes along with intrusive thoughts, impaired cognitive functions, marked changes of personality, and symptoms of ED (97, 393, 396).

The clinical characteristics of schizophrenia are manifold, symptoms may be non-specific. Usually positive (such as hallucinations, delusions) and negative symptoms (such as affective flattening, avolition, catatonia) are distinguished (397). Symptoms are not exclusively characteristic for schizophrenia but have been refined since the development of the DSM-IV. Symptoms may vary with age and also depend on comorbid problems, such as affective dysregulation. Jerrell et al. (393) described symptoms of ED, especially in older adolescents needing in-ward treatment. ED has a moderating effect on the severity of schizophrenia associated “positive” symptoms, such as acoustic and optic hallucinations in relation to the severity of ED (398–400). In addition, subclinical changes of personality seem to be associated with more accentuated manifestations of ED. For example, pronounced schizotypal personality may be considered a subclinical stage of schizophrenia and associated with a higher risk of developing schizophrenia (401–405). This close relationship between ED and schizophrenia or schizotypal disorder suggests a genetic link for this co-occurrence (405). This could help improving the specificity and sensitivity of diagnostics of ED in schizophrenia, and especially in adapting multimodal treatment approaches individual needs (402).

#### Gaming Disorder

The use of electronic media (computers, internet, video and mobile phone use) has dramatically increased in the last two decades, especially in children and adolescents. Daily electronic media use has increased to a mean of 7.4 h in 8–18 year old adolescents (406). The gain in entertainment and knowledge acquisition is opposed to reductions of real social and physical activities, and has a negative impact on physical and mental health. ER abilities also become compromised (407). Available data are, however, limited, especially for children and adolescents.

Adolescents having difficulties in regulating their emotions, are especially prone to problematic technology use (408, 409). The association between ED and problematic internet use (more than 4 h/day) is partly mediated by meta-cognitions in young adults (410). Donald et al. (411) found ED to be correlated to the amount of problematic internet use. Playing internet games is used to regulate unpleasant emotions. In addition, escapism is another important motivator for excessive gaming (412). Problematic internet use promotes ED, such as difficulties in recognizing emotions and goal setting.

In addition to problematic internet use, the use of video and computer games is also increasing. The median prevalence of internet gaming disorder is about 2% for children and adolescents (413). Excessive use of computer or video games leads to impairments or distress depending on the amount of activity. As early as in the preschool age, the presence of ED has been shown to be a predictor of media use and GD symptoms 5 years later (at about age 9) (414). Difficulties in impulse control and a limited access to emotions is associated with problem video gaming (415, 416). Wichstrom et al. (417) showed that ER deficits in 8 year olds predicted symptoms of internet gaming disorder at 10 years.

### Prevention and Treatment

In the following, we will describe meaningful preventive and therapeutic strategies for improving ER or ameliorating ED. Because of the complex background of ED, prevention and therapy will primarily focus on recognizing risk factors such as personality, familial and social conditions, underlying disorders, such as ADHD, affective or trauma related disorders and and their comorbidities. Especially familial factors will be susceptible to parental training, youth welfare support, and in case of not sufficiently improvable conditions, early placement in suitable foster families may be necessary.

Therapies for treating underlying disorders, such as medication for ADHD or affective disorders, for decreasing internal tension or improving stability and reactivity may be necessary. In addition, building-up or improving sensitivity and appropriate coping strategies in social or self-regulatory skills training will be beneficial (see below, section Treatment). In the following, we will describe specific measures and strategies that have proved to be successful.

#### Prevention

Prevention of ED includes identifying risks and learning or applying appropriate measures in order to prevent damage. This has been shown e.g., by treatment of postpartum depression (418), positive parenting programs (419), or early placement in care families or adoption of children from maltreating families (cf. chapter Psychological Trauma). The results of such preventive measures to date are disappointing (too late, too inefficient, too many children missed) (420). In fact, only a few studies reported about effective prevention methods.

Adolescents attending alternative schools because of behavioral and emotional problems, and youth at odds with the law have difficulties in managing strong emotions. This may also concern their sexual behavior. Effective Human immunodeficiency virus (HIV) prevention programs for adolescents should include the training of keeping a cool head dealing with strong emotions in sexual relationships. Affect management skills for reducing the risk of HIV infection are comparable to techniques used in dialectical behavioral therapy (DBT), and have successfully been applied (421, 422).

Teachers working in schools with socially and economically disadvantaged children have been trained for improving their children's social skills and emotional self-regulation, to reduce their conduct problems, and involve the parents in their supporting role. Results indicate that teachers in the intervention group used more positive classroom management strategies, their students were better in applying social skills and emotional self-regulation, and had less conduct problems compared to the non-intervention control group (423).

Transdiagnostic interventions for internalizing disorders target common underlying mechanisms and may attract a larger proportion of these youths than concepts developed for single disorders (424). As an example, the recently developed CBT-based transdiagnostic prevention program, EMOTION (425) for internalizing disorders, includes techniques for improving children's ER skills, psychoeducation, behavioral activation, cognitive restructuring, building of a problem hierarchy and exposure to feared or until now avoided situations. The 10 weeks lasting program has been shown to improve children's ER skills.

Deplus et al. (426) tested an intervention adapted from mindfulness-based cognitive therapy aiming at enhancing self-regulation skills in adolescents. The nine sessions' program was well-accepted and increased self-reported mindfulness. In addition, depressive symptoms, impulsivity (dealing with urges, and lack of perseverance), and dysfunctional strategies of ER improved.

Guendelman et al. (427) extensively reviewed mindfulness-based therapies from a neurobiological, psychological and clinical perspective. They reported changes in ER based on clinical and functional data (e.g., decreased activation changes in the amygdala, hippocampus, anterior insula, anterior cingulate cortex), following mindful meditation even in novices. We found only one randomized trial (428) comparing hypnotherapy and self-care in juvenile post cancer patients, showing evidence for the efficacy of hypnosis in improving ER. Targeted real-time fMRI-neurofeedback, downregulating amygdala activation, may induce longterm improvements of ED in patients with PTSD, BPD and schizophrenia (429).

#### Treatment

ED symptoms occur in many psychiatric disorders, often as promoting or comorbid condition. Since ED may significantly influence the development of children and adolescents, effective and available treatment is of utmost importance. Psychotherapy may be divided into four groups: (i) Dialectic Behavioral Therapy [reviewed by Courtney-Seidler et al. (430)], (ii) Behavioral and Cognitive Behavioral Therapy (including Schema Therapy) [recently reviewed by López-Pinar et al. (431)] in adults, (iii) multimodal treatment (including, e.g., parental interventions, pharmacotherapy, and others), (iv) other therapies, such as analytic group therapy, hypnotherapy, neurofeedback, and others.

Garrett et al. (432) studied pre-post effects of 4 months psychotherapy in youth with ED and a risk of BD in an fMRI setting, presenting a facial expression task. At baseline they found hypoactivation of the dorsolateral prefrontal (DLPFC) and posterior cingulate cortex compared to matched healthy controls.

Following treatment, activation of the DLPFC increased, and decreased in the amygdala, paralleling the improvement of symptoms.

Dixius and Möhler developed a low threshold program START showing positive impact on ER in traumatized teenagers (433). Thornback and Muller (91) investigated the relationship between improvement of ER and improvement of trauma related symptoms in children receiving trauma focused cognitive behavior therapy: improvement in ED was associated with improvement in the child's internalizing, externalizing, and PTSD symptoms. Mindfulness based group therapy can improve ER in children with ADHD (41, 434).

Because ED is very common, especially in children and adolescents (although only in the focus in the last decade), various treatment options primarily concentrate on this dimension (426, 435–444).

Mode Deactivation Therapy [MDT; (436, 438)], and Modular Approach to Therapy for Children with Anxiety, Depression, Trauma, or Conduct Problems [MATCH; (443)] were particularly effective. MATCH, a cognitive behavioral therapy, improves ED faster than other programs. Apsche et al. (436) and Bass & Apsche (438) described an extended version of cognitive behavioral therapy (MDT) superior to conventional cognitive behavioral therapy, especially in reducing anger and aggressive behavior in adolescents and juvenile offenders (435) (see Tables 1, 2). Since up to 90% of children with ED meet criteria for a categorized disorder (441), a number of treatments are available for treating the primary disorder. Most therapies also help in improving ED (91, 100, 164, 210, 258, 430, 432, 445–448, 450–457). Only the Light Therapeutic Procedure (447), and the Emotion Regulation Training (ERT) for adolescents with BPD (455) showed no improvement (for details, see Tables 1, 2).

**Table 1:** Clinical trials focusing on ED as primary target.

**References**	**A: primary target,** **B: secondary target**	**Treatment**	**Age groups** **participants (n)**	**Results**
Apsche et al. (436)	A: ED, B: -	MDT vs. CBT	Male adolescents (14–18 years) *n* = 84	MDT was more effective than CBT for the treatment of externalizing and internalizing disorders & ED. MDT especially reduced the dysregulation of anger and aggression in male adolescents to a greater extent
Bass et al. (438)	A: ED, B: -	MDT	Male adolescents (14–18 years) *n* = 84	Replication of findings of Apsche et al. (436)
Deplus et al. (426)	A: ED, B: Depression; impulsive behavior	Mindfulness-based CT	Adolescents (11–19 years) *n* = 21	Reduction of depressive symptoms, impulsive behavior and ED
Dixius and Möhler (433)	A: ED B. PTSD	START	Adolescents (13–17) *n* = 66	Treatment significantly improved emotional regulation
Döpfner et al. (441)	A: ED, B: -	ADOPT	children (8–12 years) *n* = 597	Versions of ADOPT (i.e., ADOPT online/institution etc.) were associated with an improvement of ED symptoms
Ducharme et al. (437)	A: ED, B: -	DBT, CBT	Children & adolescents (9–17 years) *n* = 37	Reduction of anger scores caused by decrease of ED
Evans et al. (443)	A: ED, B: -	Match (BT) vs. other treatments	Children & adolescents (7–13 years) *n* = 175	General improvement of ED symptoms. MATCH led to faster decrease of ED compared to other standard treatments; ES = 0.49
Pardo et al. (444)	A: ED, B: impulse control disturbance	DBT	Adolescents (Ø = 15.4 years) *n* = 20	General improvement of ED and symptoms of impulse control. Qualitative reports of adolescents: positive statement of adolescents who terminated the treatment program
Ravindran et al. (439)	A: ED of parents, B: -	MFWSB	Parents of children (4–8 years) *n* = 84	Reduction of ED only in mothers of children
Simpson et al. (442)	A: ED, B: -	Mindfulness-Based Intervention	male incarcerated youth (18–21 years) *n* = 48	Improvement of ED, sleeping quality, stress-level etc.; ES (impulsivity) = 0.72, ES(mental well-being) = 0.50, ES (inner resilience) = 0.35, ES (mindfulness) = 0.32
Thoder and Cautili (435)	A: ED, B: -	MDT	Juvenile offenders (14–17 years) *n* = 39	Decrease of internalizing and externalizing symptoms (i.e., ED). Reduction of aggressive and delinquent behavior. Positive effects on relapse risk
West et al. (440)	A: ED, B: -	Sensory room	Adolescents (12–18 years) *n* = 112	Reduction of stress level, especially aggressive behavior, general improvement of ED symptoms
Winiarski et al. (445)	A: ED, B: -	MST	Adolescents (12–17 years) *n* = 180	Decrease of physiological and behavioral indicators of ED, significant differences between male and female adolescents: females had higher responder-rates

*ADOPT, Affective Dysregulation–Optimizing Prevention and Treatment; BT, Behavioral Treatment; CBT, Cognitive Behavioral Treatment; DBT, Dialectic Behavioral Treatment; ERIC, Emotion Regulation and Impulse Control Treatment; ERT, Emotion Regulation Training; ES, effect size; IGST, Individual / Group Supportive Therapy; MATCH, Modular Approach to Therapy for children with anxiety, Depression, Trauma or Conduct Problems; MDT, Mode Deactivation Therapy; MFWSB, More Fun with Sisters and Brothers; MIT-G, Metacognitive Interpersonal Therapy in Groups; MMT, Mindfulness Meditation Therapy; MST, Multisystemic therapy; START, Stress-Trauma-Symptoms-Regulation-Treatment; TAU, Treatment as Usual*.

**Table 2:** Clinical trials focusing on ED as secondary target.

**References**	**A: primary target, B: secondary target**	**Treatment**	**Age groups participants (n)**	**Results**
Adrian et al. (450)	A: NSSI & suicidality, B: ED	DBT vs. IGST vs. other treatments	Adolescents (Ø = 14.89 years) *n* = 99	DBT appears to be more effective for the treatment of suicidal adolescents with higher levels of ED than IGST
Bjureberg et al. (210)	A: NSSI, B: ED	ERITA online (BT)	Adolescents (13–17 years) *n* = 25	After treatment reduction of NSSI and ED in adolescents & improvement of parental adaptive behavior ES (past month NSSI frequency) = 0.88, ES (global functioning) = 1.01, ES (ED) = 0.75, ES (NSSI versatility)= 0.63 (number of different types of NSSI behaviors)
Blader et al. (447)	A: ADHD, B: DMDD	Family-based BT	Children (6–13 years) *n* = 156	Reduction of DMDD symptoms. Decrease of aggressive behavior in 51%
Bogen et al. (448)	A: Depression, B: ED	Light therapy	Adolescents (12–17 years) *n* = 57	No improvement of ED, but ED could eventually be enhanced by amelioration of sleep & circadian rhythm; partial *Eta*^2^(sleep quality) = 0.02, partial *Eta*^2^(restorative sleep) = 0.09, partial *Eta*^2^(circadian preference) = 0.22
Boutelle et al. (258)	A: Eating Disorder, B: ED	PEER (BT)	Adolescents (13–17 years) *n* = 53	Significant reduction of emotional eating situations. Trend toward reduction of ED; ES = 0.32
Ford et al. (449)	A: PTSD, B: ED	TARGET vs. ETAU	Female delinquent adolescents (13–17 years) *n* = 59	Both therapies reduced anxiety, anger, depression, and posttraumatic cognitions (medium effect sizes). Interaction effect between TARGET and time with respect to PTSD, anxiety, posttraumatic cognitions, and emotion regulation
Ford et al. (473)	A: PTSD, B: ED	TARGET	Detained adolescents (11–16 years) *n* = 394	TARGET was associated with fewer disciplinary incidents and seclusion
Garrett et al. (432)	A: Depression/Mania, B: ED	Multimodal therapy	Adolescents (13–17 years) *n* = 24	Improvement of mood dysregulation was associated with increased activation in DLPFC, decreased activation in amygdala, and reduced maniac symptoms; ES(maniac symptoms) = 0.59, ES(CDRS) = 0.56
Goldstein et al. (451)	A: Bipolar Disorders, B: ED	DBT	Adolescents (12–18 years) *n* = 10	Significant improvement of all symptoms (NSSI, suicidality, depressive, maniac, and ED symptoms) ES(ED) = 0.3
Heinrich et al. (452)	A: ADHD, B: ED	Neuro-feedback	Children (8–12 years) *n* = 30	Improvement of ED symptoms & cognitive and behavioral dysregulation. Decrease of ADHD specific symptoms,
Kaufman et al. (453)	A: Self-injury, B: ED	DBT	Female adolescents (13–17 years) *n* = 60	Reduction of self-injury and ED symptoms
Kiani et al. (434)	A: ADHD, B: ED & executive functions	MMT	Female adolescents (13–15 years) *n* = 30	Improvement of ED symptoms and executive functions scores ES = “large”
Marco et al. (454)	A: ODD, B: ED	DBT	Female adolescents (12–18 years) *n* = 2	Reduction of impulsive behaviors, maladaptive ER strategies
Marrow et al. (474)	A: PTSD, B: ED	TAU & trauma training for staff (CG) vs. TAU & environmental modifications (trauma training for staff, trauma affect regulation) (EG)	Detained adolescents (11–19 years) *n* = 74	Significant reduction in depression, threatening of staff, use of physical restraints, seclusion rates in the intervention program
McCauley et al. (164)	A: suicidality & NSSI, B: ED	DBT	Adolescents (12–18 years) *n* = 173	Improvement of all outcomes: Decrease of NSSI, risk of lifetime suicide attempt and ED; ES (end of active treatment) = 0.34, ES (end of follow up) = 0.11
Popolo et al. (446)	A: Personality disorder, B: ED & Alexithymia	MIT-G	Adolescents & young adults (16–25 years) *n* = 17	Improvement of specific symptoms of personality disorder and of functioning. Reduction of ED symptoms; ES(different symptoms) = 0.14–1.17
Schuppert et al. (455)	A: BPD, B: ED	ERT vs. TAU	Adolescents (14–19 years) *n* = 43	ERT had no additional effect on symptoms of BPD (including ED). Only TAU (medication, psychotherapy, systemic therapy …) improved BPD-symptoms (including ED)
Sharma-Patel et al. (100)	A: PTSD, B: ED	Tf-CBT	Children & adolescents (4–17 years) *n* = 118	Decrease of PTSD symptoms (ED included)
Sloan et al. (456)	A: SAD, B: ED & Anxiety & Depression	ERIC	Adolescents & young adults (16–20 years) *n* = 79	Significant reduction of ED in 60%, significant decrease of depression and anxiety ratings in 50–60%; ES = −0.53
Suveg et al. (457)	A: Anxiety disorders, B: ED	CBT	Children & adolescents (7–15 years) *n* = 37	Significant reduction of anxiety symptoms, improvement of ED and coping strategies for only one emotion (“worry”); ES = 0.82
Thornback and Muller (91)	A: PTSD, B: ED	Tf-CBT	Children (7–12 years) *n* = 107, 44 at follow up	Significant reduction of PTSD symptoms, decrease of the use of maladaptive ER strategies. ED was the best predictor for improvements of PTSD symptoms; ES (pre treatment to 6 months follow up) = 0.36

*BT, Behavioral Treatment; CBT, Cognitive Behavioral Treatment; Tf-CBT, Trauma-focused Cognitive Behavioral Treatment; CDBT, Dialectic Behavioral Treatment; CDRS, Children's Depression Rating Scale Revised; ERIC, Emotion Regulation and Impulse Control Treatment; ERITA, Emotion Regulation Individual Therapy for Adolescents; ES, effect size; ERT, Emotion Regulation Training; ETAU, Enhanced Treatment as Usual; IGST, Individual/Group Supportive Therapy; MATCH, Modular Approach to Therapy for children with anxiety, Depression, Trauma or Conduct Problems; MDT, Mode Deactivation Therapy; MFWSB, More Fun with Sisters and Brothers; MIT-G, Metacognitive Interpersonal Therapy in Groups; MMT, Mindfulness Meditation Therapy; PEER, Preventing Emotional Eating Routines; TARGET, Trauma Affect Regulation, Guide for Education and Therapy; TAU, Treatment as Usual; Tf-CBT, Trauma-focused Cognitive Behavioral Treatment*.

Tables 1, 2 summarize evaluated therapies focusing on ED. The tables are organized depending on ED being the primary therapeutic target (Table 1) or, if the underlying disease was the primary, and treatment of ED the secondary target (Table 2). The order of the listed studies is alphabetical according to the name of the first author of the respective study.

##### Pharmacotherapy

ED leads to psychopathology and a number of externalizing and internalizing symptoms, including anxiety, mood dysregulation, impulsiveness, behavioral issues, and serious debilitating social problems. Two forms of stress related sequelae have to be considered: (i) acute emotional cues triggering experiential, behavioral, central and peripheral physiological systems (458), and (ii) chronic mental stress resulting from continuous burdens, such as maltreatment or familial discord, leading to personality modification, ED, and related problems.

To date, there is no substance available for a causal treatment of ED. Among the available pharmaceuticals, the following classes of medication have been shown to alleviating symptoms of ED:

- Antipsychotics - sedating and distancing from mental tension (459, 460)
- Antidepressants and mood stabilizing medication - alleviating anxiety, improving mood, and decreasing maniac symptoms (458, 461)
- Sedatives/anxiolytics - sedating and alleviating anxiety
- ADHD Medication (55, 462)
- Combined medication (463)
- Experiential therapies, such as oxytocin (464)

##### Other Therapies

fMRI neurofeedback produces promising lab results for improving ER by changing the bloodflow to the amygdala, and the interconnection between the amygdala and the prefrontal cortex in patients with BPD (429, 465), MDD (466), or PTSD (467). The disadvantages lie in the poor availability and the complexity of the technology. Relaxation and mindfulness based therapy have been shown to reduce chronic stress activation interacting with the impaired threat perception of ED (468). Biofeedback has been shown to produce relaxing, threat and stress reducing effects within a short training time, depending on the patients' hypnotic ability (469). There are, however, only a few uncontrolled observational studies and single case reports supporting this approach.

Early parenting programs are effective in preventing or changing unfavorable environmental conditions. These programs focus on sensitivity, acceptance, making aware and avoiding self-defeating strategies, such as harsh educational measures, and help in improving empathic understanding, clarity, fairness, decision making, and coming to a decision (22). There are, however, reports of failing parenting programs [e.g., (470)], possibly related to the timing of the intervention and the already established dysfunctions on both sides, the caregivers and the children.

## Discussion

ED influences most child and adolescent psychiatric disorders by interfering with cognitive processes that interact with reactive processes. In some disorders, such as ADHD, psychosis, or affective disorders, ED is an essential but often neglected part of the related pschopathology.

Several mechanisms lead to the development of ED, with reference to anatomical, functional, family, social and relational bases. This causes severe stress, misunderstandings, relationship-difficulties, maladaptive ER strategies such as self-injurious behavior, externalizing and internalizing responses, endangerment of self, self-perception and social relationships.

The relationship between ED and nosology according to ICD or DSM has not been clarified as yet. The further development of this topic raises more questions than answers:

- Is ED the lowest common denominator of psychological disorders?
- How specific is its predictive value for explaining psychological disorders? Or does it rather sum up to one single concept concerning all psychological disorders?
- Would it therefore be the “Grand Unifying Theory” of psychological disorders?

ED may possibly be a new concept, allowing psychological disorders to be defined by their essential nature instead of being defined by their phenotype and objectively measured symptoms. ER and emotion processing would be more meaningful than single well-defined symptoms. It would render the individual personality and the dynamics of social relationships and communication more understandable.

As part of the health care system, many hospitals and psychotherapists are required to provide ICD-10 or DSM-5 diagnoses. These symptom-based and descriptive nosologies are useful for improving the accuracy and consistency of clinical diagnostics and form the basis for effective treatments.

Other transdiagnostic approaches can be seen as a very good supplement to traditional categorical diagnoses using the DSM or ICD thesauri. The approach of systemic therapy, for example, sees psychological symptoms, disorders, and their change, embedded in interactive and narrative structures.

Another very significant transdiagnostic approach are the Research Domain Criteria (RDoC) (471). Fernandez et al. proposed conceptualizing ER as a new, sixth domain in the RDoC matrix (472).

It is argued that in order to understand causes and mechanisms of mental disorders, many clinical symptoms can be viewed as being caused by emotionally dysregulated processes. In this way, the underlying structure of the mental disorder can be captured better (at least complementarily) than by a pure ICD or DSM approach. In the area of interventions, treatments that target common features of multiple disorders should be developed on this basis. ED is to be considered as such a common feature.

ER and ED concepts are relatively new on the agenda, therefore, specific treatments are still under development – including child and adolescent psychotherapies, positive parenting initiatives, sedating and mood stabilizing medication. To date no causal pharmacologic treatment is available. Research in patients with ASD suggests that oxytocin might be a candidate for future treatment options. Available treatment tools, such as CBT, DBT or mindfulness training need to be explored and evaluated more extensively.

Our narrative review, although carefully prepared and elaborated, bears the limitations of subjective selection of references and setting of priorities. The explained search strategy is not sufficient to conduct a systematic review because considerable heterogeneity in the definitions used by researchers and the possible variants of the label are not taken into account. Moreover, the review was apparently not conducted following PRISMA guidelines, as usually requested and expected, and the search was not preregistered on an international database of systematic reviews such as PROSPERO. Due to the complexity of the association of ED with numerous mental disorders, a systematic literature review as well as work based on the PRISMA criteria was not feasible. On the other hand, this review covers a very broad spectrum of major psychiatric disorders in general, rather than a specific one.

In addition, our review includes a limited age range of children and adolescents between 2 and 17 years. Therefore, ED in infancy has not been covered in this review.

Our review sheds light on a central topic of individual and social functioning that to date has been more regarded in its dysfunctional aspects of single psychiatric disorders. The concept of ED will probably help in discovering basic approaches to the understanding, diagnosis and treatment of psychiatric disorders especially in children and adolescents but will probably also prove a key issue in adult psychiatry.

## Data Availability Statement

Publicly available datasets were analyzed in this study. We searched the literature databases ERIC, PsycARTICLES, PsycINFO and PSYNDEX on June 29th, 2020 for peer reviewed articles, published in English language in between January, 2000 and June, 2020, and related to children and adolescents (2–18 years).

## Author Contributions

FP: idea, conceptualization, structure of the work, preparations, literature analysis, methods section, several chapters, tables, the figure, corrections, and discussion. SO: several chapters, tables, and corrections. EM and PP: corrections and discussion. CP: several chapters, structure of the work, tables, literature, corrections, and discussion. All authors contributed to the article and approved the submitted version.

## Conflict of Interest

The authors declare that the research was conducted in the absence of any commercial or financial relationships that could be construed as a potential conflict of interest.

## Publisher's Note

All claims expressed in this article are solely those of the authors and do not necessarily represent those of their affiliated organizations, or those of the publisher, the editors and the reviewers. Any product that may be evaluated in this article, or claim that may be made by its manufacturer, is not guaranteed or endorsed by the publisher.

## Abbreviations

ACE: Adverse Childhood Experiences
ADHD: Attention Deficit/Hyperactivity Disorder
AN: Anorexia Nervosa
ASD: Autism Spectrum Disorder
BD: Bipolar Disorder
BN: Bulimia Nervosa
BPD: Borderline Personality Disorder
CBT: Cognitive Behavioral Therapy
CD: Conduct Disorder
CU: Callous-Unemotional (traits)
DBD: Disruptive Behavioral Disorder
DMDD: Disruptive Mood Dysregulation Disorder
EatD: Eating Disorder(s)
ED: Emotional Dysregulation
ER: Emotional Regulation
LGB: Lesbian, Gay and Bisexual
MPH: Methylphenidate
NSSI: Non-Suicidal Self-Injuring
OB: Obesity
ODD: Oppositional Defiant Disorder
PFC: Prefrontal Cortex
PTSD: Post Traumatic Stress Disorder
RSA: Respiratory Sinus Arrhythmia
SES: Socio Economic Status
SUD: Substance Use Disorder
TD: Typically Developing.

## References

[1] Thompson RA. Emotion regulation: a theme in search of definition. Monogr Soc Res Child Dev. (1994) 59:25–52. 10.1111/j.1540-5834.1994.tb01276.x 7984164

[2] Gratz KL Roemer L. Multidimensional assessment of emotion regulation and dysregulation: development, factor structure, and initial validation of the difficulties in emotion regulation scale. J Psychopathol Behav Assess. (2004) 26:41–54. 10.1023/B:JOBA.0000007455.08539.94

[3] Gross JJ Thompson RA. Emotion regulation. Conceptual foundations, In: Gross J, editor. Handbook of Emotion Regulation. New York, NY: Guilford Press (2007).

[4] Esbjørn BH Bender PK Reinholdt-Dunne ML Munck LA Ollendick TH. The development of anxiety disorders: Considering the contributions of attachment and emotion regulation. Clin Child Fam Psychol Rev. (2011) 15:129–43. 10.1007/s10567-011-0105-4 22116623

[5] Aldao A Nolen-Hoeksema S. When are adaptive strategies most predictive of psychopathology? J Abnorm Psychol. (2012) 121:276–81. 10.1037/a0023598 21553934

[6] Takahashi T Chanen AM Wood SJ Yücel M Tanino R Michio S. . Insular cortex volume and impulsivity in teenagers with first-presentation borderline personality disorder. Progress Neuro-Psychopharmacol Biol Psychiatry. (2009) 33:1395–400. 10.1016/j.pnpbp.2009.07.017 19632284

[7] Spencer AE Uchida M Kenworthy T Keary CJ Joseph B. Glutamatergic dysregulation in pediatric psychiatric disorders. J Clin Psychiatry. (2014) 75:1226–41. 10.4088/JCP.13r08767 25271988

[8] Schmahmann JD Weilburg JB Sherman JC. The Neuropsychiatry of the cerebellum - insights from the clinic. Cerebellum. (2007) 6:254–67. 10.1080/14734220701490995 17786822

[9] Bertocci MA Bebko G Olino T Fournier J Hinze AK. Behavioral and emotional dysregulation trajectories marked by prefrontal-amygdala function in symptomatic youth. Psychol Med. (2014) 44:2603–15. 10.1017/S0033291714000087 24468022 PMC4344801

[10] Spechler PA Chaarani B Orr C Mackey S Higgins ST Banaschewski T . Neuroimaging evidence for right orbitofrontal cortex differences in adolescents with emotional and behavioral dysregulation. J Am Acad Child Adolesc Psychiatry. (2019) 58:1092–103. 10.1016/j.jaac.2019.01.021 31004740

[11] van Roekel E Verhagen M Engels RCME Kuppens P. Variation in the serotonin transporter polymorphism (5-HTTLPR) and inertia of negative and positive emotions in daily life. Emotion. (2018) 18:229–36. 10.1037/emo0000336 28569537

[12] Gross JJ. Emotion regulation: taking stock and moving forward. Emotion. (2013) 13:359–65. 10.1037/a0032135 23527510

[13] Bretherton. I. The origins of attachment theory: John Bowlby, Mary Ainsworth. Dev Psychol. (1992) 28:759–75. 10.1037/0012-1649.28.5.759

[14] Hee Yoo S Matsumoto D LeRoux JA. The influence of emotion recognition and emotion regulation on intercultural adjustment. Int J Intercult Relations. (2006) 30:345–63. 10.1016/j.ijintrel.2005.08.006

[15] Guyer AE McClure EB Adler AD Brotman MA Rich BA Kimes S . Specificity of facial expression labeling deficits in childhood psychopathology. J Child Psychol Psychiatry. (2007) 48:863–71. 10.1111/j.1469-7610.2007.01758.x 17714371

[16] Pons F Lawson J Harris PL de Rosnay M. Individual differences in children's emotion understanding: Effects of age and language. Scand J Psychol. (2003) 44:347–53. 10.1111/1467-9450.00354 12887556

[17] Carlson SM Wang TS. Inhibitory control and emotion regulation in preschool children. Cogn Dev. (2007) 22:489–510. 10.1016/j.cogdev.2007.08.002

[18] Groves NB Kofler MJ Wells EL Day TN Chan ESM. An examination of relations among working memory, ADHD symptoms, emotion regulation. J Abnorm Child Psychol. (2020) 48:525–37. 10.1007/s10802-019-00612-8 31900835 PMC7318097

[19] Cole PM Michel MK O'Donnell Teti L. The development of emotion regulation and dysregulation: a clinical perspective. Monogr Soc Res Child Dev. (1994) 59:73–102. 10.1111/j.1540-5834.1994.tb01278.x 7984169

[20] Ross A Thompson RA. Emotion and emotion regulation: Two sides of the developing coin. Emotion Review. (2011) 3:53–61. 10.1177/1754073910380969

[21] Loevaas MES Sund AM Patras J Martinsen K Hjemdal O Neumer SP . Emotion regulation and its relation to symptoms of anxiety and depression in children aged 8-12 years: does parental gender play a differentiating role? BMC Psychol. (2018) 6:255. 10.1186/s40359-018-0255-y 30126444 PMC6102894

[22] Noroña AN Tung I Lee SS Blacher J Crnic KA Baker BL . Developmental patterns of child emotion dysregulation as predicted by serotonin transporter genotype and parenting. J Clin Child Adolesc Psychol. (2017) 47:S354–68. 10.1080/15374416.2017.1326120 28617048 PMC6324841

[23] Beauchaine TP Gatzke-Kopp L Mead HK. Polyvagal theory and developmental psychopathology: Emotional dysregulation and conduct problems from preschool to adolescence. Biol Psychol. (2007) 74:174–84. 10.1016/j.biopsycho.2005.08.008 17045726 PMC1801075

[24] Ambrosini PJ Bennett DS Elia J. Attention deficit hyperactivity disorder characteristics: II. Clinical correlates of irritable mood. J Affect Disord. (2013) 145:70–6. 10.1016/j.jad.2012.07.014 22868057 PMC3496809

[25] Tonacci A Billeci L Calderoni S Levantini V Masi G Milone A. Sympathetic arousal in children with oppositional defiant disorder and its relation to emotional dysregulation. J Affect Disord. (2019) 257:207–13. 10.1016/j.jad.2019.07.046 31301625

[26] Shaw P Stringaris A Nigg J Leibenluft E. Emotion dysregulation in attention deficit hyperactivity disorder. Am J Psychiatry. (2014) 171:276–93. 10.1176/appi.ajp.2013.13070966 24480998 PMC4282137

[27] Gratz KL Weiss NH McDermott MJ Dilillo D Messman-Moore T Tull MT. Emotion dysregulation mediates the relation between borderline personality disorder symptoms and later physical health symptoms. J Pers Disord. (2017) 31:433–48. 10.1521/pedi_2016_30_252 27322577 PMC5472518

[28] Pat-Horenczyk R Cohen S Ziv Y Achituv M Asulin-Peretz L Blanchard TR . Emotion regulation in mothers and young children faced with trauma. Infant Ment Health J. (2015) 36:337–48. 10.1002/imhj.21515 25941026

[29] Biederman J Petty CR Day H Goldin RL Spencer T Faraone SV . Severity of the aggression/anxiety-depression/attention child behavior checklist profile discriminates between different levels of deficits in emotional regulation in youth with attention-deficit hyperactivity disorder. J Dev Behav Pediatrics. (2012) 33:236–43. 10.1097/DBP.0b013e3182475267 22278125 PMC3319866

[30] Dugal C Godbout N Bélanger C Hébert M Goulet M. Cumulative childhood maltreatment and subsequent psychological violence in intimate relationships: The role of emotion dysregulation. Partner Abuse. (2018) 9:18–40. 10.1891/1946-6560.9.1.18

[31] American Psychiatric Association. Diagnostic and Statistical Manual of Mental Disorders (DSM-5®), Fifth Edition. Washington, DC: American Psychiatric Association (2013). 10.1176/appi.books.9780890425596

[32] WHO. ICD-11 (2019). Available online at: https://icd.who.int/browse11/l-m/en. (accessed October 25, 2020).

[33] Wozniak J Biederman J Kiely K Ablon S Faraone SV Mundy E . Mania-like symptoms suggestive of childhood-onset bipolar disorder in clinically referred children. J Am Acad Child Adolesc Psychiatry. (1995) 34:867–76. 10.1097/00004583-199507000-00010 7649957

[34] Vacher C Goujon A Romo L Purper-Ouakil D. Efficacy of psychosocial interventions for children with ADHD and emotion dysregulation: A systematic review. Psychiatry Res. (2020) 291:113151. 10.1016/j.psychres.2020.113151 32619822

[35] Bottelier MA Schrantee A Ferguson B Tamminga H Bouziane C Kooij JJS . Age-dependent effects of acute methylphenidate on amygdala reactivity in stimulant treatment-naive patients with attention deficit/hyperactivity disorder. Psychiatry Res. (2017) 269:36–42. 10.1016/j.pscychresns.2017.09.009 28938219

[36] McQuade JD Breaux RP. Are elevations in ADHD symptoms associated with physiological reactivity and emotion dysregulation in children? J Abnorm Child Psychol. (2016) 45:1091–103. 10.1007/s10802-016-0227-8 27838892

[37] Qian Y Chang W He X Yang L Liu L Ma Q . Emotional dysregulation of ADHD in childhood predicts poor early-adulthood outcomes: A prospective follow up study. Res Dev Disabil. (2016) 59:428–36. 10.1016/j.ridd.2016.09.022 27744214

[38] Bunford N Evans SW Langberg JM. Emotion dysregulation is associated with social impairment among young adolescents with ADHD. J Atten Disord. (2018) 22:66–82. 10.1177/1087054714527793 24681899

[39] Gamli IS Tahiroglu AY. Six months methylphenidate treatment improves emotion dysregulation in adolescents with attention deficit/hyperactivity disorder: A prospective study. Neuropsychiatr Dis Treat. (2018) 14:1329–37. 10.2147/NDT.S164807 29872300 PMC5973442

[40] Wheeler Maegden J Carlson CL. Social functioning and emotional regulation in the attention deficit hyperactivity disorder. J Clin Child Psychol. (2000) 29:30–42. 10.1207/S15374424jccp2901_4 10693030

[41] Huguet A Izaguirre Eguren J Miguel-Ruiz D Vall Vallés X Alda JA. Deficient emotional self-regulation in children with attention deficit hyperactivity disorder. J Dev Behav Pediatrics. (2019) 40:425–31. 10.1097/DBP.0000000000000682 31135603

[42] Rosen PJ Factor PI. Emotional impulsivity and emotional and behavioral difficulties among children with ADHD. J Atten Disord. (2015) 19:779–93. 10.1177/1087054712463064 23172248

[43] Sørensen L Plessen KJ Nicholas J Lundervold AJ. Is behavioral regulation in children with ADHD aggravated by comorbid anxiety disorder? J Atten Disord. (2010) 15:56–66. 10.1177/1087054709356931 20071639

[44] Özbaran B Kalyoncu T Köse S. Theory of mind and emotion regulation difficulties in children with ADHD. Psychiatry Res. (2018) 270:117–22. 10.1016/j.psychres.2018.09.034 30245374

[45] Romvig Overgaard K Oerbeck B Aase H Torgersen S Reichborn-Kjennerud T Zeiner P. Emotional lability in preschoolers with symptoms of ADHD. J Atten Disord. (2015) 22:787–95. 10.1177/1087054715576342 25804545

[46] Lugo-Candelas C Flegenheimer C McDermott Harvey JME. Emotional understanding, reactivity, and regulation in young children with ADHD symptoms. J Abnorm Child Psychol. (2016) 45:1297–310. 10.1007/s10802-016-0244-7 27957717

[47] Cremone A Lugo-Candelas CI Harvey EA McDermott JM Rebecca Spencer MC. Positive emotional attention bias in young children with symptoms of ADHD. Child Neuropsychol. (2018) 24:1137–45. 10.1080/09297049.2018.1426743 29347861 PMC6136424

[48] Tenenbaum RB Musser ED Morris S Ward AR Raiker JS Coles EK . Pelham. Response inhibition, response execution, and emotion regulation among children with attention-deficit/hyperactivity disorder. J Abnormal Child Psychol. (2018) 47:589–603. 10.1007/s10802-018-0466-y 30112596 PMC6377355

[49] López-Martín S Albert J Fernández-Jaén A Carretié L. Emotional response inhibition in children with attention-deficit/hyperactivity disorder: neural and behavioural data. Psychol Med. (2015) 45:2057–71. 10.1017/S0033291714003195 25708692

[50] Seymour KE Tang X Crocetti D Mostofsky SH Miller MI Rosch KS. Anomalous subcortical morphology in boys, but not girls, with ADHD compared to typically developing controls and correlates with emotion dysregulation. Psychiatry Res. (2017) 261:20–8. 10.1016/j.pscychresns.2017.01.002 28104573 PMC5335909

[51] Bunford N Evans SW Wymbs F. ADHD and emotion dysregulation among children and adolescents. Clin Child Fam Psychol Rev. (2015) 18:185–217. 10.1007/s10567-015-0187-5 26243645

[52] Lee CA Milich R Lorch EP Flory K Sarno Owens J Lamont AE. Forming first impressions of children: the role of attentiondeficit/hyperactivity disorder symptoms and emotion dysregulation. J Child Psychol Psychiatry. (2017) 59:556–64. 10.1111/jcpp.12835 29083026

[53] Williams KE Sciberras E. Sleep and self-regulation from birth to 7 years. J Dev Behav Pediatrics. (2016) 37:385–94. 10.1097/DBP.0000000000000281 26982247

[54] Kutlu A Akyol Ardic U Sabri Ercan E. Effect of methylphenidate on emotional dysregulation in children with attention-deficit/hyperactivity disorder and oppositional defiant disorder/conduct disorder. J Clin Psychopharmacol. (2017) 37:220–5. 10.1097/JCP.0000000000000668 28225747

[55] Winters DE Fukui S Leibenluft E Hulvershorn LA. Improvements in irritability with open-label methylphenidate treatment in youth with comorbid attention deficit/hyperactivity disorder and disruptive mood dysregulation disorder. J Child Adolesc Psychopharmacol. (2018) 28:298–305. 10.1089/cap.2017.0124 29708762 PMC6016730

[56] Walter H von Kalckreuth A Schardt D Stephan A Goschke T Erk S. The temporal dynamics of voluntary emotion regulation. PLoS ONE. (2009) 4:e6726. 10.1371/journal.pone.0006726 21949675 PMC3175755

[57] Young KD Siegle GJ Misaki M Zotev V Phillips R Drevets WC . Altered task-based and resting-state amygdala functional connectivity following real-time fMRI amygdala neurofeedback training in major depressive disorder. NeuroImage: Clin. (2018) 17:691–703. 10.1016/j.nicl.2017.12.004 29270356 PMC5734798

[58] Folk JB Zeman JL Poon JA Dallaire DH. A longitudinal examination of emotion regulation: pathways to anxiety and depressive symptoms in urban minority youth. Child Adolesc Ment Health. (2014) 19:243–50. 10.1111/camh.12058 32878352

[59] Tahmouresi N Bender C Schmitz J Baleshzar A Tuschen-Caffier B. Similarities and differences in emotion regulation and psychopathology in Iranian and German school-children: A cross-cultural study. Int J Prev Med. (2014) 5:52–60. 24554992 PMC3915473

[60] Feng X Keenan K Hipwell AE Henneberger AK Rischall MS Butch J . Longitudinal associations between emotion regulation and depression in preadolescent girls: Moderation by the caregiving environment. Dev Psychol. (2009) 45:798–808. 10.1037/a0014617 19413432 PMC2679182

[61] Keenan K Hipwell AE. Preadolescent clues to understanding depression in girls. Clin Child Fam Psychol Rev. (2005) 8:89–105. 10.1007/s10567-005-4750-3 15984082 PMC2848388

[62] Mirsu-Paun A. Grief cognitions and cognitive-emotional regulation associated with romantic breakup distress among college students. Eur Psychiatry. (2016) 33:S284. 10.1016/j.eurpsy.2016.01.762

[63] Stegge H Meerum Terwogt M. Awareness and regulationof emotion in typical and atypical development. In: Gross JJ, editor. Handbook of Emotion Regulation. New York, NY: Guilford Press (2007).

[64] Fussner LM Luebbe AM Mancini KJ Becker SP. Emotion dysregulation mediates the longitudinal relation between peer rejection and depression. Int J Behav Dev. (2016) 42:155–66. 10.1177/0165025416669062

[65] Fulford D Eisner LR Johnson SL. Differentiating risk for mania and borderline personality disorder: The nature of goal regulation and impulsivity. Psychiatry Res. (2015) 227:347–52. 10.1016/j.psychres.2015.02.001 25892256

[66] Kafantaris V Kingsley P Ardekani B Saito E Lencz T Lim K . Lower orbital frontal white matter integrity in adolescents with bipolar I disorder. J Am Acade Child Adolesc Psychiatry. (2009) 48:79–86. 10.1097/CHI.0b013e3181900421 19050654 PMC2747245

[67] Chang KD Wagner C Garrett A Howe M Reis A. A preliminary functional magnetic resonance imaging study of prefrontal-amygdalaractivation changes in adolescents with bipolar depression treated with lamotrigine. Bipolar Disord. (2008) 10:426–31. 10.1111/j.1399-5618.2007.00576.x 18402630

[68] Roberts G Lord A Frankland A Wright A Lau P Levy F . Functional dysconnection of the inferior frontal gyrus in young people with bipolar disorder or at genetic high risk. Biol Psychiatry. (2017) 81:718–27. 10.1016/j.biopsych.2016.08.018 28031150

[69] Legenbauer T Heiler S Holtmann M Fricke-Oerkermann L Lehmkuhl G. The affective storms of school children during night time: Do affective dysregulated school children show a specific pattern of sleep disturbances? J Neural Transm. (2012) 119:989–98. 10.1007/s00702-012-0837-4 22684420

[70] Mehl RC O'Brien LM Jones JH Dreisbach JK Mervis CB Gozal D. Correlates of sleep and pediatric bipolar disorder. Sleep. (2006) 29:193–7. 10.1093/sleep/29.2.193 16494087

[71] Barch DM Harms MP Tillman R Hawkey E Luby JL. Early childhood depression, emotion regulation, episodic memory, hippocampal development. J Abnorm Psychol. (2019) 128:81–95. 10.1037/abn0000392 30628810 PMC6338439

[72] Kim P Arizpe J Rosen B Razdan V Catherine Haring C Sarah Jenkins S . Impaired fixation to eyes during facial emotion labelling in children with bipolar disorder or severe mood dysregulation. J Psychiatry Neurosci. (2013) 38:407–16. 10.1503/jpn.120232 23906351 PMC3819155

[73] Deveney CM Connolly ME Haring C Bones BL. Neural mechanisms of frustration in chronically irritable children. Am J Psychiatry. (2013) 170:1186–94. 10.1176/appi.ajp.2013.12070917 23732841 PMC3938281

[74] Melissa Brotman AMA Layla Kassem L Michelle Reising MMM Amanda Guyer EAE Daniel Dickstein PDP Brendan A . Parental diagnoses in youth with narrow phenotype bipolar disorder or severe mood dysregulation. Am J Psychiatry. (2007) 164:1208–41. 10.1176/appi.ajp.2007.06101619 17671287

[75] Schenkel LS Pavuluri MN Herbener ES Sweeney EM. Facial emotion processing in acutely ill and euthymic patients with pediatric bipolar disorder. J Am Acad Child Adolesc Psychiatry. (2007) 46:1070–9. 10.1097/chi.0b013e3180600fd6 17667485

[76] Perugi G Hantouche E Vannucchi G. Diagnosis and treatment of cyclothymia: the “primacy” of temperament. Curr Neuropharmacol. (2017) 15:372–9. 10.2174/1570159X14666160616120157 28503108 PMC5405616

[77] Kochman FJ Hantouche EG Ferrari P Lancrenon S Bayart D Akiskal HS. Cyclothymic temperament as a prospective predictor of bipolarity and suicidality in children and adolescents with major depressive disorder. J Affect Disord. (2005) 85:181–9. 10.1016/j.jad.2003.09.009 15780688

[78] Akiskal HS. Developmental pathways to bipolarity: are juvenile-onset depressions pre-bipolar? J Am Acad Child Adolesc Psychiatry. (1995) 34:754–63. 10.1097/00004583-199506000-00016 7608049

[79] Signoretta S Maremmani I Liguori A Perugi G Akiskal HS. Affective temperament traits measured by TEMPS-I and emotional-behavioral problems in clinically-well children, adolescents, young adults. J Affect Disord. (2005) 85:169–80. 10.1016/S0165-0327(03)00100-9 15780687

[80] Linehan M. Cognitive-Behavioral Treatment for Borderline Personality Disorder. New York, NY: Guilford Press (1993).

[81] Crowell SE Beauchaine TP McCauley E Smith CJ Stevens AL Sylvers P. Psychological, autonomic, and serotonergic correlates of parasuicide among adolescent girls. Dev Psychopathol. (2005) 17:1105–27. 10.1017/S0954579405050522 16613433

[82] Yen S Weinstock LM Andover MS Sheets ES Selby EA Spirito A. Prospective predictors of adolescent suicidality: 6-month post-hospitalization follow-up. Psychol Med. (2012) 43:983–93. 10.1017/S0033291712001912 22932393 PMC3663078

[83] Bowen R Rahman H Yue Dong L Khalaj S Baetz M Peters E . Suicidality in people with obsessive-compulsive symptoms or personality traits. Front Psychiatry. (2019) 9:747. 10.3389/fpsyt.2018.00747 30692943 PMC6339952

[84] Weinberg A Klonsky ED. Measurement of emotion dysregulation in adolescents. Psychol Assess. (2009) 21:616–21. 10.1037/a0016669 19947794

[85] Cloitre M Stolbach BC Herman JL van der Kolk B Pynoos R. A developmental approach to complex PTSD: Childhood and adult cumulative trauma as predictors of symptom complexity. J Trauma Stress. (2009) 22:399–408. 10.1002/jts.20444 19795402

[86] Young Choi J Ja Oh K. Cumulative childhood trauma and psychological maladjustment of sexually abused children in Korea: Mediating effects of emotion regulation. Child Abuse and Neglect. (2014) 38:296–303. 10.1016/j.chiabu.2013.09.009 24210271

[87] Dvir Y Ford JD Hill M Frazier JA. Childhood maltreatment, emotional dysregulation, psychiatric comorbidities. Harv Rev Psychiatry. (2014) 22:149–61. 10.1097/HRP.0000000000000014 24704784 PMC4091823

[88] van der Kolk BA. Developmental trauma disorder: Toward a rational diagnosis for children with complex trauma histories. Psychiatr Ann. (2005) 35:401–8. 10.3928/00485713-20050501-06

[89] McLaughlin KA Hatzenbuehler ML Mennin DS Nolen-Hoeksema S. Emotion dysregulation and adolescent psychopathology: A prospective study. Behav Res Ther. (2011) 49:544–54. 10.1016/j.brat.2011.06.003 21718967 PMC3153591

[90] Rizeq J McCann D. Trauma and affective forecasting: The mediating effect of emotion dysregulation on predictions of negative affect. Pers Individ Dif. (2019) 147:172–6. 10.1016/j.paid.2019.04.036

[91] Thornback K Muller RT. Relationships among emotion regulation and symptoms during trauma-focused CBT for school-aged children. Child Abuse and Neglect. (2015) 50:182–92. 10.1016/j.chiabu.2015.09.011 26470906

[92] Spinazozola J van der Kolk B Ford JD. When nowhere is safe: interpersonal trauma and attachment adversity as antecedents of posttraumatic stress disorder and developmental trauma disorder. J Trauma Stress. (2018) 31:631–42. 10.1002/jts.22320 30338544 PMC6221128

[93] Ford JD Spinazzola J van der Kolk B Grasso D. Toward an empirically-based Developmental Trauma Disorder diagnosis for children: Factor structure, item characteristics, reliability and validity of the Developmental Trauma Disorder Semi-Structured Interview (DTD-SI). J Clin Psychiatry. (2018) 79:e1–9. 10.4088/JCP.17m11675 30256549

[94] Powers A Stevens JS O'Banion D Stenson AF Kaslow N Jovanovic T . Intergenerational transmission of risk for PTSD symptoms in African American children: The roles of maternal and child emotion dysregulation. Psychol Trauma. (2020). 10.1037/tra0000543 PMC7329591 31894989

[95] Kim J Cicchetti D. Longitudinal pathways linking child maltreatment, emotion regulation, peer relations, and psychopathology. J Child Psychol Psychiatry. (2009) 51:706–16. 10.1111/j.1469-7610.2009.02202.x 20050965 PMC3397665

[96] Larsen RJ Diener E. Affect intensity as an individual difference characteristic: A review. J Res Pers. (1987) 21:1–39. 10.1016/0092-6566(87)90023-7

[97] Conley RR Ascher-Svanum H Zhu B Faries D Kinon BJ. The burden of depressive symptoms in the long-term treatment of patients with schizophrenia. Schizophr Res. (2007) 90:186–97. 10.1016/j.schres.2006.09.027 17110087 PMC1937504

[98] Samson AC Huber O Gross JJ. Emotion regulation in Asperger's syndrome and high-functioning autism. Emotion. (2012) 12:659–65. 10.1037/a0027975 22642342

[99] Shields A Cicchetti D. Parental maltreatment and emotion dysregulation as risk factors for bullying and victimization in middle childhood. J Clin Child Psychol. (2001) 30:349–63. 10.1207/S15374424JCCP3003_7 11501252

[100] Sharma-Patel K Brown EJ. Emotion regulation and self blame as mediators and moderators of trauma-specific treatment. Psychol Violence. (2016) 6:400–9. 10.1037/vio0000044

[101] Lehmann S Breivik K Monette S Minnis H. Potentially traumatic events in foster youth, and association with DSM-5 trauma- and stressor related symptoms. Child Abuse Neglect. (2020) 101:104374. 10.1016/j.chiabu.2020.104374 31982843

[102] Langeland W Dijkstra S. Breaking the intergenerational transmission of child abuse: Beyond the mother-child relationship. Child Abuse Review. (1995) 4:4–13. 10.1002/car.2380040104

[103] Kessler RC Petukhova M Sampson NA Zaslavsky AM Wittchen HU. Twelve-month and lifetime prevalence and lifetime morbid risk of anxiety and mood disorders in the United States. Int J Methods Psychiatr Res. (2012) 21:169–84. 10.1002/mpr.1359 22865617 PMC4005415

[104] Greif Green J McLaughlin KA Berglund PA Gruber MJ Sampson NA Zaslavsky AM . Childhood adversities and adult psychiatric disorders in the National Comorbidity Survey Replication I. Arch Gen Psychiatry. (2010) 67:113–23. 10.1001/archgenpsychiatry.2009.186 20124111 PMC2822662

[105] Nusslock R Miller GE. Early-life adversity and physical and emotional health across the lifespan: A neuroimmune network hypothesis. Biol Psychiatry. (2016) 80:23–32. 10.1016/j.biopsych.2015.05.017 26166230 PMC4670279

[106] Smith AL Cross D Winkler J Jovanovic T Bradley B. Emotional dysregulation and negative affect mediate the relationship between maternal history of child maltreatment and maternal child abuse potential. J Fam Violence. (2014) 29:483–94. 10.1007/s10896-014-9606-5

[107] Warmingham JM Handley ED Rogosch FA Manly JT Cicchetti D. Identifying maltreatment subgroups with patterns of maltreatment subtype and chronicity: A latent class analysis approach. Child Abuse Neglect. (2019) 87:28–39. 10.1016/j.chiabu.2018.08.013 30224068 PMC6348036

[108] Briere J. Treating adults severely abused as children: The self-trauma model. In: Wolfe DA McMahon B Peters RD, editors. Child Abuse: New Directions in Treatment and Prevention Across the Lifespan. Newbury Park, CA: Sage Publications (1997).

[109] Briere J. Treating adult survivors of severe childhood abuse and neglect: Further development of an integrative model. In: Myers JEB Berliner L Briere J Reid T Jenny C, editors. The APSAC Handbook on Child Maltreatment. Newbury Park, CA: Sage Publications (2002).

[110] Freyd JJ. Betrayal Trauma: The Logic of Forgetting Childhood Abuse. Cambridge: Havard University Press (1996).

[111] Sevecke K Franke S Kosson D Krischer M. Emotional dysregulation and trauma predicting psychopathy dimensions in female and male juvenile offenders. Child Adolesc Psychiatry Ment Health. (2016) 10:130. 10.1186/s13034-016-0130-7 27822303 PMC5088678

[112] Herts KL McLaughlin KA Hatzenbuehler ML. Emotion dysregulation as a mechanism linking stress exposure to adolescent aggressive behavior. J Abnorm Child Psychol. (2012) 40:1111–22. 10.1007/s10802-012-9629-4 22466516 PMC3448707

[113] McLaughlin KA Hatzenbuehler ML Hilt LM. Emotion dysregulation as a mechanism linking peer victimization to internalizing symptoms in adolescents. J Consult Clin Psychol. (2009) 77:894–904. 10.1037/a0015760 19803569 PMC2778003

[114] Vitaro F Brendgen M Trembley RE. Reactively and proactively aggressive children: antecedent and subsequent characteristics. J Child Psychol Psychiatry. (2002) 43:495–506. 10.1111/1469-7610.00040 12030595

[115] Jacoby VM Krackow E Scotti JR. Betrayal trauma in youth and negative communication during a stressful task. Int J Aging Human Dev. (2016) 84:247–75. 10.1177/0091415016669724 28195013

[116] Maughan A Cicchetti D. Impact of child maltreatment and interadult violence on children's emotion regulation abilities and socioemotional adjustment. Child Dev. (2002) 73:1525–42. 10.1111/1467-8624.00488 12361317

[117] Nederlof E Van der Ham JM Dingemans PMJA Oei TI. The relation between dimensions of normal and pathological personality and childhood maltreatment in incarcerated boys. J Pers Disord. (2010) 24:746–62. 10.1521/pedi.2010.24.6.746 21158597

[118] Meredith Gruhn A MA Compas BE. Effects of maltreatment on coping and emotion regulation in childhood and adolescence: A meta-analytic review. Child Abuse Neglect. (2020) 103:104446. 10.1016/j.chiabu.2020.104446 32200195 PMC12352122

[119] Vettese LC Dyer CE Ling Li W Wekerle C. Does self-compassion mitigate the association between childhood maltreatment and later emotion regulation difficulties? A preliminary investigation. Int J Ment Health Addict. (2011) 9:480–91. 10.1007/s11469-011-9340-7

[120] Krain Roy A Lopes V Klein RG. Disruptive mood dysregulation disorder: A new diagnostic approach to chronic irritability in youth. Am J Psychiatry. (2014) 171:918–24. 10.1176/appi.ajp.2014.13101301 25178749 PMC4390118

[121] Heleniak C King KM Monahan KC McLaughlin KA. Disruptions in emotion regulation as a mechanism linking community violence exposure to adolescent internalizing problems. J Res Adolesc. (2017) 28:229–44. 10.1111/jora.12328 28646545 PMC5742089

[122] Miller DJ Vachon DD Aalsma MC. Negative affect and emotion dysregulation. Crim Justice Behav. (2012) 39:1316–27. 10.1177/0093854812448784

[123] Schelble JL Franks BA Miller MD. Emotion dysregulation and academic resilience in maltreated children. Child Youth Care Forum. (2010) 39:289–303. 10.1007/s10566-010-9105-7

[124] Bielas H Barra S Skrivanek C Aebi M Steinhausen HC. The associations of cumulative adverse childhood experiences and irritability with mental disorders in detained male adolescent offenders. Child Adolesc Psychiatry Ment Health. (2016) 10:2016. 10.1186/s13034-016-0122-7 27688799 PMC5034668

[125] Heleniak C Jenness JL Vander Stoep A McCauley E McLaughlin KA. Childhood maltreatment exposure and disruptions in emotion regulation: A transdiagnostic pathway to adolescent internalizing and externalizing psychopathology. Cognit Ther Res. (2015) 40:394–415. 10.1007/s10608-015-9735-z 27695145 PMC5042349

[126] Sachs-Ericsson NJ Sheffler JL Stanley IH Piazza JR Preacher KJ. When emotional pain becomes physical: Adverse childhood experiences, pain, and the role of mood and anxiety disorders. J Clin Psychol. (2017) 73:1403–28. 10.1002/jclp.22444 28328011 PMC6098699

[127] Xu Peh C Shahwan S Fauziana R Mahesh MV Sambasivam R Zhang Y. Emotion dysregulation as a mechanism linking child maltreatment exposure and self-harm behaviors in adolescents. Child Abuse Neglect. (2017) 67:383–90. 10.1016/j.chiabu.2017.03.013 28371647

[128] Berman AK Knight RA. The relation of familiarity with sexual abusers to subsequent developmental adaptation in youths who have sexually offended. Sexual Abuse. (2014) 27:587–608. 10.1177/1079063214544329 25053106

[129] Kroupina MG Fuglestad AJ Iverson SL Mason JH. Adoption as an intervention for institutionally reared children: HPA functioning and developmental status. Infant Behav Dev. (2012) 35:829–37. 10.1016/j.infbeh.2012.07.011 22986178

[130] Arria A O'Grady K Caldeira K Vincent K Wilcox H Wish E. Suicide ideation among college students: A multivariate analysis. Arch Suicide Res. (2009) 13:230–46. 10.1080/13811110903044351 19590997 PMC2709750

[131] Freyd JJ. Betrayal trauma: Traumatic amnesia as an adaptive response to childhood abuse. Ethics Behav. (1994) 4:307–29. 10.1207/s15327019eb0404_1

[132] Schwartz D Proctor LJ. Community violence exposure and children's social adjustment in the school peer group: The mediating roles of emotion regulation and social cognition. J Consult Clin Psychol. (2000) 68:670–83. 10.1037/0022-006X.68.4.670 10965642

[133] Langer Zarling A Taber-Thomas S Murray A Knuston JF Lawrence E Valles NL . Internalizing and externalizing symptoms in young children exposed to intimate partner violence: Examining intervening processes. J Family Psychol. (2013) 27:945–55. 10.1037/a0034804 24294933 PMC5308783

[134] Kaszynski K Kallis DL Karnik N Soller M Hunter S Haapanen R . Incarcerated youth with personality disorders: Prevalence, comorbidity and convergent validity. Personal Ment Health. (2014) 8:42–51. 10.1002/pmh.1241 24532554

[135] Trent ES Viana AG Raines EM Woodward EC Zvolensky MJ Candelari AE. Exposure to parental threatening behaviors and internalizing psychopathology in a trauma-exposed inpatient adolescent sample. J Nervous Mental Dis. (2019) 207:969–76. 10.1097/NMD.0000000000001058 31503185

[136] Hatzis D Dawe S Harnett P Loxton N. An investigation of the impact of childhood trauma on quality of caregiving in high risk mothers: Does maternal substance misuse confer additional risk? Child Psychiatry Hum Dev. (2019) 50:835–45. 10.1007/s10578-019-00886-5 30929117

[137] Ghorbani F Khosravani V Mohammadzadeh A Shadnia S. The role of emotion dysregulation in the relation of childhood trauma to heroin craving in individuals with heroin dependence. Drug Alcohol Depend. (2019) 195:132–9. 10.1016/j.drugalcdep.2018.12.008 30634108

[138] Mohammadzadeh A Ganji Z Khosravani V Mohammadpanah Ardakan A Amirinezhad A. Direct and indirect associations between perception of childhood trauma and suicidal ideation through emotion dysregulation in males who use heroin. Addict Behav. (2019) 98:106011. 10.1016/j.addbeh.2019.05.035 31233952

[139] Shipman K Zeman J Penza S Champion K. Emotion management skills in sexually maltreated and nonmaltreated girls: A developmental psychopathology perspective. Dev Psychopathol. (2000) 12:47–62. 10.1017/S0954579400001036 10774595

[140] Shenk CE Noll JG Cassarly JA. A multiple mediational test of the relationship between childhood maltreatment and non-suicidal self-injury. J Youth Adolesc. (2009) 39:335–42. 10.1007/s10964-009-9456-2 19798560 PMC2977983

[141] Chaplo SD Kerig KP Bennett DC Modrowski CA. The roles of emotion dysregulation and dissociation in the association between sexual abuse and self-injury among juvenile justice-involved youth. J Trauma Dissociation. (2015) 16:272–85. 10.1080/15299732.2015.989647 25759937

[142] Bierman KL Kalvin CB Heinrichs BS. Early childhood precursors and adolescent sequelae of grade school peer rejection and victimization. J Clin Child Adoles Psychol. (2014) 44:367–79. 10.1080/15374416.2013.873983 24527989 PMC4133343

[143] Hébert M Langevin R Charest F. Disorganized attachment and emotion dysregulation as mediators of the association between sexual abuse and dissociation in preschoolers. J Affect Disord. (2020) 267:220–8. 10.1016/j.jad.2020.02.032 32217222

[144] Josephine Tejada A Linder SM. The influence of child sexual abuse on preschool-aged children. Early Child Dev Care. (2018) 190:1833–43. 10.1080/03004430.2018.1542384

[145] Girard M Dugal C Hébert M Godbout N. Is my sex life ok? The mediating role of sexual anxiety in the association between childhood sexual abuse and sexual coercion against women. J Child Sex Abus. (2020) 29:717–33. 10.1080/10538712.2020.1774697 32529921

[146] Villalta L Khadr S Chua KC Kramer T Clarke V. Complex post-traumatic stress symptoms in female adolescents: the role of emotion dysregulation in impairment and trauma exposure after an acute sexual assault. Eur J Psychotraumatol. (2020) 11:1710400. 10.1080/20008198.2019.1710400 32002143 PMC6968575

[147] Greenbaum VJ. commercial sexual exploitation and sex trafficking of children in the United States. Curr Probl Pediatr Adolesc Health Care. (2014) 44:245–69. 10.1016/j.cppeds.2014.07.001 25131563

[148] Hopper EK. Polyvictimization and developmental trauma adaptations in sex trafficked youth. J Child Adolesc Trauma. (2016) 10:161–73. 10.1007/s40653-016-0114-z

[149] Ahmed SP Bittencourt-Hewitt A Sebastian CL. Neurocognitive bases of emotion regulation development in adolescence. Dev Cogn Neurosci. (2015) 15:11–25. 10.1016/j.dcn.2015.07.006 26340451 PMC6989808

[150] Malter Cohen M Jing D Yang RR Tottenham N Lee FS Casey BJ. Early-life stress has persistent effects on amygdala function and development in mice and humans. Proc Nat Acad Sci USA. (2013) 110:18274–8. 10.1073/pnas.1310163110 24145410 PMC3831447

[151] Fishbein D Warner T Krebs C Trevarthen N Flannery B Hammond J. Differential relationships between personal and community stressors and children's neurocognitive functioning. Child Maltreat. (2008) 14:299–315. 10.1177/1077559508326355 18971345 PMC10506695

[152] Cicchetti D Rogosch FA Gunnar MR Toth SL. The differential impacts of early physical and sexual abuse and internalizing problems on daytime cortisol rhythm in school-aged children. Child Dev. (2010) 81:252–69. 10.1111/j.1467-8624.2009.01393.x 20331666 PMC2846099

[153] Rees CA Selwyn J. Non-infant adoption from care: lessons for safeguarding children. Child Care Health Dev. (2009) 35:561–7. 10.1111/j.1365-2214.2009.00978.x 19638026

[154] Panlilio CC Harden BJ Harring J. School readiness of maltreated preschoolers and later school achievement: The role of emotion regulation, language, and context. Child Abuse Neglect. (2018) 75:82–91. 10.1016/j.chiabu.2017.06.004 28601356

[155] Glaser D. Child abuse and neglect and the brain-a review. J Child Psychol Psychiatry. (2000) 41:97–116. 10.1111/1469-7610.00551 10763678

[156] Rutter ML. Psychosocial adversity and child psychopathology. Br J Psychiatry. (1999) 174:480–93. 10.1192/bjp.174.6.480 10616625

[157] Horowitz MJ. Stress Response Syndromes. 5th edition. Northvale, NJ: Jason Aronson (2011).

[158] Lanius RA Vermetten E Loewenstein RJ Brand B Schmahl C Douglas Bremner J . Emotion modulation in PTSD: Clinical and neurobiological evidence for a dissociative subtype. Am J Psychiatry. (2010) 167:640–7. 10.1176/appi.ajp.2009.09081168 20360318 PMC3226703

[159] Tull MT Barrett HM McMillan ES Roemer L. A preliminary investigation of the relationship between emotion regulation difficulties and posttraumatic stress symptoms. Behav Ther. (2007) 38:303–13. 10.1016/j.beth.2006.10.001 17697854

[160] Seligowski AV Lee DJ Bardeen JR Orcutt HK. Emotion regulation and posttraumatic stress symptoms: A meta-analysis. Cogn Behav Ther. (2014) 44:87–102. 10.1080/16506073.2014.980753 25421727

[161] Kaczkurkin AN Zang Y Gay NG Peterson AL Yarvis JS Borah EV . Cognitive emotion regulation strategies associated with the DSM-5 posttraumatic stress disorder criteria. J Trauma Stress. (2007) 30:343–50. 10.1002/jts.22202 28665526

[162] Weissman DG Bitran D Bryant Miller A Schaefer JD Sheridan MA McLaughlin . Difficulties with emotion regulation as a transdiagnostic mechanism linking child maltreatment with the emergence of psychopathology. Dev Psychopathol. (2019) 31:899–915. 10.1017/S0954579419000348 30957738 PMC6620140

[163] Wolf RC Herringa RJ. Prefrontal-amygdala dysregulation to threat in pediatric posttraumatic stress disorder. Neuropsychopharmacology. (2015) 41:822–31. 10.1038/npp.2015.209 26171717 PMC4707828

[164] McCauley E Berk MS Asarnow JR Adrian M Cohen J Korslund K . Efficacy of dialectical behavior therapy for adolescents at high risk for suicide. JAMA Psychiatry. (2018) 75:777. 10.1001/jamapsychiatry.2018.1109 29926087 PMC6584278

[165] Briggs-Gowan MJ Carter AS Clark R Augustyn M McCarthy KJ Ford JD. Exposure to potentially traumatic events in early childhood: differential links to emergent psychopathology. J Child Psychol Psychiatry. (2010) 51:1132–40. 10.1111/j.1469-7610.2010.02256.x 20840502 PMC3106304

[166] Grasso DJ Ford JD Briggs-Gowan MJ. Early life trauma exposure and stress sensitivity in young children. J Pediatr Psychol. (2013) 38:94–103. 10.1093/jpepsy/jss101 23008502 PMC3547236

[167] Scheeringa MS Zeanah CH Myers L Putnam FP. New findings on alternative criteria for PTSD in preschool children. J Am Acad Child Adolesc Psychiatry. (2003) 42:561–70. 10.1097/01.CHI.0000046822.95464.14 12707560

[168] Mongillo EA Briggs-Gowan M Ford J Carter AS. Impact of traumatic life events in a community sample of toddlers. J Abnorm Child Psychol. (2008) 37:455–68. 10.1007/s10802-008-9283-z 19034643

[169] Viana AG Woodward EC Raines EM Hanna AE Zvolensky MJ. The role of emotional clarity and distress tolerance in deliberate self-harm in a sample of trauma-exposed inpatient adolescents at risk for suicide. Gen Hosp Psychiatry. (2018) 50:119–24. 10.1016/j.genhosppsych.2017.10.009 29161582

[170] Viana AG Raines EM Woodward EC Hanna AE Walker R Zvolensky MJ. The relationship between emotional clarity and suicidal ideation among trauma-exposed adolescents in inpatient psychiatric care: does distress tolerance matter? Cogn Behav Ther. (2018) 48:430–44. 10.1080/16506073.2018.1536163 30457457

[171] van der Kolk BA Roth S Pelcovitz D Sunday S Spinazzola J. Disorders of extreme stress: The empirical foundation of a complex adaptation to trauma. J Trauma Stress. (2005) 18:389–99. 10.1002/jts.20047 16281237

[172] Buckholdt KE Weiss NH Young J Gratz KL. Exposure to violence, posttraumatic stress symptoms, and borderline personality pathology among adolescents in residential psychiatric treatment: The influence of emotion dysregulation. Child Psychiatry Hum Dev. (2014) 46:884–92. 10.1007/s10578-014-0528-5 25500759 PMC4466212

[173] Espil FM Viana AG Dixon LJ. Post-traumatic stress disorder and depressive symptoms among inpatient adolescents: The underlying role of emotion regulation. Residential Treatment Children Youth. (2016) 33:51–68. 10.1080/0886571X.2016.1159939

[174] Marsee MA. Reactive aggression and posttraumatic stress in adolescents affected by Hurricane Katrina. J Clin Child Adolesc Psychol. (2008) 37:519–29. 10.1080/15374410802148152 18645743

[175] Bennett DC Modrowski CA Chaplo SD Kerig PK. Facets of emotion dysregulation as mediators of the association between trauma exposure and posttraumatic stress symptoms in justice-involved adolescents. Traumatology. (2016) 22:174–83. 10.1037/trm0000085

[176] Miller MA Marsee MA. Emotional reactivity and antisocial behavior relative to posttraumatic stress symptom expression: a latent profile analysis. J Abnorm Child Psychol. (2019) 47:1339–50. 10.1007/s10802-019-00514-9 30729378

[177] Reich WA. Mental health screening outcomes among justice-involved youths under community supervision. J Offender Rehabil. (2014) 53:211–30. 10.1080/10509674.2014.887607

[178] Kelley LP Weathers FW McDevitt-Murphy ME Eakin DE Flood AM. A comparison of PTSD symptom patterns in three types of civilian trauma. J Trauma Stress. (2009) 22:227–35. 10.1002/jts.20406 19444884

[179] Ford JD Gagnon K Connor DF Pearson G. History of interpersonal violence, abuse, and nonvictimization trauma and severity of psychiatric symptoms among children in outpatient psychiatric treatment. J Interpers Violence. (2011) 26:3316–37. 10.1177/0886260510393009 21362676

[180] Naomi Breslau N. The epidemiology of trauma, PTSD, and other posttrauma disorders. Trauma Violence Abuse. (2009) 10:198–210. 10.1177/1524838009334448 19406860

[181] Salazar AM Keller TE Gowen LK Courtney ME. Trauma exposure and PTSD among older adolescents in foster care. Soc Psychiatry Psychiatr Epidemiol. (2012) 48:545–51. 10.1007/s00127-012-0563-0 22898825 PMC4114143

[182] Valdez CE Bailey BE Santuzzi AM Lilly MM. Trajectories of depressive symptoms in foster youth transitioning into adulthood. Child Maltreat. (2014) 19:209–18. 10.1177/1077559514551945 25248919

[183] Kelly NR Tanofsky-Kraff M Vannucci A Ranzenhofer LM Altschul AM Natasha Schvey ANA . Emotion dysregulation and loss-of-control eating in children and adolescents. Health Psychol. (2016) 35:1110–9. 10.1037/hea0000389 27505194 PMC5033667

[184] Weiss NH Tull MT Lavender J Gratz KL. Role of emotion dysregulation in the relationship between childhood abuse and probable PTSD in a sample of substance abusers. Child Abuse Neglect. (2013) 37:944–54. 10.1016/j.chiabu.2013.03.014 23643388

[185] Charak R Byllesby BM Fowler JC Sharp C Elhai JD Frueh BC. Assessment of the revised difficulties in emotion regulation scales among adolescents and adults with severe mental illness. Psychiatry Res. (2019) 279:278–83. 10.1016/j.psychres.2019.04.010 30975439

[186] Charak R Ford JD Modrowski CA Kerig PK. Polyvictimization, emotion dysregulation, symptoms of posttraumatic stress disorder, and behavioral health problems among justice-involved youth: a latent class analysis. J Abnorm Child Psychol. (2018) 47:287–98. 10.1007/s10802-018-0431-9 29654539

[187] Rauch S Foa E. Emotional processing theory (EPT) and exposure therapy for PTSD. J Contemp Psychother. (2006) 36:61–5. 10.1007/s10879-006-9008-y

[188] Woodward EC Viana AG Trent S E Raines EM Zvolensky MJ . Emotional nonacceptance, distraction coping and PTSD symptoms in a trauma-exposed adolescent inpatient sample. Cognit Ther Res. (2019) 44:412–9. 10.1007/s10608-019-10065-4

[189] Biederman J Wozniak J Martelon MK Spencer TJ Woodworth Y Joshi G . Can pediatric bipolar-I disorder be diagnosed in the context of posttraumatic stress disorder? A familial risk analysis. Psychiatry Res. (2013) 208:215–24. 10.1016/j.psychres.2013.05.011 23790757 PMC3728676

[190] Liu J Subramaniam M Ann Chong S Mahendran R. A systematic examination of cognitive emotion regulation strategies, global emotion dysregulation, and cognitive insight in relation to posttraumatic stress disorder symptoms among trauma exposed patients with early nonaffective psychosis. Psychol Trauma. (2019). 10.1037/tra0000531 31714102

[191] Ford JD Grasso D Greene C Levine J Spinazzola J van der Kolk B. Clinical significance of a proposed developmental trauma disorder diagnosis: Results of an international survey of clinicians. J Clin Psychiatry. (2013) 74:841–9. 10.4088/JCP.12m08030 24021504

[192] Nock MK. Self-injury. Ann Rev Clin Psychol. (2010) 6:339–63. 10.1146/annurev.clinpsy.121208.131258 20192787

[193] Glenn CR Klonsky ED. Nonsuicidal self-injury disorder: An empirical investigation in adolescent psychiatric patients. J Clin Child Adolesc Psychol. (2013) 42:496–507. 10.1080/15374416.2013.794699 23682597 PMC4433043

[194] Suyemoto KL. The functions of self-mutilation. Clin Psychol Rev. (1998) 18:531–54. 10.1016/S0272-7358(97)00105-0 9740977

[195] Gratz KL Roemer L. The relationship between emotion dysregulation and deliberate self-harm among female undergraduate students at an urban commuter university. Cogn Behav Ther. (2008) 37:14–25. 10.1080/16506070701819524 18365795

[196] Nock MK Favazza AR. Nonsuicidal self-injury: definition classification. In: Nock MK, editor. Understanding Nonsuicidal Self-Injury: Origins, Assessment Treatment. Washington, DC: American Psychological Association (2009). 10.1037/11875-001

[197] Nock MK Prinstein MJ. Contextual features and behavioral functions of self-mutilation among adolescents. J Abnorm Psychol. (2005) 114:140–6. 10.1037/0021-843X.114.1.140 15709820

[198] Chapman AL Gratz KL Brown MZ. Solving the puzzle of deliberate self-harm: The experiential avoidance model. Behav Res Ther. (2016) 44:371–94. 10.1016/j.brat.2005.03.005 16446150

[199] Nock MK Prinstein MJ Sterba KS. Revealing the form and function of self-injurious thoughts and behaviors: A real-time ecological assessment study among adolescents and young adults. J Abnorm Psychol. (2009) 118:816–27. 10.1037/a0016948 19899851 PMC5258190

[200] Caro-Cañizares I Díaz de Neira-Hernando M Pfang B Baca-Garcia E Carballo JJ. The strengths and difficulties questionnaire -dysregulation profile, non-suicidal self-injury behaviors and the mediating role of stressful life events. Span J Psychol. (2018) 21:E22. 10.1017/sjp.2018.23 29897027

[201] Nock MK Joiner TE Gordon KH Lloyd-Richardson E Prinstein MJ. Non-suicidal self-injury among adolescents: Diagnostic correlates and relation to suicide attempts. Psychiatry Res. (2006) 144:65–72. 10.1016/j.psychres.2006.05.010 16887199

[202] Andover MS Blair W Morris BW Wren A Bruzzese ME. The co-occurrence of non-suicidal self-injury and attempted suicide among adolescents: distinguishing risk factors and psychosocial correlates. Child Adolesc Psychiatry Ment Health. (2012) 6:11. 10.1186/1753-2000-6-11 22463065 PMC3379960

[203] Rajappa K Gallagher M Miranda R. Emotion dysregulation and vulnerability to suicidal ideation and attempts. Cognit Ther Res. (2011) 36:833–9. 10.1007/s10608-011-9419-2

[204] Perez J Venta A Garnaat S Sharp C. The difficulties in emotion regulation scale: Factor structure and association with nonsuicidal self-injury in adolescent inpatients. J Psychopathol Behav Assess. (2012) 34:393–404. 10.1007/s10862-012-9292-7

[205] Santangelo PS Koenig J Funke V Parzer P Resch F Ebner-Priemer UW . Ecological momentary assessment of affective and interpersonal instability in adolescent non-suicidal self-injury. J Abnorm Child Psychol. (2016) 45:1429–38. 10.1007/s10802-016-0249-2 27995358

[206] Somma A Sharp C Borroni S Fossati A. Borderline personality disorder features, emotion dysregulation and non-suicidal self-injury: Preliminary findings in a sample of community-dwelling Italian adolescents. Personal Ment Health. (2016) 11:23–32. 10.1002/pmh.1353 27910261

[207] Gratz KL Chapman AL. The role of emotional responding and childhood maltreatment in the development and maintenance of deliberate self-harm among male undergraduates. Psychol Men Masculinity. (2007) 8:1–14. 10.1037/1524-9220.8.1.1

[208] Adrian M Zeman J Veits G. Methodological implications of the affect revolution: a 35-year review of emotion regulation assessmentin children. J Exp Child Psychol. (2011) 110:171–97. 10.1016/j.jecp.2011.03.009 21514596

[209] Ann Emery A Heath NL Mills DJ. Basic psychological need satisfaction, emotion dysregulation, and non-suicidal self-injury engagement in young adults: An application of self-determination theory. J Youth Adolesc. (2015) 45:612–23. 10.1007/s10964-015-0405-y 26685906

[210] Bjureberg J Sahlin H Hedman-Lagerlöf E Gratz KL Tull TMT Jokinen J . Extending research on emotion regulation individual therapy for adolescents (ERITA) with nonsuicidal self-injury disorder: open pilot trial and mediation analysis of a novel online version. BMC Psychiatry. (2018) 18:6. 10.1186/s12888-018-1885-6 30305103 PMC6180600

[211] Sadeh N Londahl-Shaller EA Piatigorsky A Fordwood S Stuart BK McNiel DE . Functions of non-suicidal self-injury in adolescents and young adults with borderline personality disorder symptoms. Psychiatry Res. (2014) 216:217–22. 10.1016/j.psychres.2014.02.018 24594204

[212] Nakar O Brunner R Schilling O Chanen A Fischer G Parzer P . Developmental trajectories of self-injurious behavior, suicidal behavior and substance misuse and their association with adolescent borderline personality pathology. J Affect Disord. (2016) 197:231–8. 10.1016/j.jad.2016.03.029 26995466

[213] Muehlenkamp J Brausch A Quigley K Whitlock J. Interpersonal features and functions of nonsuicidal self-injury. Suicide Life-Threatening Behav. (2012) 43:67–80. 10.1111/j.1943-278X.2012.00128.x 23082783

[214] Klonsky ED Glenn CR Styer DM Olino TM Washburn JJ. The functions of nonsuicidal self-injury: converging evidence for a two-factor structure. Child Adolesc Psychiatry Ment Health. (2015) 9:73. 10.1186/s13034-015-0073-4 26421059 PMC4586000

[215] Plener PL Kapusta ND Kölch MG Kaess M Brunner R. Nicht-suizidale Selbstverletzung als eigenständige Diagnose. [Non-suicidal self-injury as autonomous diagnosis - implications for research and clinic of the DSM-5 proposal to establish the diagnosis of non-suicidal self-injury in adolescents]. Zeitschrift für Kinder- und Jugendpsychiatrie und Psychotherapie. (2012) 40:113–20. 10.1024/1422-4917/a000158 22354495

[216] Turner BJ Yiu A Layden BK Claes L Zaitsoff S Chapman AL. Temporal associations between disordered eating and nonsuicidal self-injury: Examining symptom overlap over 1 year. Behav Ther. (2015) 46:125–38. 10.1016/j.beth.2014.09.002 25526840

[217] Duggan J Heath N Hu T. Non-suicidal self-injury maintenance and cessation among adolescents: a one-year longitudinal investigation of the role of objectified body consciousness, depression and emotion dysregulation. Child Adolesc Psychiatry Ment Health. (2015) 9: 52. 10.1186/s13034-015-0052-9 26157480 PMC4495797

[218] Adrian M McCauley E Berk MS Asarnow JR Korslund K Avina C . Predictors and moderators of recurring self-harm in adolescents participating in a comparative treatment trial of psychological interventions. J Child Psychol Psychiatry. (2019) 60:1123–32. 10.1111/jcpp.13099 31359435 PMC6849475

[219] Hilt LM Cha CB Nolen-Hoeksema S. Nonsuicidal self-injury in young adolescent girls: Moderators of the distress-function relationship. J Consult Clin Psychol. (2008) 76:63–71. 10.1037/0022-006X.76.1.63 18229984

[220] Crowell SE Beauchaine TP Linehan M M. A biosocial developmental model of borderline personality: Elaborating and extending Linehan's theory. Psychol Bull. (2009) 135:495–510. 10.1037/a0015616 19379027 PMC2696274

[221] Sim L Adrian M Zeman J Cassano M Friedrich WN. Adolescent deliberate self-harm: Linkages to emotion regulation and family emotional climate. J Res Adolesc. (2009) 19:75–91. 10.1111/j.1532-7795.2009.00582.x

[222] Kaess M Parzer P Mattern M Plener PL Bifulco A Resch F . Adverse childhood experiences and their impact on frequency, severity, and the individual function of nonsuicidal self-injury in youth. Psychiatry Res. (2013) 206:265–72. 10.1016/j.psychres.2012.10.012 23159195

[223] Titelius EN Cook E Spas J Orchowski L Kivisto K O'Brien K . Emotion dysregulation mediates the relationship between child maltreatment and non-suicidal self-injury. J Aggress Maltreatment Trauma. (2017) 27:323–31. 10.1080/10926771.2017.1338814 30369785 PMC6108548

[224] Fraser G Stewart Wilson M Anne Garisch J Robinson K Brocklesby M Kingi T . Non-suicidal self-injury, sexuality concerns, and emotion regulation among sexually diverse adolescents: A multiple mediation analysis. Arch Suicide Res. (2017) 22:432–52. 10.1080/13811118.2017.1358224 28759324

[225] Ybarra ML Mitchell KJ Kosciw JG Korchmaros JD. Understanding linkages between bullying and suicidal ideation in a national sample of LGB and heterosexual youth in the United States. Prevent Sci. (2014) 16:451–62. 10.1007/s11121-014-0510-2 25322949

[226] Shneidman ES. Suicide as psychache. J Nerv Ment Dis. (1993) 181:145–7. 10.1097/00005053-199303000-00001 8445372

[227] Ringel E. Der Selbstmord [The Suicide]. Maudrich, 5th edition. Wien (1993).

[228] Gliatto MF Rai AR. Evaluation and treatment of patients with suicidal ideation. Am Fam Physician. (1999) 59:1500–6. 10193592

[229] Borges G Nock MK Haro Abad JM Hwang I Sampson NA Alonso J . Twelve-month prevalence of and risk factors for suicide attempts in the World Health Organization world mental health surveys. J Clin Psychiatry. (2010) 71:1617–28. 10.4088/JCP.08m04967blu 20816034 PMC3000886

[230] Nock M. K., Borges G, Bromet J E, Cha CB, Kessler RC, . Suicide and suicidal behavior. Epidemiol Rev. (2008) 30:133–54. 10.1093/epirev/mxn002 18653727 PMC2576496

[231] Wolff CJ Davis S Liu RT Cha CB Cheek SM. Trajectories of suicidal ideation among adolescents following psychiatric hospitalization. J Abnorm Child Psychol. (2017) 46:355–63. 10.1007/s10802-017-0293-6 28349306 PMC5617752

[232] Pan LA Hassel S Segreti AM Nau SA Brent DA Phillips ML. Differential patterns of activity and functional connectivity in emotion processing neural circuitry to angry and happy faces in adolescents with and without suicide attempt. Psychol Med. (2013) 43:2129–42. 10.1017/S0033291712002966 23298821

[233] Selby EA Yen S Spirito A. Time varying prediction of thoughts of death and suicidal ideation in adolescents: Weekly ratings over 6-month follow-up. J Clin Child Adolesc Psychol. (2013) 42:481–95. 10.1080/15374416.2012.736356 23148530 PMC3584233

[234] Tamás Z Kovacs M Gentzler AL Tepper P Gádoros J Kiss E . The relations of temperament and emotion self-regulation with suicidal behaviors in a clinical sample of depressed children in Hungary. J Abnorm Child Psychol. (2007) 35:640–52. 10.1007/s10802-007-9119-2 17530394

[235] Saffer BY Glenn CR Klonsky ED. Clarifying the relationship of parental bonding to suicide ideation and attempts. Suicide Life-Threatening Behav. (2014) 45:518–28. 10.1111/sltb.12146 25530006

[236] Anestis MD Kleiman EM Lavender JM Tull MT Gratz KL. The pursuit of death versus escape from negative affect: An examination of the nature of the relationship between emotion dysregulation and both suicidal behavior and non-suicidal self-injury. Compr Psychiatry. (2014) 55:1820–30. 10.1016/j.comppsych.2014.07.007 25104613

[237] Esposito C Spirito A Boergers J Donaldson D. Affective, behavioral, and cognitive functioning in adolescents with multiple suicide attempts. Suicide Life Threatening Behav. (2003) 33:389–99. 10.1521/suli.33.4.389.25231 14695054

[238] World Health Organization. Icd-10 (International Statistical Classification of Diseases and Health Related Problems) (2019).

[239] Wisniewski L Safer D Chen E. Dialectical behavior therapy and eating disorders. In: Dimeff LA Koerner K, editors. Dialectical Behavior Therapy in Clinical Practice: Applications Across Disorders and Settings. New York, NY: The Guilford Press (2007).

[240] Knatz S Braden A Kerri N. Boutelle. Parent coaching model for adolescents with emotional eating. Eat Disord. (2015) 23:377–86. 10.1080/10640266.2015.1044352 26011794

[241] Racine SE Wildes JE. Emotion dysregulation and symptoms of anorexia nervosa: The unique roles of lack of emotional awareness and impulse control difficulties when upset. Int J Eat Disord. (2013) 46:713–20. 10.1002/eat.22145 23754665 PMC3784620

[242] Cimbolli P Quiñones Á Ugarte C De Pascale A. Studio pilota sui disturbi della nutrizione e dell'alimentazione in età pediatrica e i disturbi dell'umore: comorbilità o tratti prodromici? Riv Psichiatr. (2017) 52:32–9. 10.1708/2631.27052 28287195

[243] Haynos AF Fruzzetti AE. Anorexia nervosa as a disorder of emotion dysregulation: Evidence and treatment implications. Clin Psychol. (2011) 18:183–202. 10.1111/j.1468-2850.2011.01250.x

[244] Monell E Clinton D Birgegård A. Emotion dysregulation and eating disorders- associations with diagnostic presentation and key symptoms. Int J Eat Disord. (2018) 51:921–30. 10.1002/eat.22925 30030942

[245] Safer DL Telch CG Chen EY. Dialectical Behavior Therapy for Binge Eating and Bulimia. New York, NY: The Guilford Press (2009).

[246] Guerdjikova AI McElroy SL Kotwal R Stanford K Keck PE. Psychiatric and metabolic characteristics of childhood versus adult-onset obesity in patients seeking weight management. Eat Behav. (2007) 8:266–76. 10.1016/j.eatbeh.2006.11.001 17336797

[247] Lavender JM Wonderlich SA Peterson CB Crosby RD Engel SG Mitchell JE . Dimensions of emotion dysregulation in bulimia nervosa. Eur Eat Disord Rev. (2014) 22:212–6. 10.1002/erv.2288 24619484 PMC4554700

[248] Hansson E Daukantaité D Johnsson P. Disordered eating and emotion dysregulation among adolescents and their parents. BMC Psychol. (2017) 5:180. 10.1186/s40359-017-0180-5 28376909 PMC5381147

[249] Hughes-Scalise A Connell A. The roles of adolescent attentional bias and parental invalidation of sadness in significant illness: A comparison between eating disorders and chronic pain. Eat Behav. (2014) 15:493–501. 10.1016/j.eatbeh.2014.06.007 25064305

[250] Gilboa-Schechtman E Avnon L Zubery E Jeczmien P. Emotional processing in eating disorders: specific impairment or general distress related deficiency? Depress Anxiety. (2006) 23:331–9. 10.1002/da.20163 16688732

[251] Gilbert KE. The neglected role of positive emotion in adolescent psychopathology. Clin Psychol Rev. (2012) 32:467–81. 10.1016/j.cpr.2012.05.005 22710138

[252] Racine SE Wildes JE. Dynamic longitudinal relations between emotion regulation difficulties and anorexia nervosa symptoms over the year following intensive treatment. J Consult Clin Psychol. (2015) 83:785–95. 10.1037/ccp0000011 25181027 PMC4345157

[253] Mills P Frances Newman E Jill Cossar J George Murray G. Emotional maltreatment and disordered eating in adolescents: Testing the mediating role of emotion regulation. Child Abuse Neglect. (2015) 39:156–66. 10.1016/j.chiabu.2014.05.011 25129874

[254] Hansson E Daukantaite D Johnsson P. Typical patterns of disordered eating among Swedish adolescents: associations with emotion dysregulation, depression, and self-esteem. J Eat Disord. (2016) 4:28. 10.1186/s40337-016-0122-2 27822375 PMC5097389

[255] Anderson LK Claudat K Cusack A Brown TA Trim J Rockwell R. Differences in emotion regulation difficulties among adults and adolescents across eating disorder diagnoses. J Clin Psychol. (2018) 74:1867–73. 10.1002/jclp.22638 29756232

[256] Jakovina T Crnkovic Batista M Razic Pavicic A Zuric Jakovina I Begovac I. Emotional dysregulation and attachment dimensions in female patients with bulimia nervosa. Psychiatr Danub. (2018) 30:72–8. 10.24869/psyd.2018.72 29546861

[257] Laghi F Bianchi D Pompili S Lonigro A Baiocco R. Metacognition, emotional functioning and binge eating in adolescence: the moderation role of need to control thoughts. Eat Weight Disord. (2018) 23:861–9. 10.1007/s40519-018-0603-1 30367384

[258] Boutelle KN Braden A Knatz-Peck S Anderson LK Rhee KE. An open trial targeting emotional eating among adolescents with overweight or obesity. Eat Disord. (2018) 26:79–91. 10.1080/10640266.2018.1418252 29384462

[259] Wiser S Telch CF. Dialectical behavior therapy for binge-eating disorder. J Clin Psychol. (1999) 55:755–68. 10.1002/(SICI)1097-4679(199906)55:6<755::AID-JCLP8>3.0.CO;2-R 10445865

[260] Segal A. Differences in emotion regulation along the eating disorder spectrum: Cross sectional study in adolescents out patient care. J Psychol Clin Psychiatry. (2016) 6:314. 10.15406/jpcpy.2016.06.00314

[261] Rai T Mainali P Raza A Rashid J Rutkofsky I. Exploring the link between emotional child abuse and anorexia nervosa: A psychopathological correlation. Cureus. (2019) 11:e5318. 10.7759/cureus.5318 31598427 PMC6777933

[262] McDonald CE Rossell SL Phillipou A. The comorbidity of eating disorders in bipolar disorder and associated clinical correlates characterised by emotion dysregulation and impulsivity: A systematic review. J Affect Disord. (2019) 259:228–43. 10.1016/j.jad.2019.08.070 31446385

[263] Slane JD Klump KL McGue M Iacono G. Genetic and environmental factors underlying comorbid bulimic behaviours and alcohol use disorders: A moderating role for the dysregulated personality cluster? Eur Eat Disord Rev. (2014) 22:159–69. 10.1002/erv.2284 24616026 PMC4255714

[264] Ford JD. Traumatic victimization in childhood and persistent problems with oppositional defiance. J Aggress Maltreatment Trauma. (2002) 6:25–58. 10.1300/J146v06n01_03

[265] Steiner H Remsing L. Practice parameter for the assessment and treatment of children and adolescents with oppositional defiant disorder. J Am Acad Child Adolesc Psychiatry. (2007) 46:126–41. 10.1097/01.chi.0000246060.62706.af 17195736

[266] Déry M Lapalme M Jagiellowicz J Poirier M Temcheff C Toupin J. Predicting depression and anxiety from oppositional defiant disorder symptoms in elementary school-age girls and boys with conduct problems. Child Psychiatry Hum Dev. (2016) 48:53–62. 10.1007/s10578-016-0652-5 27209374

[267] Muratori P Pisano S Milone A Masi G. Is emotional dysregulation a risk indicator for auto-aggression behaviors in adolescents with oppositional defiant disorder? J Affect Disord. (2017) 208:110–2. 10.1016/j.jad.2016.08.052 27764737

[268] Bradshaw CP Mitchell MM Leaf PJ. Examining the effects of schoolwide positive behavioral interventions and supports on student outcomes. J Posit Behav Interv. (2009) 12:133–48. 10.1177/1098300709334798

[269] Blandon AY Calkins SD Grimm J K Keane SP O'Brien M. Testing a developmental cascade model of emotional and social competence and early peer acceptance. Dev Psychopathol. (2010) 22:737–48. 10.1017/S0954579410000428 20883578 PMC3019307

[270] Martel MM Gremillion ML Roberts B. Temperament and common disruptive behavior problems in preschool. Pers Individ Dif. (2012) 53:874–9. 10.1016/j.paid.2012.07.011 23139437 PMC3489497

[271] Cavanagh M Quinn D Duncan D Graham T Balbuena L. Oppositional defiant disorder is better conceptualized as a disorder of emotional regulation. J Atten Disord. (2016) 21:381–9. 10.1177/1087054713520221 24481934

[272] Frick MA Forslund T Fransson M Johansson M Bohlin G Brocki KC. The role of sustained attention, maternal sensitivity, and infant temperament in the development of early self-regulation. Br J Psychol. (2018) 109:277–98. 10.1111/bjop.12266 28895129

[273] Marion Mitchison G Margo Liber J Hannesdottir D Njardvik U. Emotion dysregulation, ODD and conduct problems in a sample of five and six-year-old children. Child Psychiatry Hum Dev. (2019) 51:71–9. 10.1007/s10578-019-00911-7 31300966

[274] Sagar-Ouriaghli I Lievesley K Santosh PJ. Propranolol for treating emotional, behavioural, autonomic dysregulation in children and adolescents with autism spectrum disorders. J Psychopharmacolo. (2018) 32:641–53. 10.1177/0269881118756245 29484909

[275] Fehlbaum LV Raschle NM Menks WM Prätzlich M Flemming E. Altered neuronal responses during an affective stroop task in adolescents with conduct disorder. Front Psychol. (2018) 9.:1961 10.3389/fpsyg.2018.01961 30405475 PMC6200838

[276] Schoorl J van Rijn S de Wied M van Goozen S Swaab H. Emotion regulation difficulties in boys with oppositional defiant disorder/conduct disorder and the relation with comorbid autism traits and attention deficit traits. PLoS ONE. (2016) 11:e0159323. 10.1371/journal.pone.0159323 27420110 PMC4946778

[277] Bauermeister JJ Shrout PE Ramírez R Bravo M Alegría M Martínez-Taboas A . ADHD correlates, comorbidity, and impairment in community and treated samples of children and adolescents. J Abnorm Child Psychol. (2007) 35:883–98. 10.1007/s10802-007-9141-4 17505876 PMC3591788

[278] Leaberry KD Rosen PJ Fogleman ND Walerius DM Slaughter KE. Comorbid internalizing and externalizing disorders predict lability of negative emotions among children with ADHD. J Atten Disord. (2020) 24:2001. 10.1177/1087054717734647 28992752

[279] Ford JD Connor DF Hawke J. Complex trauma among psychiatrically impaired children: a cross-sectional, chart-review study. J Clin Psychiatry. (2009) 70:1155–63. 10.4088/JCP.08m04783 19573498

[280] Ford JD Fraleigh LA Connor DF. Child abuse and aggression among seriously emotionally disturbed children. J Clin Child Adolesc Psychol. (2010) 39:25–34. 10.1080/15374410903401104 20390796

[281] Ford JD Racusin R Daviss WB Ellis CG Thomas J Rogers K . Trauma exposure among children with oppositional defiant disorder and attention deficit-hyperactivity disorder. J Consult Clin Psychol. (1999) 67:786–9. 10.1037/0022-006X.67.5.786 10535245

[282] Vasileva M Petermann U Petermann F. Traumatische Erfahrungen und Callous -unemotional Traits: Zusammenhang mit funktionalen und dysfunktionalen Emotionsregulationsstrategien. Zeitschrift für Psychiatrie Psychologie und Psychotherapie. (2019) 67:125–32. 10.1024/1661-4747/a000380

[283] Hoskins D Marshall BDL Koinis-Mitchell D Galbraith K Tolou-Shams M. Latinx youth in first contact with the justice system: Trauma and associated behavioral health needs. Child Psychiatry Hum Dev. (2018) 50:459–72. 10.1007/s10578-018-0855-z 30483922 PMC6482072

[284] Cooley JL Ritschel LA Frazer AL Blossom JB. The influence of internalizing symptoms and emotion dysregulation on the association between witnessed community violence and aggression among urban adolescents. Child Psychiatry Hum Dev. (2019) 50:883–93. 10.1007/s10578-019-00890-9 30989477 PMC6790286

[285] Plattner B Karnik N Jo B Hall RE Schallauer A Carrion V . State and trait emotions in delinquent adolescents. Child Psychiatry Hum Dev. (2007) 38:155–69. 10.1007/s10578-007-0050-0 17417724

[286] Landis TD Garcia AM Hart KC Graziano PA. Differentiating symptoms of ADHD in preschoolers: the role of emotion regulation and executive function. J Atten Disord. (2021) 25:1260–71. 10.1177/1087054719896858 31904270 PMC9104514

[287] Masi G Pisano S Milone A Muratori P. Child behavior checklist dysregulation profile in children with disruptive behavior disorders. A longitudinal study. J Affect Disord. (2015) 186:249–53. 10.1016/j.jad.2015.05.069 26254616

[288] Masi G Muratori P Manfredi A Pisano S Milone A. Child behaviour checklist emotional dysregulation profiles in youth with disruptive behaviour disorders: Clinical correlates and treatment implications. Psychiatry Res. (2015) 225:191–6. 10.1016/j.psychres.2014.11.019 25480545

[289] Tufan E Topal Z Demir N Taskiran S Savci U Akif Cansiz M . Sociodemographic and clinical features of disruptive mood dysregulation disorder: A chart review. J Child Adolesc Psychopharmacol. (2016) 26:94–100. 10.1089/cap.2015.0004 26491995

[290] Martin SE Hunt JI Mernick LR DeMarco M Hunter HL Coutinho T . Temper loss and persistent irritability in preschoolers: Implications for diagnosing disruptive mood dysregulation disorder in early childhood. Child Psychiatry Hum Dev. (2016) 48:498–508. 10.1007/s10578-016-0676-x 27510439

[291] Dickerson Mayes S Kokotovich C Mathiowetz C Baweja R Calhoun SL Waxmonsky J. Disruptive mood dysregulation disorder symptoms by age in autism, ADHD and general population samples. J Ment Health Res Intellect Disabil. (2017) 10:345–59. 10.1080/19315864.2017.133880427549781

[292] Dougherty LR Smith VC Bufferd SJ Carlson A G Stringaris A . DSM-5 disruptive mood dysregulation disorder: correlates and predictors in young children. Psychol Med. (2014) 44:2339–50. 10.1017/S0033291713003115 24443797 PMC4480202

[293] Zepf D F Biskup CS Holtmann M Runions K. Disruptive mood dysregulation disorder. In: Rey JM, editor. IACAPAP e-Textbook of Child and Adolescent Mental Health. Geneva: International Association for Child and Adolescent Psychiatry and Allied Professions (2016).

[294] Adleman NE Fromm SJ Razdan V Kayser R Dickstein PD. Cross-sectional and longitudinal abnormalities in brain structure in children with severe mood dysregulation or bipolar disorder. J Child Psychol Psychiatry. (2012) 53:1149–56. 10.1111/j.1469-7610.2012.02568.x 22650379 PMC3472043

[295] Deveney CM Connolly ME Jenkins SE Kim P Fromm SJ Pine DS. Neural recruitment during failed motor inhibition differentiates youths with bipolar disorder and severe mood dysregulation. Biol Psychol. (2012) 89:148–55. 10.1016/j.biopsycho.2011.10.003 22008364 PMC3245776

[296] El-Rasheed AH ElAttar KS Elrassas HH Mahmoud DAM Mohamed SY. Mood regulation, alexithymia, and personality disorders in adolescent male addicts. Addictive Disorders and Their Treatment. (2017) 16:49–58. 10.1097/ADT.0000000000000098

[297] Picardi A Toni A Caroppo E. Stability of alexithymia and its relationships with the 'big five' factors, temperament, character, attachment style. Psychother Psychosom. (2005) 74:371–8. 10.1159/000087785 16244514

[298] Narimani M Vahidi Z Abolghasemi A. Comparison alexithymia, impulsiovity and activation and inhibitiobn of the students with symptoms of obsessive - compulsive and paranoid personality disorder with normal individuals. J Clin Psychol. (2013) 5:55–65. 10.22075/jcp.2017.2127

[299] Reeves M James LM Pizzarello SM Taylor JE. Support for Linehan's biosocial theory from a nonclinical sample. J Pers Disord. (2010) 24:312–26. 10.1521/pedi.2010.24.3.312 20545497

[300] Sharp C Pane H Ha C Venta A Patel AB Sturek J . Theory of mind and emotion regulation difficulties in adolescents with borderline traits. J Am Acad Child Adolesc Psychiatry. (2011) 50:563–73.el. 10.1016/j.jaac.2011.01.017 21621140

[301] Carpenter RW Trull TJ. Components of emotion dysregulation in borderline personality disorder: A review. Curr Psychiatry Rep. (2012) 15:335. 10.1007/s11920-012-0335-2 23250816 PMC3973423

[302] Goodman M Hazlett EA Avedon JB Siever RD Chu S New A. Anterior cingulate volume reduction in adolescents with borderline personality disorder and co-morbid major depression. J Psychiatr Res. (2011) 45:803–7. 10.1016/j.jpsychires.2010.11.011 21145068

[303] Xenaki LA Pehlivanidis A. Clinical, neuropsychological and structural convergences and divergences between attention deficit/hyperactivity disorder and borderline personality disorder: A systematic review. Pers Individ Dif. (2015) 86:438–49. 10.1016/j.paid.2015.06.049

[304] Krauch M Ueltzhöffer K Brunner R Kaess M Hensel S Herpertz SC . Heightened salience of anger and aggression in female adolescents with borderline personality disorder-a script-based fMRI study. Front Behav Neurosci. (2018) 12:57. 10.3389/fnbeh.2018.00057 29632476 PMC5879116

[305] Fonagy P Luyten P. A developmental, mentalization-based approach to the understanding and treatment of borderline personality disorder. Dev Psychopathol. (2009) 21:1355–81. 10.1017/S0954579409990198 19825272

[306] Selby EA Anestis MD Bender TW Joiner TE. An exploration of the emotional cascade model in borderline personality disorder. J Abnorm Psychol. (2009) 118:375–87. 10.1037/a0015711 19413411 PMC2842601

[307] Fonagy P Target M Gergely G Allen JG Bateman AW. The developmental roots of borderline personality disorder in early attachment relationships: A theory and some evidence. Psychoanalytic Inquiry. (2003) 23:412–59. 10.1080/07351692309349042

[308] Kernberg O. Borderline personality organization. J Am Psychoanal Assoc. (1967) 15:641–85. 10.1177/000306516701500309 4861171

[309] Gratz KL Kiel EJ Latzman RD Elkin D T Anne Moore S. Emotion: Empirical contribution: Maternal borderline personality pathology and infant emotion regulation: Examining the influence of maternal emotion-related difficulties and infant attachment. J Pers Disord. (2014) 28:52–69. 10.1521/pedi.2014.28.1.52 24344887

[310] Kim S Sharp C Carbone C. The protective role of attachment security for adolescent borderline personality disorder features via enhanced positive emotion regulation strategies. Personality Disord. (2014) 5:125–36. 10.1037/per0000038 24364499

[311] Crawford TN Livesley WJ Jang KL Shaver PR Cohen P Ganiban J. Insecure attachment and personality disorder: a twin study of adults. Eur J Pers. (2007) 21:191–208. 10.1002/per.602

[312] Sharp C Venta A Vanwoerden S Schramm A Ha C Newlin E. First empirical evaluation of the link between attachment, social cognition and borderline features in adolescents. Compr Psychiatry. (2016) 64:4–11. 10.1016/j.comppsych.2015.07.008 26298843

[313] Kalpakci A Vanwoerden S Elhai JD Sharp C. The independent contributions of emotion dysregulation and hypermentalization to the “double dissociation” of affective and cognitive empathy in female adolescent inpatients with BPD. J Pers Disord. (2016) 30:242–60. 10.1521/pedi_2015_29_192 25905730

[314] Lenzenweger MF Clarkin JF Fertuck EA Kernberg OF. Executive neurocognitive functioning and neurobehavioral systems indicators in borderline personality disorder: A preliminary study. J Pers Disord. (2004) 18:421–38. 10.1521/pedi.18.5.421.51323 15519953

[315] Jayaro C De La Vega I Bayon-Palomino C Díaz-Marsá M Montes A Tajima K . Depressive-type emotional response pattern in impulsive-aggressive patients with borderline personality disorder. J Affect Disord. (2011) 135:37–42. 10.1016/j.jad.2011.06.040 21807413

[316] Schramm AT Venta A Sharp C. The role of experiential avoidance in the association between borderline features and emotion regulation in adolescents. Personal Disord. (2013) 4:138–44. 10.1037/a0031389 23397937

[317] Fossati A Gratz KL Maffei C Borroni S. Impulsivity dimensions, emotion dysregulation, and borderline personality disorder featuresamong Italian nonclinical adolescents. Borderline Personality Disorder Emotion Dysregulation. (2014) 1:1–11. 10.1186/2051-6673-1-5 26401289 PMC4574387

[318] Yen S Gagnon K Spirito A. Borderline personality disorder in suicidal adolescents. Personal Ment Health. (2012) 7:89–101. 10.1002/pmh.1216 24343935 PMC4414329

[319] Mancke F Herpertz SC Bertsch K. Aggression in borderline personality disorder: A multidimensional model. Personality Disord. (2015) 6:278–91. 10.1037/per0000098 26191822

[320] Mancke I Herpertz SC Kleindienst N Bertsch K. Emotion dysregulation and trait anger sequentially mediate the association between borderline personality disorder and aggression. J Pers Disord. (2017) 31:256–272. 10.1521/pedi_2016_30_247 27064852

[321] Banny AM Tseng WL Murray-Close D Pitula CE Crick NR. Borderline personality features as a predictor of forms and functions of aggression during middle child-hood: Examining the roles of gender and physiological reactivity. Dev Psychopathol. (2014) 26:789–804. 10.1017/S095457941400039X 25047299

[322] Gaher RM Hofman NL Simons JS Hunsaker R. Emotion regulation deficits as mediators between trauma exposure and borderline symptoms. Cognit Ther Res. (2013) 37:466–75. 10.1007/s10608-012-9515-y

[323] van Dijke A Ford JD van der Hart L van Son JD Maarten F. Association of childhood-trauma-by-primary caregiver and affect dysregulation with borderline personality disorder symptoms in adulthood. Psychol Trauma Theory Res Practice Policy. (2013) 5:217–24. 10.1037/a0027256

[324] Ford JD Courtois CA. Complex PTSD and borderline personality disorder. Borderl. Personal Disord. Emotion Dysregul. (2021) 8:16. 10.1186/s40479-021-00155-9 33958001 PMC8103648

[325] Yen S Frazier E Hower H Weinstock LM Topor DR Hunt J . Borderline personality disorder in transition age youth with bipolar disorder. Acta Psychiatr Scand. (2015) 132:270–80. 10.1111/acps.12415 25865120 PMC4573347

[326] Winsper C Tang NKY. Linkages between insomnia and suicidality: Prospective associations, high-risk subgroups and possible psychological mechanisms. Int Rev Psychiatry. (2014) 26:189–204. 10.3109/09540261.2014.881330 24892894

[327] Marco JH Pérez S García-Alandete J Moliner R. Meaning in life in people with borderline personality disorder. Clin Psychol Psychother. (2015) 24:162–70. 10.1002/cpp.1991 26639791

[328] Stanton K Rozek DC Stasik-O'Brien SM Ellickson-Larew S Watson DA. Transdiagnostic approach to examining the incremental predictive power of emotion regulation and basic personality dimensions. J Abnorm Psychol. (2016) 125:960–75. 10.1037/abn0000208 27732026

[329] Cheshure A Zeigler-Hill V Sauls D Vrabel JK Lehtman MJ. Narcissism and emotion dysregulation: Narcissistic admiration and narcissistic rivalry have divergent associations with emotion regulation difficulties. Pers Individ Dif. (2020) 154:109679. 10.1016/j.paid.2019.109679

[330] Back MD Küfner ACP Dufner M Gerlach TM Rauthmann JF. Narcissistic admiration and rivalry: Disentangling the bright and dark sides of narcissism. J Pers Soc Psychol. (2013) 105:1013–37. 10.1037/a0034431 24128186

[331] Yang Y Narr KL Baker LA Joshi SH Jahanshad N Raine A . Frontal and striatal alterations associated with psychopathic traits in adolescents. Psychiatry Res. (2015) 231:333–40. 10.1016/j.pscychresns.2015.01.017 25676553 PMC4871259

[332] Wills TA Simons SJ Sussman S Knight R. Emotional self-control and dysregulation: A dual-process analysis of pathways to externalizing/internalizing symptomatology and positive well-being in younger adolescents. Drug Alcohol Depend. (2016) 163:S37–45. 10.1016/j.drugalcdep.2015.08.039 27306730 PMC4911542

[333] Englund MM Egeland B Oliva EM Collins WA. Childhood and adolescent predictors of heavy drinking and alcohol use disorders in early adulthood: a longitudinal developmental analysis. Addiction. (2008) 103:23–35. 10.1111/j.1360-0443.2008.02174.x 18426538 PMC2822999

[334] Boulos PK Dalwani MS Tanabe J Mikulich-Gilbertson KS Banich MT . Brain cortical thickness differences in adolescent females with substance use disorders. PLoS ONE. (2016) 11:e0152983. 10.1371/journal.pone.0152983 27049765 PMC4822952

[335] Tan H Ahmad T Loureiro M Zunder J Laviolette SR. The role of cannabinoid transmission in emotional memory formation: Implications for addiction and schizophrenia. Front Psychiatry. (2014) 5:73. 10.3389/fpsyt.2014.00073 25071606 PMC4074769

[336] Ilbegi S Groenman AP Schellekens A Hartman CA Hoekstra PJ Franke B. Substance use and nicotine dependence in persistent, remittent, and late-onset ADHD: a 10-year longitudinal study from childhood to young adulthood. J Neurodev Disord. (2018) 10:9260. 10.1186/s11689-018-9260-y 30587104 PMC6307241

[337] Hasler BP Soehner AM Clark DB. Sleep and circadian contributions to adolescent alcohol use disorder. Alcohol. (2015) 49:377–87. 10.1016/j.alcohol.2014.06.010 25442171 PMC4424185

[338] Cheetham A Allen NB Yücel M Lubman DI. The role of affective dysregulation in drug addiction. Clin Psychol Rev. (2020) 30:621–34. 10.1016/j.cpr.2010.04.005 20546986

[339] Simons JS Carey KB. Risk and vulnerability for marijuana use problems: The role of affect dysregulation. Psychol Addict Behav. (2002) 16:72–5. 10.1037/0893-164X.16.1.72 11934090

[340] Brown LK Houck C Lescano C Donenberg G Tolou-Shams M Mello J. Affect regulation and HIV risk among youth in therapeutic schools. AIDS Behav. (2012) 16:2272–8. 10.1007/s10461-012-0220-3 22669595 PMC3496428

[341] Brumback T Worley M Nguyen-Louie TT Squeglia LM Jacobus J Tapert SF. Neural predictors of alcohol use and psychopathology symptoms in adolescents. Dev Psychopathol. (2016) 28:1209–16. 10.1017/S0954579416000766 27739397 PMC5094063

[342] Weinstein SM Mermelstein R Shiffman S Flay B. Mood variability and cigarette smoking escalation among adolescents. Psychol Addict Behav. (2008) 22:504–13. 10.1037/0893-164X.22.4.504 19071975 PMC2605639

[343] Khantzian EJ. The self-medication hypothesis of addictive disorders: Focus on heroin and cocaine dependence. Am J Psychiatry. (1985) 142:1259–64. 10.1176/ajp.142.11.1259 3904487

[344] Brook JS Zhang C Leukefeld CG Brook DW. Marijuana use from adolescence to adulthood: developmental trajectories and their outcomes. Soc Psychiatry Psychiatr Epidemiol. (2016) 51:1405–15. 10.1007/s00127-016-1229-0 27168181 PMC5050063

[345] Bonn-Miller MO Vujanovic AA Zvolensky MJ. Emotional dysregulation: Association with coping-oriented marijuana use motives among current marijuana users. Substance Use Misuse. (2008) 43:1653–65. 10.1080/10826080802241292 18752166

[346] Dorard G Berthoz S Phan O Corcos M Bungener C. Affect dysregulation in cannabis abusers. Eur Child Adolesc Psychiatry. (2008) 17:274–82. 10.1007/s00787-007-0663-7 18301941

[347] Clark DB Chung T Thatcher DL Pajtek S Long EC. Psychological dysregulation, white matter disorganization and substance use disorders in adolescence. Addiction. (2011) 107:206–214. 10.1111/j.1360-0443.2011.03566.x 21752141 PMC3237873

[348] Bava S Jacobus J Thayer RE Tapert SF. Longitudinal changes in white matter integrity among adolescent substance users. Alcoholism. (2012) 37:E181–9. 10.1111/j.1530-0277.2012.01920.x 23240741 PMC3548057

[349] Coban FR Kunst AE Van Stralen MM Richter M Rathmann K Perelman J . Nicotine dependence among adolescents in the European Union: How many and who are affected? J Public Health. (2018) 41:447–55. 10.1093/pubmed/fdy136 30192963

[350] Novak SP Clayton RR. The influence of school environment and self-regulation on transitions between stages of cigarette smoking: A multilevel analysis. Health Psychol. (2001) 20:196–207. 10.1037/0278-6133.20.3.196 11403217

[351] Wilens TE Martelon MK Anderson JP Shelley-Abrahamson R Biederman J. Difficulties in emotional regulation and substance use disorders: A controlled family study of bipolar adolescents. Drug Alcohol Depend. (2013) 132:114–21. 10.1016/j.drugalcdep.2013.01.015 23422834 PMC3683118

[352] Jessica Combs LJL Nichea Spillane SSN Leann Caudill L Brittany Stark B Gregory Smith TGT. The acquired preparedness risk model applied to smoking in 5th grade children. Addict Behav. (2012) 37:331–4. 10.1016/j.addbeh.2011.11.005 22143003 PMC3711825

[353] Dir AL Banks DE Zapolski TCB McIntyre E Hulvershorn LA. Negative urgency and emotion regulation predict positive smoking expectancies in non-smoking youth. Addict Behav. (2016) 58:47–52. 10.1016/j.addbeh.2016.02.014 26905764 PMC4808417

[354] Colder CR Stice E. A longitudinal study of the interactive effects of impulsivity and anger on adolescent problem behavior. J Youth Adolesc. (1998) 27:255–74. 10.1023/A:1022889202419

[355] Mischel ER Leen-Feldner EW Knapp AA Bilsky AS Hama L. Indirect effects of smoking motives on adolescent anger dysregulation and smoking. Addict Behav. (2014) 39:1831–8. 10.1016/j.addbeh.2014.07.029 25128636

[356] Kassel JD Stroud LR Paronis CA. Smoking, stress, and negative affect: Correlation, causation, and context across stages of smoking. Psychol Bull. (2003) 129:270–304. 10.1037/0033-2909.129.2.270 12696841

[357] Mermelstein R Hedeker D Flay BR Saul Shiffman S. Real-Time Data Capture and Adolescent Cigarette Smoking. (2007). Available online at: https://www.researchgate.net/publication/265530314 (accessed July 26, 2020).

[358] Whalen CK Jamner LD Henker B Delfino RJ. Smoking and moods in adolescents with depressive and aggressive dispositions: Evidence from surveys and electronic diaries. Health Psychol. (2001) 20:99–111. 10.1037/0278-6133.20.2.99 11315734

[359] Weinstein SM Mermelstein RJ. Dynamic associations of negative mood and smoking across the development of smoking in adolescence. J Clin Child Adolesc Psychol. (2013) 42:629–42. 10.1080/15374416.2013.794698 23682640 PMC3762940

[360] Treloar Padovano H Merrill JE Colby SM Kahler CW Gwaltney CJ. Affective and situational precipitants of smoking lapses among adolescents. Nicotine Tobacco Res. (2019) 22:492–7. 10.1093/ntr/ntz002 30624745 PMC7164579

[361] Kovacs M Sherrill J George JC Pollock M Tumuluru RV. Contextual emotion-regulation therapy for childhood depression: Description and pilot testing of a new intervention. J Am Acad Child Adolesc Psychiatry. (2006) 45:892–903. 10.1097/01.chi.0000222878.74162.5a 16865031

[362] Banducci AN Hoffman EM Lejuez CW Koenen KC. The impact of childhood abuse on inpatient substance users: Specific links with risky sex, aggression, emotion dysregulation. Child Abuse Neglect. (2014) 38:928–38. 10.1016/j.chiabu.2013.12.007 24521524 PMC4065225

[363] Barahmand U Khazaee A Sadeghi Hashjin G. Emotion dysregulation mediates between childhood emotional abuse and motives for substance use. Arch Psychiatr Nurs. (2016) 30:653–9. 10.1016/j.apnu.2016.02.007 27888955

[364] Li D Li D Wu N Wang Z. Intergenerational transmission of emotion regulation through parents' reactions to children's negative emotions: Tests of unique, actor, partner, mediating effects. Child Youth Serv Rev. (2019) 101:113–22. 10.1016/j.childyouth.2019.03.038

[365] Li Z Coles CD Lynch ME Hamann S Peltier S LaConte S . Prenatal cocaine exposure alters emotional arousal regulation and its effects on working memory. Neurotoxicol Teratol. (2009) 31:342–8. 10.1016/j.ntt.2009.08.005 19699795 PMC2761988

[366] Baker JK Fenning RM Moffitt J. A cross-sectional examination of the internalization of emotion co-regulatory support in children with ASD. J Autism Dev Disord. (2019) 49:4332–8. 10.1007/s10803-019-04091-0 31201578 PMC7153849

[367] Brereton AV Tonge BJ Einfeld SL. Psychopathology in children and adolescents with autism compared to young people with intellectual disability. J Autism Dev Disord. (2006) 36:863–70. 10.1007/s10803-006-0125-y 16897401

[368] Lecavalier L Leone S Wiltz J. The impact of behaviour problems on caregiver stress in young people with autism spectrum disorders. J Intellectual Disability Res. (2006) 50:172–83. 10.1111/j.1365-2788.2005.00732.x 16430729

[369] Mazefsky CA White SW. Emotion regulation. Child Adolesc Psychiatr Clin N Am. (2014) 23:15–24. 10.1016/j.chc.2013.07.002 24231164 PMC3830422

[370] Samson AC Hardan AY Podell RW Phillips JM Gross JJ. Emotion regulation in children and adolescents with autism spectrum disorder. Autism Res. (2014) 8:9–18. 10.1002/aur.1387 24863869

[371] Berkovits L Eisenhower A Blacher J. Emotion regulation in young children with autism spectrum disorders. J Autism Dev Disord. (2016) 47:68–79. 10.1007/s10803-016-2922-2 27838805

[372] Jahromi LB Meek SE Ober-Reynolds S. Emotion regulation in the context of frustration in children with high functioning autism and their typical peers. J Child Psychol Psychiatry. (2012) 53:1250–8. 10.1111/j.1469-7610.2012.02560.x 22591180

[373] Jahromi LB Bryce CI Swanson J. The importance of self-regulation for the school and peer engagement of children with high-functioning autism. Res Autism Spectr Disord. (2013) 7:235–46. 10.1016/j.rasd.2012.08.012

[374] Joshi G Wozniak J Fitzgerald M Faraone S Fried R Galdo M . High risk for severe emotional dysregulation in psychiatrically referred youth with autism spectrum disorder: A controlled study. J Autism Dev Disord. (2018) 48:3101–15. 10.1007/s10803-018-3542-9 29675767

[375] Pitskel NB Bolling DZ Kaiser D M Pelphrey KA Crowley MJ. Neural systems for cognitive reappraisal in children and adolescents with autism spectrum disorder. Dev Cogn Neurosci. (2014) 10:117–28. 10.1016/j.dcn.2014.08.007 25198094 PMC4253669

[376] Stark KH Barnes JC Young ND Gabriels RL. Brief report: Understanding crisis behaviors in hospitalized psychiatric patients with autism spectrum disorder-Iceberg Assessment Interview. J Autism Dev Disord. (2015) 45:3468–74. 10.1007/s10803-015-2552-0 26324248

[377] López-Pérez B Ambrona T Gummerum M. Emotional preferences and goals and emotion dysregulation in children with Asperger's syndrome and typically developing children. Br J Clin Psychol. (2018) 57:274–90. 10.1111/bjc.12173 29400401

[378] Gillham JE Carter AS Volkmar FR Sparrow SS. Toward a developmental operational definition of autism. J Autism Dev Disord. (2000) 30:269–78. 10.1023/A:1005571115268 11039854

[379] Samson AC Wells WM Phillips JM Hardan AY Gross JJ. Emotion regulation in autism spectrum disorder: evidence from parent interviews and children's daily diaries. J Child Psychol Psychiatry. (2014) 56:903–13. 10.1111/jcpp.12370 25442191

[380] Gadow KD Pinsonneault JK Perlman G Sadee W. Association of dopamine gene variants, emotion dysregulation and ADHD in autism spectrum disorder. Res Dev Disabil. (2014) 35:1658–65. 10.1016/j.ridd.2014.04.007 24780147 PMC4084560

[381] Rieffe C Camodeca M Pouw LBC Lange AMC Stockmann L. Don't anger me! Bullying, victimization, and emotion dysregulation in young adolescents with ASD. Eur J Dev Psychol. (2012) 9:351–70. 10.1080/17405629.2012.680302

[382] Rieffe C Oosterveld P Meerum Terwogt M Mootz S van Leeuwen E Stockmann L. Emotion regulation and internalizing symptoms in children with autism spectrum disorders. Autism. (2011) 15:655–70. 10.1177/1362361310366571 21733959

[383] Swain D Scarpa A White S Laugeson E. Emotion dysregulation and anxiety in adults with ASD: Does social motivation play a role? J Autism Dev Disord. (2015) 45:3971–7. 10.1007/s10803-015-2567-6 26319254

[384] Vasa RA Kreiser LN Keefer A Singh V Mostofsky SH. Relationships between autism spectrum disorder and intolerance of uncertainty. Autism Res. (2018) 11:636–44. 10.1002/aur.1916 29316350 PMC5903967

[385] Richdale AL Kimberly AK. Schreck. Sleep problems in autism spectrum disorders: Prevalence, nature, and possible biopsychosocial aetiologies. Sleep Med Rev. (2009) 13:403–11. 10.1016/j.smrv.2009.02.003 19398354

[386] Taylor MA Schreck KA Mulick JA. Sleep disruption as a correlate to cognitive and adaptive behavior problems in autism spectrum disorders. Res Dev Disabil. (2012) 33:1408–17. 10.1016/j.ridd.2012.03.013 22522199

[387] Zaidman-Zait A Zwaigenbaum L Duku E Bennett T Szatmari P Mirenda P . Factor analysis of the children's sleep habits questionnaire among preschool children with autism spectrum disorder. Res Dev Disabil. (2020) 97:103548. 10.1016/j.ridd.2019.103548 31901672

[388] Fenning RM Baker JK Moffitt J. Intrinsic and extrinsic predictors of emotion regulation in children with autism spectrum disorder. J Autism Dev Disord. (2018) 48:3858–70. 10.1007/s10803-018-3647-1 29926292 PMC6291376

[389] Simonoff E Jones CRG Pickles A Happé F Baird G Charman T. Severe mood problems in adolescents with autism spectrum disorder. J Child Psychol Psychiatry. (2012) 53:1157–66. 10.1111/j.1469-7610.2012.02600.x 22909395

[390] Uljarevic' M Hedley D Nevill R Evans DW Ying Cai R Butter E . Brief report: Poor self-regulation as a predictor of individual differences in adaptive functioning in young children with autism spectrum disorder. Autism Res. (2018) 11:1157–65. 10.1002/aur.1953 29624924

[391] Mazefsky CA Day TN Siegel M White SW Yu L Pilkonis PA. Development of the emotion dysregulation inventory: A PROMIS®ing method for creating sensitive and unbiased questionnaires for autism spectrum disorder. J Autism Dev Disord. (2016) 48:3736–46. 10.1007/s10803-016-2907-1 27699685 PMC5378674

[392] Mazefsky CA Yu L White SW Siegel M Pilkonis PA. The emotion dysregulation inventory: Psychometric properties and item response theory calibration in an autism spectrum disorder sample. Autism Res. (2018) 11:928–41. 10.1002/aur.1947 29624893 PMC6026073

[393] Jerrell JM McIntyre RS Deroche CB. Diagnostic clusters associated with an early onset schizophrenia diagnosis among children and adolescents. Human Psychopharmacol. (2017) 32:e2589. 10.1002/hup.2589 28370311

[394] Kelleher I Connor D Clarke CM Devlin N Harley M. Prevalence of psychotic symptoms in childhood and adolescence: a systematic review and meta-analysis of population-based studies. Psychol Med. (2012) 42:1857–63. 10.1017/S0033291711002960 22225730

[395] Okkels N Vernal DL Jensen SOW McGrath JJ Nielsen RE. Changes in the diagnosed incidence of early onset schizophrenia over four decades. Acta Psychiatr Scand. (2012) 127:62–8. 10.1111/j.1600-0447.2012.01913.x 22906158

[396] Millier A Schmidt U Angermeyer MC Chauhan D Murthy V Toumi M . Humanistic burden in schizophrenia: A literature review. J Psychiatr Res. (2014) 54:85–93. 10.1016/j.jpsychires.2014.03.021 24795289

[397] Tandon R Gaebel W Barch DM Bustillo J Gur RE Heckers S . Definition and description of schizophrenia in the DSM-5. Schizophr Res. (2013) 150:3–10. 10.1016/j.schres.2013.05.028 23800613

[398] DeLoore E Gunther N Drukker M Feron F Sabbe B Deboutte D . Persistence and outcome of auditory hallucinations in adolescence: A longitudinal general population study of 1800 individuals. Schizophr Res. (2011) 127:252–6. 10.1016/j.schres.2011.01.015 21315559

[399] Morrison AP. The interpretation of intrusions in psychosis: An integrative cognitive approach to hallucinations and delusions. Behav Cogn Psychother. (2001) 29:257–76. 10.1017/S1352465801003010

[400] Scott J Martin G Bor W Sawyer M Clark J McGrath J. The prevalence and correlates of hallucinations in Australian adolescents: Results from a national survey. Schizophr Res. (2009) 107:179–85. 10.1016/j.schres.2008.11.002 19046858

[401] Fonseca-Pedrero E Paino M Lemos-Giráldez S Muñiz J. Schizotypal traits and depressive symptoms in nonclinical adolescents. Compr Psychiatry. (2011) 52:293–300. 10.1016/j.comppsych.2010.07.001 21497224

[402] Fonseca-Pedrero E Lemos-Giráldez S Paino M Muñiz J. Schizotypy, emotional-behavioural problems and personality disorder traits in a non-clinical adolescent population. Psychiatry Res. (2011) 190:316–21. 10.1016/j.psychres.2011.07.007 21802744

[403] Gooding DC Tallent KA Matts CW. Clinical status of at-risk individuals 5 years later: Further validation of the psychometric high-risk strategy. J Abnorm Psychol. (2005) 114:170–5. 10.1037/0021-843X.114.1.170 15709824

[404] Kwapil TR Barrantes-Vidal N Silvia PJ. The dimensional structure of the Wisconsin schizotypy scales: Factor identification and construct validity. Schizophr Bull. (2007) 34:444–57. 10.1093/schbul/sbm098 17768308 PMC2632435

[405] Raine A. Schizotypal personality: Neurodevelopmental and psychosocial trajectories. Annu Rev Clin Psychol. (2006) 2:291–326. 10.1146/annurev.clinpsy.2.022305.095318 17716072

[406] Rideout VJ Foehr UG Roberts DF. Generation m2. Media in the Lives of 8- to 18-Year-Olds. A Kaiser Family Foundation study. (2010). Available online at: https://files.eric.ed.gov/fulltext/ED527859.pdf (accessed September 7, 2020).

[407] Mihara S Higuchi S. Cross-sectional and longitudinal epidemiological studies of Internet Gaming Disorder: A systematic review of the literature. Psychiatry Clin Neurosci. (2017) 71:425–44. 10.1111/pcn.12532 28436212

[408] Amendola S Spensieri V Guidetti V Cerutti R. The relationship between difficulties in emotion regulation and dysfunctional technology use among adolescents. J Psychopathol. (2018) 25:10–7.

[409] Mo PKH Chan VWY Chan SW Lau JTF. The role of social support on emotion dysregulation and internet addiction among Chinese adolescents: A structural equation model. Addict Behav. (2018) 82:86–93. 10.1016/j.addbeh.2018.01.027 29501012

[410] Casale S Caplan SE Fioravanti G. Positive metacognitions about internet use: The mediating role in the relationship between emotional dysregulation and problematic use. Addict Behav. (2016) 59:84–8. 10.1016/j.addbeh.2016.03.014 27077964

[411] Donald JN Ciarrochi J Sahdra BK. The consequences of compulsion: A 4-year longitudinal study of compulsive internet use and emotion regulation difficulties. Emotion. (2020). 10.1037/emo0000769 32551747

[412] Kuss DJ Griffiths MD. Internet gaming addiction: A systematic review of empirical research. Int J Ment Health Addict. (2012) 10:278–96. 10.1007/s11469-011-9318-5

[413] Paulus FW Ohmann S von Gontard A Popow C. Internet gaming disorder in children and adolescents: a systematic review. Dev Med Child Neurol. (2018) 60:645–59. 10.1111/dmcn.13754 29633243

[414] Paulus FW Hübler K Mink F Möhler E. Emotional dysregulation in preschool age predicts later media use and Gaming Disorder symptoms in childhood. Front Psychiatry. (2021) 12:626387. 10.3389/fpsyt.2021.626387 34220565 PMC8245768

[415] Gaetan S Bréjard V Bonnet A. Video games in adolescence and emotional functioning: Emotion regulation, emotion intensity, emotion expression, and alexithymia. Comput Human Behav. (2016) 61:344–9. 10.1016/j.chb.2016.03.027

[416] Hollett KB Harris N. Dimensions of emotion dysregulation associated with problem video gaming. Addiction Res Theory. (2019) 28:38–45. 10.1080/16066359.2019.1579801

[417] Wichstrøm L Stenseng F Belsky J von Soest T Wold Hygen B. Symptoms of Internet Gaming Disorder in youth: Predictors and comorbidity. J Abnorm Child Psychol. (2018) 47:71–83. 10.1007/s10802-018-0422-x 29623484 PMC6329732

[418] Poobalan AS Aucott LS Ross L Cairns W Smith S Helms PJ . Effects of treating postnatal depression on mother-infant interaction and child development. Br J Psychiatry. (2007) 191:378–86. 10.1192/bjp.bp.106.032789 17978316

[419] Furlong M McGilloway S Bywater T Hutchings J Smith SM Donnelly M. Behavioural and cognitive-behavioural group-based parenting programmes for early-onset conduct problems in children aged 3 to 12 years. Cochrane Database Syst Rev. (2012) 15:CD008225. 10.1002/14651858.CD008225.pub2 22336837 PMC12935172

[420] Euser S Alink LRA Stoltenborgh M Bakermans-Kranenburg MJ van IJzendoorn MH. A gloomy picture: a meta-analysis of randomized controlled trials reveals disappointing effectiveness of programs aiming at preventing child maltreatment. BMC Public Health. (2015) 15:2387. 10.1186/s12889-015-2387-9 26476980 PMC4609474

[421] Brown LK Whiteley L Houck CD Craker LK Lowery A Beausoleil N . The role of affect management for HIV risk reduction for youth in alternative schools. J Am Acad Child Adolesc Psychiatry. (2017) 56:524–31. 10.1016/j.jaac.2017.03.010 28545758 PMC5465640

[422] Tolou-Shams R Dauria E Conrad MS Kemp K Johnson S. Outcomes of a family-based HIV prevention intervention for substance using juvenile offenders. J Subst Abuse Treat. (2017) 77:115–25. 10.1016/j.jsat.2017.03.013 28476263 PMC5769453

[423] Webster-Stratton C Jamila Reid M Stoolmiller M. Preventing conduct problems and improving school readiness: evaluation of the incredible years teacher and child training programs in high-risk schools. J Child Psychol Psychiatry. (2008) 49:471–88. 10.1111/j.1469-7610.2007.01861.x 18221346 PMC2735210

[424] Loevaas MES Mari Sund A Lydersen S Neumer SP Martinsen K. Does the transdiagnostic EMOTION intervention improve emotion regulation skills in children? J Child Fam Stud. (2019) 28:805–13. 10.1007/s10826-018-01324-129687457

[425] Martinsen KD Rasmussen LMP Wentzel-Larsen T Holen S Mari Sund A . Prevention of anxiety and depression in school children: Effectiveness of the transdiagnostic EMOTION program. J Consult Clin Psychol. (2019) 87:212–9. 10.1037/ccp0000360 30550301

[426] Deplus S Billieux J Scharff C Philippot P. A mindfulness-based group intervention for enhancing self-regulation of emotion in late childhood and adolescence: A pilot study. Int J Ment Health Addict. (2016) 14:775–90. 10.1007/s11469-015-9627-1

[427] Guendelman S Medeiros S Rampes H. Mindfulness and emotion regulation: insights from neurobiological, psychological, clinical studies. Front Psychol. (2017) 8:220. 10.3389/fpsyg.2017.00220 28321194 PMC5337506

[428] Grégoire C Faymonville ME Vanhaudenhuyse A Jerusalem G Willems S. Randomized controlled trial of a group intervention combining self-hypnosis and self-care: secondary results on self-esteem, emotional distress and regulation, and mindfulness in post treatment cancer patients. Quality of Life Res. (2020) 30:425–36. 10.1007/s11136-020-02655-7 33025372 PMC7886776

[429] Linhartová P Látalová A Kóša B Kašpárek T Schmahl C Paret C. FMRI neurofeedback in emotion regulation: A literature review. Neuroimage. (2019) 193:75–92. 10.1016/j.neuroimage.2019.03.011 30862532

[430] Courtney-Seidler EA Burns K Zilber I Miller AL. Adolescent suicide and self-injury: Deepening the understanding of the biosocial theory and applying dialectical behavior therapy. Int J Behav Consult Therapy. (2014) 9:35–40. 10.1037/h0101638

[431] López-Pinar C Martínez-Sanchís S Carbonell-Vayá E Sánchez-Meca J Fenollar-Cortés J. Efficacy of nonpharmacological treatments on comorbid internalizing symptoms of adults with attention-deficit/hyperactivity disorder: A meta-analytic review. J Atten Disord. (2019) 24:456–78. 10.1177/1087054719855685 31189374

[432] Garrett AS Miklowitz DJ Howe ME Singh MK Acquaye TK Hawkey CG . Changes in brain activation following psychotherapy for youth with mood dysregulation at familial risk for bipolar disorder. Progress Neuro-Psychopharmacol Biol Psychiatry. (2015) 56:215–20. 10.1016/j.pnpbp.2014.09.007 25283342 PMC4258439

[433] Dixius A Möhler E. Feasibility and effectiveness of a new short-term psychotherapy concept for adolescents with emotional dysregulation. Front Psychiatry. (2021) 11:585250. 10.3389/fpsyt.2020.585250 33551862 PMC7858646

[434] Kiani B Hadianfard H Mitchell JT. The impact of mindfulness meditation training on executive functions and emotion dysregulation in an Iranian sample of female adolescents with elevated attention-deficit/hyperactivity disorder symptoms. Aust J Psychol. (2016) 69:273–82. 10.1111/ajpy.12148

[435] Thoder VJ Cautilli JD. An independent evaluation of mode deactivation therapy for juvenile offenders. Int J Behav Consult Therapy. (2011) 7:40–5. 10.1037/h0100925

[436] Apsche JA Bass CK Backlund B. Mediation analysis of mode deactivation therapy, (MDT). Behav Anal Today. (2012) 13:1–10. 10.1037/h0100723

[437] Ducharme P Wharff E Kahn J Hutchinson E Logan G Waber D . Augmenting anger control therapy with a videogame requiring emotional control: A pilot study on an inpatient psychiatric unit. Adolescent Psychiatry. (2012) 2:323–32. 10.2174/2210676611202040323

[438] Bass CK Apsche JA. Mediation analysis of mode deactivation therapy (Reanalysis and interpretation). Int J Behav Consult Therapy. (2013) 8:1–6. 10.1037/h0100967

[439] Ravindran N Engle JM McElwain NL Kramer L. Fostering parents' emotion regulation through a sibling-focused experimental intervention. J Family Psychol. (2015) 29:458–68. 10.1037/fam0000084 26053350

[440] West M Melvin G McNamara F Gordon M. An evaluation of the use and efficacy of a sensory room within an adolescent psychiatric inpatient unit. Aust Occup Ther J. (2017) 64:253–63. 10.1111/1440-1630.12358 28138979

[441] Döpfner M Katzmann J Hanisch C Fegert JM Kölch M Ritschel A . Affective dysregulation in childhood - optimizing prevention and treatment: protocol of three randomized controlled trials in the ADOPT study. BMC Psychiatry. (2019) 19:2239. 10.1186/s12888-019-2239-8 31477086 PMC6720991

[442] Simpson S Wyke S Mercer SW. Adaptation of a mindfulness-based intervention for incarcerated young men: a feasibility study. Mindfulness. (2019) 10:1568–78. 10.1007/s12671-018-1076-z

[443] Evans SC Weisz JR Carvalho AC Garibaldi PM Bearman SK Chorpita BF. Effects of standard and modular psychotherapies in the treatment of youth with severe irritability. J Consult Clin Psychol. (2020) 88:255–68. 10.1037/ccp0000456 32068426

[444] Sesma Pardo E Fernández Rivas A Orgaz Barnier P Beá Mirabent M Kerexeta Lizeaga I Díaz Cosgaya . A qualitative research of adolescents with behavioral problems about their experience in a dialectical behavior therapy skills training group. BMC Psychiatry. (2020) 20:2649. 10.1186/s12888-020-02649-2 32429886 PMC7238612

[445] Winiarski DA Schechter JC Brennan A P Foster SL Cuningham PB. Adolescent physiological and behavioral patterns of emotion dysregulation predict multisystemic therapy response. J Emot Behav Disord. (2016) 25:131–42. 10.1177/1063426616638315 28867925 PMC5580832

[446] Popolo R MacBeth A Brunello S Canfora F Ozdemir E Rebecchi D . Metacognitive interpersonal therapy in group: a feasibility study. Res Psychother. (2018) 21:338. 10.4081/ripppo.2018.338 32913773 PMC7451332

[447] Blader JC Pliszka SR Kafantaris V Sauder C Posner J Foley CA . Prevalence and treatment outcomes of persistent negative mood among children with attention-deficit/hyperactivity disorder and aggressive behavior. J Child Adolesc Psychopharmacol. (2016) 26:164–73. 10.1089/cap.2015.0112 26745211 PMC4800385

[448] Bogen S Legenbauer T Gest S Holtmann M. Morning bright light therapy: a helpful tool for reducing comorbid symptoms of affective and behavioral dysregulation in juvenile depressed inpatients? A pilot trial. Zeitschrift für Kinder- und Jugendpsychiatrie und Psychotherapie. (2017) 45:34–41. 10.1024/1422-4917/a000442 27299514

[449] Ford JD Steinberg KL Hawke J Levine J Zhang W. Randomized trial comparison of emotion regulation and relational psychotherapies for PTSD with girls involved in delinquency. J Clin Child Adolesc Psychol. (2012) 41:27–37. 10.1080/15374416.2012.632343 22233243

[450] Adrian M Zeman J Erdley C Whitlock K Sim L. Trajectories of non- suicidal self-injury in adolescent girls following inpatient hospitalization. Clin Child Psychol Psychiatry. (2019) 24:831–46. 10.1177/1359104519839732 30947520

[451] Goldstein TR Axelson DA Birmaher B Brent DA. Dialectical behavior therapy for adolescents with bipolar disorder: A 1-year open trial. J Am Acad Child Adolesc Psychiatry. (2007) 46:820–30. 10.1097/chi.0b013e31805c1613 17581446 PMC2823290

[452] Heinrich H Gevensleben H Becker A Rothenberger A. Effects of neurofeedback on the dysregulation profile in children with ADHD: SCP NF meets SDQ-DP - a retrospective analysis. Psychol Med. (2019) 50:258–63. 10.1017/S0033291718004130 30674360

[453] Kaufman EA Puzia ME Godfrey DA Crowell SE. Physiological and behavioral effects of interpersonal validation: A multilevel approach to examining a core intervention strategy among self-injuring adolescents and their mothers. J Clin Psychol. (2019) 76:559–80. 10.1002/jclp.22902 31742683

[454] Marco JH García-Palacios A Botella C. Dialectical behavioural therapy for oppositional defiant disorder. Psicothema. (2013) 25:158–63. 10.7334/psicothema2012.119 23628528

[455] Schuppert HM Giesen-Bloo J van Gemert TG Wiersema HM Minderaa RB Emmelkamp PMG . Effectiveness of an emotion regulation group training for adolescents-a randomized controlled pilot study. Clin Psychol Psychotherapy. (2009) 16:467–78. 10.1002/cpp.637 19630069

[456] Sloan E Hall K Simpson A Youssef GJ Moulding R Mildred H . An emotion regulation treatment for young people with complex substance use and mental health issues: A case-series analysis. Cogn Behav Pract. (2018) 25:427–41. 10.1016/j.cbpra.2017.12.006

[457] Suveg C Sood E Comer JS Kendall PC. Changes in emotion regulation following cognitive-behavioral therapy for anxious youth. J Clin Child Adolesc Psychol. (2009) 38:390–401. 10.1080/15374410902851721 19437299

[458] Carthy T Benaroya-Milshtein N Valevski A Apter A. Emotional reactivity and regulation following citalopram therapy in children and adolescents with anxiety disorders. J Child Adolesc Psychopharmacol. (2017) 27:43–51. 10.1089/cap.2015.0067 26771291

[459] Valle Krieger F Ferreira Pheula G Coelho R Zeni T Tramontina S Patrick Zeni C . An open-label trial of risperidone in children and adolescents with severe mood dysregulation. J Child Adolesc Psychopharmacol. (2011) 21:237–43. 10.1089/cap.2010.0123 21663426

[460] Fakra E Khalfa S Da Fonseca D Besnier N Delaveau P Azorin JM . Effect of risperidone versus haloperidol on emotional responding in schizophrenic patients. Psychopharmacology. (2008) 200:261–72. 10.1007/s00213-008-1203-y 18575849

[461] Andrade C Rao SKN. How antidepressant drugs act: A primer on neuroplasticity as the eventual mediator of antidepressant efficacy. Indian J Psychiatry. (2010) 52:378. 10.4103/0019-5545.74318 21267376 PMC3025168

[462] Dougherty DM Olvera RL Acheson A Hill-Kapturczak N Ryan SR Mathias CW. Acute effects of methylphenidate on impulsivity and attentional behavior among adolescents comorbid for ADHD and conduct disorder. J Adolesc. (2016) 53:222–30. 10.1016/j.adolescence.2016.10.013 27816696 PMC5116269

[463] Masi G Milone A Manfredi A Brovedani P Pisano S Muratori P. Combined pharmacotherapy-multimodal psychotherapy in children with disruptive behavior disorders. Psychiatry Res. (2016) 238:8–13. 10.1016/j.psychres.2016.02.010 27086204

[464] Domes G Heinrichs M Gläscher J Büchel C Braus DF Herpertz SC. Oxytocin attenuates amygdala responses to emotional faces regardless of valence. Biol Psychiatry. (2007) 62:1187–90. 10.1016/j.biopsych.2007.03.025 17617382

[465] Zaehringer J Ende G Santangelo P Kleindienst N Ruf M Bertsch K . Improved emotion regulation after neurofeedback: A single-arm trial in patients with Borderline Personality Disorder. NeuroImage: Clin. (2019) 24:102032. 10.1016/j.nicl.2019.102032 31795041 PMC6978219

[466] Quevedo K Yuan Teoh J Engstrom M Wedan R Santana-Gonzalez C Zewde B . Amygdala circuitry during neurofeedback training and symptoms' change in adolescents with varying depression. Front Behav Neurosci. (2020) 14:110. 10.3389/fnbeh.2020.00110 32774244 PMC7388863

[467] Nicholson AA Rabellino D Densmore M Frewen PA Paret C Kluetsch R . The neurobiology of emotion regulation in posttraumatic stress disorder: Amygdala downregulation via real-time fMRI neurofeedback. Hum Brain Mapp. (2016) 38:541–60. 10.1002/hbm.23402 27647695 PMC6866912

[468] Keng SL Siew Ling Lee C Eisenlohr-Moul TA. Effects of brief daily mindfulness practice on affective outcomes and correlates in a high BPD trait sample. Psychiatry Res. (2019) 280:112485. 10.1016/j.psychres.2019.112485 31408773

[469] Wickramasekera I. How does biofeedback reduce clinical symptoms and do memories and beliefs have biological consequences? Toward a model of mind-body healing. Appl Psychophysiol Biofeedback. (1999) 24:91–105. 10.1023/A:1022201710323 10575537

[470] Gibbs A Moor S Frampton C Watkins W. Impact of psychosocial interventions on children with disruptive and emotional disorders treated in a health camp. Austr New Zeal J Psychiatry. (2008) 42:789–99. 10.1080/00048670802277248 18696283

[471] Insel T Cuthbert B Garvey M Heinssen R Kozak M Pine DS . Research Domain Criteria (RDoC): Toward a new classification framework for research on mental disorders. Am J Psychiatry. (2010) 167:748–51. 10.1176/appi.ajp.2010.09091379 20595427

[472] Fernandez CK Jazaieri H Gross JJ. Emotion regulation: a transdiagnostic perspective on a new RDoC domain. Cognit Ther Res. (2016) 40:426–40. 10.1007/s10608-016-9772-2 27524846 PMC4979607

[473] Ford JD Hawke J. Trauma affect regulation psychoeducation group and milieu intervention outcomes in juvenile detention facilities. J Aggress Maltreat Trauma. (2012) 21:365–84. 10.1080/10926771.2012.673538

[474] Marrow M Knudsen K Olafson E Bucher S. The value of implementing TARGET within a trauma-informed juvenile justice setting. J Child Adolesc Trauma. (2012) 5:257–70. 10.1080/19361521.2012.697105

