# Supplementary material for: Emotional Dysregulation in Children and Adolescents With Psychiatric Disorders. A Narrative Review
Source: Front Psychiatry. 2021 Oct 25;12:628252. doi: 10.3389/fpsyt.2021.628252 (PMC8573252; doi:10.3389/fpsyt.2021.628252)
Supplement: Supplementary file 1 [file Data_Sheet_1.docx]

Appendix:

References used for the different psychiatric disorders

**3.1 ADHD**

**31.** American Psychiatric Association. (2013). Diagnostic and Statistical Manual of Mental Disorders (DSM-5®), Fifth Edition. Washington, DC: American Psychiatric Association.

**32.** WHO. (2019). ICD-11. https://icd.who.int/browse11/l-m/en. [Accessed October 25, 2020] **33.** Wozniak, J., Biederman, J., Kiely, K., Ablon, S., Faraone, S. V., and Mundy, E., et al. (1995). Mania-like symptoms suggestive of childhood-onset bipolar disorder in clinically referred children. Journal of the American Academy of Child and Adolescent Psychiatry, 34(7), 867–876. <https://doi.org/10.1891/1946-6560.9.1.18>

**34.** Vacher, C., Goujon, A., Romo, L., and Purper-Ouakil, D. (2020). Efficacy of psychosocial interventions for children with ADHD and emotion dysregulation: A systematic review. Psychiatry Research, 291, 113151. <https://doi.org/10.1016/j.psychres.2020.113151>

**35.** Bottelier, M., A., Schrantee, A., Ferguson, B., Tamminga, H., G., H., Bouziane, C., and Kooij, J. J. S., et al. (2017). Age-dependent effects of acute methylphenidate on amygdala reactivity in stimulant treatment-naive patients with attention deficit/hyperactivity disorder. Psychiatry Research: Neuroimaging, 269, 36–42. <https://doi.org/10.1016/j.pscychresns.2017.09.009>

**36**. McQuade J. D., and Breaux, R. P. (2016). Are elevations in ADHD symptoms associated with physiological reactivity and emotion dysregulation in children? Journal of Abnormal Child Psychology, 45(6), 1091–1103. <https://doi.org/10.1007/s10802-016-0227-8>

**37**. Qian, Y., Chang, W., He, X., Yang, L., Liu, L., Ma, Q., et al. (2016). Emotional dysregulation of ADHD in childhood predicts poor early-adulthood outcomes: A prospective follow up study. Research in Developmental Disabilities, 59, 428–436. <https://doi.org/10.1016/j.ridd.2016.09.022>

**38**. Bunford, N., Evans, S. W., and Langberg, J. M. (2018). Emotion dysregulation is associated with social impairment among young adolescents with ADHD. Journal of Attention Disorders, 22(1), 66–82. <https://doi.org/10.1177/1087054714527793>

**39**. Gamli, I. S., and Tahiroglu, A. Y. (2018). Six months methylphenidate treatment improves emotion dysregulation in adolescents with attention deficit/hyperactivity disorder: A prospective study. Neuropsychiatric Disease and Treatment, 14, 1329–1337. <https://doi.org/10.2147/NDT.S164807>

**40**. Wheeler Maegden J., and Carlson, C. L. (2000). Social functioning and emotional regulation in the attention deficit hyperactivity disorder. Journal of Clinical Child Psychology, 29(1), 30–42. <https://doi.org/10.1207/S15374424jccp2901_4>

**41**. Huguet, A., Izaguirre Eguren, J., Miguel-Ruiz, D., Vall Vallés, X., and Alda, J. A. (2019).

Deficient emotional self-regulation in children with attention deficit hyperactivity disorder. Journal of Developmental & Behavioral Pediatrics, 40(6), 425–431. <https://doi.org/10.1097/dbp.0000000000000682>

**42**. Groves, N. B., Kofler, M. J., Wells, E. L., Day, T. N., and Chan, E. S. M. (2020). An examination of relations among working memory, ADHD symptoms, and emotion regulation. Journal of Abnormal Child Psychology, 48(4), 525–537. <https://doi.org/10.1007/s10802-019-00612-8>

**43**. Rosen P. J., and Factor, P. I. (2015). Emotional impulsivity and emotional and behavioral difficulties among children with ADHD. Journal of Attention Disorders, 19(9), 779–793. <https://doi.org/10.1177/1087054712463064>

**44**. Sørensen, L., Plessen, K. J., Nicholas, J., and Lundervold, A. J. Is behavioral regulation in children with ADHD aggravated by comorbid anxiety disorder? Journal of Attention Disorders, 15(1):56–66, 2010. <https://doi.org/10.1177/1087054709356931>

**45**. Özbaran, B., Kalyoncu, T., and Köse, S. (2018). Theory of mind and emotion regulation difficulties in children with ADHD. Psychiatry Research, 270, 117–122. <https://doi.org/10.1016/j.psychres.2018.09.034>

**46**. Romvig Overgaard, K., Oerbeck, B., Aase, H., Torgersen, S., Reichborn-Kjennerud, T., and Zeiner, P. (2015). Emotional lability in preschoolers with symptoms of ADHD. Journal of Attention Disorders, 22(8), 787–795. <https://doi.org/10.1177/1087054715576342>

**47**. Lugo-Candelas, C., Flegenheimer, C., McDermott, J. M. and Harvey, E. (2016). Emotional understanding, reactivity, and regulation in young children with ADHD symptoms. Journal of Abnormal Child Psychology, 45(7), 1297–1310. <https://doi.org/10.1007/s10802-016-0244-7>

**48**. Cremone, A., Lugo-Candelas, C. I., Harvey, E. A. Jennifer M. McDermott, and

Rebecca M. C. Spencer, R. M. C. (2018). Positive emotional attention bias in young children with symptoms of ADHD. Child Neuropsychology, 24(8), 1137–1145. <https://doi.org/10.1080/09297049.2018.1426743>

**49**. Tenenbaum, R. B.,Musser, E. D., Morris, S.,Ward, A. R., Raiker, J. S., and Coles, E. K., et al. (2018). Pelham. Response inhibition, response execution, and emotion regulation among children with attention-deficit/hyperactivity disorder. Journal of Abnormal Child Psychology, 47(4), 589–603. <https://doi.org/10.1007/s10802-018-0466-y>

**50**. López-Martín, S., Albert, J., Fernández-Jaén, A., and Carretié, L. (2015), Emotional response inhibition in children with attention-deficit/hyperactivity disorder: neural and behavioural data. Psychological Medicine, 45(10), 2057–2071. <https://doi.org/10.1017/S0033291714003195>

**51**. Seymour, K. E., Tang, X., Crocetti, D., Mostofsky, S. H., Miller, M. I., and Rosch, K. S. Anomalous subcortical morphology in boys, but not girls, with ADHD compared to

typically developing controls and correlates with emotion dysregulation. Psychiatry Research: Neuroimaging, 261:20–28, 2017. <https://doi.org/10.1016/j.pscychresns.2017.01.002>

**52**. Bunford, N., Evans, S. W., and Wymbs, F. (2015). ADHD and emotion dysregulation among children and adolescents. Clinical Child and Family Psychology Review, 18(3), 185– 217. <https://doi.org/10.1007/s10567-015-0187-5>

**53.** Lee, C. A., Milich, R., Lorch, E. P., Flory, K., Sarno Owens, J., and Lamont, A. E. (2017). Forming first impressions of children: the role of attentiondeficit/hyperactivity disorder symptoms and emotion dysregulation. Journal of Child Psychology and Psychiatry, 59(5), 556–564. <https://doi.org/10.1111/jcpp.12835>

**54**. Williams, K. E., and Sciberras, E. (2016). Sleep and self-regulation from birth to 7 years. Journal of Developmental & Behavioral Pediatrics, 37(5), 385–394. <https://doi.org/10.1097/dbp.0000000000000281>

**55**. Kutlu, A., Akyol Ardic, U., and Sabri Ercan, E. (2017). Effect of methylphenidate on emotional dysregulation in children with attention-deficit/hyperactivity disorder and oppositional defiant disorder/conduct disorder. Journal of Clinical Psychopharmacology, 37(2), 220–225. <https://doi.org/10.1097/jcp.0000000000000668>

**56**. Winters, D. E., Fukui, S., Leibenluft, E., and Hulvershorn, L. A. (2018). Improvements in irritability with open-label methylphenidate treatment in youth with comorbid attention deficit/hyperactivity disorder and disruptive mood dysregulation disorder. Journal of Child and Adolescent Psychopharmacology, 28(5), 298–305. <https://doi.org/10.1089/cap.2017.0124>

**3.1.2 Mood Disorders**

**15**. Guyer, A. E., McClure, E. B., Adler, A. D., Brotman, M. A., Rich, B. A., and Kimes, A. S. et al., (2007). Specificity of facial expression labeling deficits in childhood psychopathology. Journal of Child Psychology and Psychiatry, 48(9):863–871. <https://doi.org/10.1111/j.1469-7610.2007.01758.x>

**21**. Loevaas, M. E. S., Sund, A. M., Patras, J., Martinsen, K., Hjemdal, O., and Neumer S.- P., et al. (2018). Emotion regulation and its relation to symptoms of anxiety and depression in children aged 8–12 years: does parental gender play a differentiating role? BMC Psychology, 6 (1). <https://doi.org/10.1186/s40359-018-0255-y>

**31**. American Psychiatric Association. (2013). Diagnostic and Statistical Manual of Mental Disorders (DSM-5®), Fifth Edition. Washington, DC: American Psychiatric Association.

**32**. WHO. (2019). ICD-11. https://icd.who.int/browse11/l-m/en. [Accessed October 25, 2020] **57.** Walter, H., von Kalckreuth, A., Schardt, D., Stephan, A., Goschke, T., and Erk, S. (2009). The temporal dynamics of voluntary emotion regulation. PLoS ONE, 4(8), e6726. <https://doi.org/10.1371/journal.pone.0006726>

**58**. Young, K. D., Siegle, G. J., Misaki, M., Zotev, V., Phillips, R., Drevets, W. C., et al. (2018). Altered task-based and resting-state amygdala functional connectivity following real- time fMRI amygdala neurofeedback training in major depressive disorder. NeuroImage: Clinical, 17, 691–703. <https://doi.org/10.1016/j.nicl.2017.12.004>

**59.** Folk, J. B., Zeman, J. L., Poon, J. A., and Dallaire, D. H. (2014). A longitudinal examination of emotion regulation: pathways to anxiety and depressive symptoms in urban minority youth. Child and Adolescent Mental Health, 19(4), 243–250. <https://doi.org/10.1111/camh.12058>

**60**. Tahmouresi, N., Bender, C., Schmitz, J., Baleshzar, A., and Tuschen-Caffier, B. (2014). Similarities and differences in emotion regulation and psychopathology in Iranian and German school-children: A cross-cultural study. International Journal of Preventive Medicine, 5, 52–60.

**61.** Feng, X., Keenan, K., Hipwell, A. E., Henneberger, A. K., Rischall, M. S., Butch, J., et al. (2009). Longitudinal associations between emotion regulation and depression in preadolescent girls: Moderation by the caregiving environment. Developmental Psychology, 45(3), 798–808. <https://doi.org/10.1037/a0014617>

**62**. Keenan, K., and Hipwell, A. E. (2005). Preadolescent clues to understanding depression in girls. Clinical Child and Family Psychology Review, 8(2), 89–105. <https://doi.org/10.1016/j.eurpsy.2016.01.762>

**63.** Mirsu-Paun, A. (2016). Grief cognitions and cognitive-emotional regulation associated with romantic breakup distress among college students. European Psychiatry, 33(S1), s284– s284. <https://doi.org/10.1016/j.eurpsy.2016.01.762>

**64.** Stegge, H., and Meerum Terwogt, M. (2007). “Awareness and regulationof emotion in typical and atypical development” in Handbook of Emotion Regulation, ed. J. J. Gross (Guilford Press, New York, NY), 269–286.

**65.** Fussner, L. M., Luebbe, A. M., Mancini, K. J., and Becker, S. P. (2016). Emotion dysregulation mediates the longitudinal relation between peer rejection and depression. International Journal of Behavioral Development, 42(2), 155–166. <https://doi.org10.1177/0165025416669062>

**66.** Fulford, D., Eisner, L. R., and Johnson, S. L. (2015). Differentiating risk for mania and borderline personality disorder: The nature of goal regulation and impulsivity. Psychiatry Research, 227(2-3), 347–352. <https://doi.org/10.1016/j.psychres.2015.02.001>

**67.** Kafantaris, V., Kingsley, P., Ardekani, B., Saito, E., Lencz, T., Lim, K., and Szeszko, P. (2009). Lower orbital frontal white matter integrity in adolescents with bipolar I disorder. Journal of the American Academy of Child & Adolescent Psychiatry, 48(1), 79–86. <https://doi.org/10.1097/chi.0b013e3181900421>

**68.** Chang, K., D., Wagner, C., Garrett, A., Howe, M., and Allan Reiss. (2008). A preliminary functional magnetic resonance imaging study of prefrontal-amygdalaractivation changes in adolescents with bipolar depression treated with lamotrigine. Bipolar Disorders, 10, 426–431. <https://doi.org/10.1111/j.1399-5618.2007.00576.x>

**69.** Roberts, G., Lord, A., Frankland, A., Wright, A., Lau, P., Levy, F., et al. (2017). Functional dysconnection of the inferior frontal gyrus in young people with bipolar disorder or at genetic high risk. Biological Psychiatry, 81(8), 718–727. <https://doi.org/10.1016/j.biopsych.2016.08.018>

**70.** Legenbauer, T., Heiler, S., Holtmann, M., Fricke-Oerkermann, L., and Lehmkuhl, G. (2012). The affective storms of school children during night time: Do affective dysregulated school children show a specific pattern of sleep disturbances? Journal of Neural Transmission, 119(9), 989–998. <https://doi.org/10.1007/s00702-012-0837-4>

**71.** Mehl, R. C., O'Brien, L. M., Jones, J. H., Dreisbach, J. K., Mervis, C. B., and Gozal, D. (2006). Correlates of sleep and pediatric bipolar disorder. Sleep, 29(2), 193–197. <https://doi.org/10.1093/sleep/29.2.193>

**72**. Barch, D. M., Harms, M. P., Tillman, R., Hawkey, E., and Luby, J. L. (2019). Early childhood depression, emotion regulation, episodic memory, and hippocampal development. Journal of Abnormal Psychology, 128(1), 81–95. <https://doi.org/10.1037/abn0000392>

**73**. Kim, P., Arizpe, J., Rosen, B., Razdan, V., Catherine Haring, C., Sarah Jenkins, S., et al. (2013). Impaired fixation to eyes during facial emotion labelling in children with bipolar disorder or severe mood dysregulation. Journal of Psychiatry & Neuroscience, 38(6), 407–416. <https://doi.org/10.1503/jpn.120232>

**74**. Deveney, C. M., Connolly, M., E., Haring, C., T., Bones, B. L., Reynolds, R. C., et al. Neural mechanisms of frustration in chronically irritable children. American Journal of Psychiatry, 170:1186–1194, 2013. <https://doi.org/10.1016/j.biopsycho.2011.10.003>

**75**. Melissa A. Brotman, M. A., Layla Kassem, L., Michelle M. Reising, M. M., Amanda E. Guyer, A. E., Daniel P. Dickstein, D.P., Brendan A., and Rich, B. A. (2007). Parental diagnoses in youth with narrow phenotype bipolar disorder or severe mood dysregulation. American Journal of Psychiatry, 164(8), 1208–1241. <https://doi.org/10.1176/appi.ajp.2007.06101619>

**76.** Schenkel, L. S., Pavuluri, M. N., Herbener, E. S., Harral, E. M. and Sweeney, J. A. (2007). Facial emotion processing in acutely ill and euthymic patients with pediatric bipolar disorder. Journal of the American Academy of Child & Adolescent Psychiatry, 46(8), 1070– 1079. <https://doi.org/10.1097/chi.0b013e3180600fd6>

**77**. Perugi, G., Hantouche, E., and Vannucchi, G. (2017). Diagnosis and Treatment of Cyclothymia: The “Primacy” of Temperament. Current Neuropharmacology, 15 (3), 372- 379. <https://doi.org/10.2174/1570159X14666160616120157>

**78**. Kochman, F.J., Hantouche, E.G., Ferrari, P., Lancrenon, S., Bayart, D., and Akiskal, H.S. (2005). Cyclothymic temperament as a prospective predictor of bipolarity and suicidality in children and adolescents with major depressive disorder. Journal of Affective Disorders, 85 (1-2), 181-189. <https://doi.org/10.1016/j.jad.2003.09.009>

**79**. Akiskal, H.S. (1995). Developmental Pathways to Bipolarity: Are Juvenile-Onset Depressions Pre-Bipolar? Journal of the American Academy of Child & Adolescent Psychiatry, 34 (6), 754-763. <https://doi.org/10.1097/00004583-199506000-00016>

**80**. Signoretta, S., Maremmani, I., Liguori, A., Perugi, G., and Akiskal, H.S. (2005). Affective temperament traits measured by TEMPS-I and emotional-behavioral problems in clinically-well children, adolescents, and young adults. Journal of Affective Disorders, 85 (1- 2), 169-180. <https://doi.org/10.1016/S0165-0327(03)00100-9>

**81**. M. Linehan. (1993) Cognitive-behavioral treatment for borderline personality disorder. New York, NY: Guilford Press.

**82.** Crowell, S. E., Beauchaine, T. P., McCauley, E., Smith, C. J., Stevens, A. L., and Sylvers, P. (2005). Psychological, autonomic, and serotonergic correlates of parasuicide among adolescent girls. Development and Psychopathology, 17(04), 1105–1127. <https://doi.org/10.1017/s0954579405050522>

**83**. Yen, S., Weinstock, L. M., Andover, M. S., Sheets, E. S., Selby, E. A., and Spirito, A. (2012). Prospective predictors of adolescent suicidality: 6-month post-hospitalization follow-up. Psychological Medicine, 43(5), 983–993. <https://doi.org/10.1017/s0033291712001912>

**84.** Bowen, R., Rahman, H., Yue Dong, L., Khalaj, S., Baetz, M., Peters, E., and Balbuena, L. (2019). Suicidality in people with obsessive-compulsive symptoms or personality traits. Frontiers in Psychiatry, 9. <https://doi.org/10.3389/fpsyt.2018.00747>

**85.** Weinberg, A., and Klonsky, E.D. (2009). Measurement of emotion dysregulation in adolescents. Psychological Assessment, 21(4), 616–621. <https://doi.org/10.1037/a0016669>

**3.1.3. Psychological Trauma**

**2.** Gratz, K. L., and Roemer, L. (2004). Multidimensional Assessment of Emotion Regulation and Dysregulation: Development, Factor Structure, and Initial Validation of the Difficulties in Emotion Regulation Scale. Journal of Psychopathology and Behavioral Assessment, 26, 41–54. <https://doi.org/10.1023/B:JOBA.0000007455.08539.94>

**28**. Pat-Horenczyk, R., Cohen, S., Ziv, Y., Achituv, M., Asulin-Peretz, L., Blanchard, T. R., et al. (2015). Emotion regulation in mothers and young children faced with trauma. Infant Mental Health Journal, 36(3), 337–348. <https://doi.org/10.1002/imhj.21515>

**86**. Cloitre, M., Stolbach, B. C., Herman, J. L., van der Kolk, B., Pynoos, R. et al. (2009). A developmental approach to complex PTSD: Childhood and adult cumulative trauma as predictors of symptom complexity. Journal of Traumatic Stress, 22(5), 399–408. <https://doi.org/10.1002/jts.20444>

**87.** Young Choi, J., and Ja Oh., K. (2014). Cumulative childhood trauma and psychological maladjustment of sexually abused children in Korea: Mediating effects of emotion regulation. Child Abuse & Neglect, 38(2), 296–303. <https://doi.org/10.1016/j.chiabu.2013.09.009>

**88.** Dvir, Y., Ford, J. D., Hill, M., and Frazier, J. A. (2014). Childhood maltreatment, emotional dysregulation, and psychiatric comorbidities. Harvard Review of Psychiatry, 22(3), 149–161. https://doi.org/ 10.1097/hrp.0000000000000014

**89.** van der Kolk, B. A. (2005). Developmental trauma disorder: Toward a rational diagnosis for children with complex trauma histories. Psychiatric Annals, 35(5), 401–408. <https://doi.org/10.3928/00485713-20050501-06>

**90.** McLaughlin, K.A., Hatzenbuehler, M.L., Mennin, D.S., and Nolen-Hoeksema, S. Emotion dysregulation and adolescent psychopathology: A prospective study. Behaviour Research and Therapy, 49(9):544–554, 2011. <https://doi.org/10.1016/j.brat.2011.06.003>

**91.** Rizeq, J., and McCann, D. (2019). Trauma and affective forecasting: The mediating effect of emotion dysregulation on predictions of negative affect. Personality and Individual Differences, 147, 172–176. <https://doi.org/10.1016/j.paid.2019.04.036>

**92.** Thornback, K., and Muller, R. T. (2015). Relationships among emotion regulation and symptoms during trauma-focused CBT for school-aged children. Child Abuse & Neglect, 50, 182–192. <https://doi.org/10.1016/j.chiabu.2015.09.011>

**93.** Spinazozola, J., van der Kolk, B., and Ford, J.D. (2018). When nowhere is safe: Interpersonal Trauma and attachment adversity as antecedents of Posttraumatic Stress Disorder and Developmental Trauma Disorder. Journal of Traumatic Stress, 31 (5), 631- 642. <https://doi.org/10.1002/jts.22320>

**94.** Ford, J. D., Spinazzola, J., van der Kolk, B., and Grasso, D. (2018). Toward an empirically-based Developmental Trauma Disorder diagnosis for children: Factor structure, item characteristics, reliability and validity of the Developmental Trauma Disorder Semi Structured Interview (DTD-SI). Journal of Clinical Psychiatry, 79(5), e1-e9. <https://doi.org/doi.org/10.4088/JCP.17m11675>

**95.** Powers, A., Stevens, J. S., O'Banion, D., Stenson, A. F., Kaslow, N., Jovanovic, T., et al. (2020). Intergenerational transmission of risk for PTSD symptoms in African American children: The roles of maternal and child emotion dysregulation. Psychological Trauma: Theory, Research, Practice, and Policy. <https://doi.org/10.1037/tra0000543>

**96.** Kim, J., and Cicchetti, D. (2009). Longitudinal pathways linking child maltreatment, emotion regulation, peer relations, and psychopathology. Journal of Child Psychology and Psychiatry, 51(6):706–716, 2009. <https://doi.org/10.1111/j.1469-7610.2009.02202.x>

**97.** Larsen, R. J., and Diener, E. (1987). Affect intensity as an individual difference characteristic: A review. Journal of Research in Personality, 21(1), 1–39. https://doi.org/10.1016/0092-6566(87)90023-7

**98.** Conley, R. R., Ascher-Svanum, H., Zhu, B., Faries, D., and Kinon, B. J. (2007). The burden of depressive symptoms in the long-term treatment of patients with schizophrenia. Schizophrenia Research, 90(1-3), 186–197. <https://doi.org/10.1016/j.schres.2006.09.027>

**99.** Samson, A. C., Huber, O., and Gross, J. J. (2012). Emotion regulation in Asperger's syndrome and high-functioning autism. Emotion, 12(4), 659–665. <https://doi.org/10.1037/a0027975>

**100.** Shields, A., and Cicchetti, D. (2001). Parental maltreatment and emotion dysregulation as risk factors for bullying and victimization in middle childhood. Journal of Clinical Child Psychology, 30(3), 349–363. <https://doi.org/10.1207/S15374424JCCP3003_7>

**101.** Sharma-Patel, K., and Brown, E. J. (2016). Emotion regulation and self blame as mediators and moderators of trauma-specific treatment. Psychology of Violence, 6(3), 400– 409. <https://doi.org/10.1037/vio0000044>

**102.** Lehmann, S., Breivik, K., Monette, S., and Minnis. H. (2020). Potentially traumatic events in foster youth, and association with DSM-5 trauma- and stressor related symptoms. Child Abuse & Neglect, 101:104374. <https://doi.org/10.1016/j.chiabu.2020.104374>

**103.** Langeland, W., and Dijkstra, S. (1995). Breaking the intergenerational transmission of child abuse: Beyond the mother-child relationship. Child Abuse Review, 4(1):4–13. <https://doi.org/10.1002/car.2380040104>

**104.** Kessler, R. C., Petukhova, M., Sampson, N.A., Zaslavsky, A. M., and Wittchen, H-U. (2012) Twelve-month and lifetime prevalence and lifetime morbid risk of anxiety and mood disorders in the United States. International Journal of Methods in Psychiatric Research, 21(3):169–184. <https://doi.org/10.1002/mpr.1359>

**105**. Greif Green, J., McLaughlin, K. A., Berglund, P. A., Gruber, M. J., Sampson, N. A., Zaslavsky, A. M., and Kessler, R. C. (2010). Childhood adversities and adult psychiatric disorders in the National Comorbidity Survey Replication I. Archives of General Psychiatry, 67(2), 113–123. <https://doi.org/10.1001/archgenpsychiatry.2009.186>

**106**. Nusslock, R., and Miller, G. E. (2016). Early-life adversity and physical and emotional health across the lifespan: A neuroimmune network hypothesis. Biological Psychiatry, 80(1), 23–32. <https://doi.org/10.1016/j.biopsych.2015.05.017>

**107**. Smith, A. L., Cross, D., Winkler, J., Jovanovic, T., and Bradley, B. (2014). Emotional dysregulation and negative affect mediate the relationship between maternal history of child maltreatment and maternal child abuse potential. Journal of Family Violence, 29(5), 483– 494. <https://doi.org/10.1007/s10896-014-9606-5>

**108**. Warmingham, J. M., Handley, E. D., Rogosch, F. A., Manly, J. T., and Cicchetti, D. (2019). Identifying maltreatment subgroups with patterns of maltreatment subtype and chronicity: A latent class analysis approach. Child Abuse & Neglect, 87, 28–39. <https://doi.org/10.1016/j.chiabu.2018.08.013>

**109**. Briere, J. (1997). Treating adults severely abused as children: The self-trauma model. In, Child abuse: New directions in treatment and prevention across the lifespan, ed. D. A. Wolfe, B. McMahon, and R. D. Peters. Sage Publications, Newbury Park, CA.

**110**. Briere, J. (2002). Treating adult survivors of severe childhood abuse and neglect: Further development of an integrative model. In The APSAC Handbook on Child Maltreatment ed. J. E. B. Myers, L. Berliner, J. Briere, T. Reid, and C. Jenny. Sage Publications, Newbury Park, CA, 2002.

**111**. J. J. Freyd, J. J. (1996). Betrayal trauma: The logic of forgetting childhood abuse. Cambridge: Havard University Press.

**112**. Sevecke, K., Franke, S., Kosson, D., and Krischer, M. (2016). Emotional dysregulation and trauma predicting psychopathy dimensions in female and male juvenile offenders. Child and Adolescent Psychiatry and Mental Health, 10(1). [https://doi.org/10.1186/s13034-016-0130- 7](https://doi.org/10.1186/s13034-016-0130-%207)

**113**. Herts, K. L., McLaughlin, K. A., and Hatzenbuehler, M. L. (2012). Emotion dysregulation as a mechanism linking stress exposure to adolescent aggressive behavior. Journal of Abnormal Child Psychology, 40(7), 1111–1122. [https://doi.org/10.1007/s10802-012- 9629-4](https://doi.org/10.1007/s10802-012-%209629-4)

**114**. McLaughlin, K. A., Hatzenbuehler, M. L., and Hilt, L. M. (2009). Emotion dysregulation as a mechanism linking peer victimization to internalizing symptoms in adolescents. Journal of Consulting and Clinical Psychology, 77(5), 894–904. <https://doi.org/10.1037/a0015760>

**115**. Vitaro, F., Brendgen, M., and Trembley, R. E. (2002). Reactively and proactively aggressive children: antecedent and subsequent characteristics. Journal of Child Psychology and Psychiatry, 43, 495–506. <https://doi.org/10.1111/1469-7610.00040>

**116**. Jacoby, V. M., Krackow, E., and Scotti, J. R. (2016). Betrayal trauma in youth and negative communication during a stressful task. The International Journal of Aging and Human Development, 84(3), 247–275. <https://doi.org/10.1177/0091415016669724>

**117**. Maughan, A., and Cicchetti, D. (2002). Impact of child maltreatment and interadult violence on children’s emotion regulation abilities and socioemotional adjustment. Child Development, 73(5), 1525–1542. <https://doi.org/10.1111/1467-8624.00488>

**118**. Nederlof , E., Van der Ham, J. M., Dingemans, P. M. J. A., and Oei, T. I. (2010). The relation between dimensions of normal and pathological personality and childhood maltreatment in incarcerated boys. Journal of Personality Disorders, 24(6), 746–762. <https://doi.org/10.1521/pedi.2010.24.6.746>

**119**. Meredith A. Gruhn, M. A., and Compas, B. E. (2020). Effects of maltreatment on coping and emotion regulation in childhood and adolescence: A meta-analytic review. Child Abuse & Neglect, 103:104446. <https://doi.org/10.1016/j.chiabu.2020.104446>

**120**. Vettese, L. C., Dyer, C. E., Ling Li, W., and Wekerle, C. (2011). Does self-compassion mitigate the association between childhood maltreatment and later emotion regulation difficulties? A preliminary investigation. International Journal of Mental Health and Addiction, 9(5), 480–491. <https://doi.org/10.1007/s11469-011-9340-7>

**121**. Krain Roy, A., Lopes, V., and Klein, R. G. (2014). Disruptive mood dysregulation disorder: A new diagnostic approach to chronic irritability in youth. American Journal of Psychiatry, 171(9), 918–924. <https://doi.org/10.1176/appi.ajp.2014.13101301>

**122**. Heleniak, C., King, K. M., Monahan, K. C., and McLaughlin, K. A. (2017). Disruptions in emotion regulation as a mechanism linking community violence exposure to adolescent internalizing problems. Journal of Research on Adolescence, 28(1), 229–244. <https://doi.org/10.1111/jora.12328>

**123**. Miller, D. J., Vachon, D. D., and Aalsma. M. C. (2012). Negative affect and emotion dysregulation. Criminal Justice and Behavior, 39(10), 1316–1327. <https://doi.org/10.1177/0093854812448784>

**124**. Schelble, J. L., Franks, B. A., and Miller, M. D. (2010). Emotion dysregulation and academic resilience in maltreated children. Child & Youth Care Forum, 39(4), 289–303. https://doi.org/10.1007/s10566-010-9105-7

**125**. Bielas, H., Barra, S., Skrivanek, C., Aebi, M., Steinhausen, H-C., Bessler, C., and Plattner, B. The associations of cumulative adverse childhood experiences and irritability with mental disorders in detained male adolescent offenders. Child and Adolescent Psychiatry and Mental Health, 10(1), 2016. <https://doi.org/10.1186/s13034-016-0122-7>

**126**. Heleniak, C., Jenness, J. L., Vander Stoep, A., McCauley, E., and McLaughlin, K.A. (2015) Childhood maltreatment exposure and disruptions in emotion regulation: A transdiagnostic pathway to adolescent internalizing and externalizing psychopathology. Cognitive Therapy and Research, 40(3), 394–415. <https://doi.org/10.1007/s10608-015-9735-z>

**127**. Sachs-Ericsson, N. J., Sheffler, J. L., Stanley, I. H., Piazza, J. R., and Preacher, K. J. (2017). When emotional pain becomes physical: Adverse childhood experiences, pain, and the role of mood and anxiety disorders. Journal of Clinical Psychology, 73(10), 1403–1428. <https://doi.org/10.1002/jclp.22444>

**128**. Xu Peh, C., Shahwan, S., Fauziana, R., Mahesh, M. V., Sambasivam, R., Zhang, Y. (2017). Emotion dysregulation as a mechanism linking child maltreatment exposure and self- harm behaviors in adolescents. Child Abuse & Neglect, 67:383–390. <https://doi.org/10.1016/j.chiabu.2017.03.013>

**129**. Berman, A. K., and Knight, R. A. (2014). The relation of familiarity with sexual abusers to subsequent developmental adaptation in youths who have sexually offended. Sexual Abuse: A Journal of Research and Treatment, 27(6), 587–608. <https://doi.org/10.1177/1079063214544329>

**130**. Kroupina, M. G., Fuglestad, A J., Iverson, S. L., Himes, J. H. Mason, P. W., and Gunnar, M. R. (2012). Adoption as an intervention for institutionally reared children: HPA functioning and developmental status. Infant Behavior and Development, 35(4), 829–837. <https://doi.org/10.1016/j.infbeh.2012.07.011>

**131**. Amelia Arria, Kevin O'Grady, Kimberly Caldeira, Kathryn Vincent, Holly Wilcox, and Eric Wish. Suicide ideation among college students: A multivariate analysis. Archives of Suicide Research, 13(3):230–246, 2009. <https://doi.org/10.1080/13811110903044351>

**132**. Freyd, J. J. (1994). Betrayal trauma: Traumatic amnesia as an adaptive response to childhood abuse. Ethics & Behavior, 4(4), 307–329. <https://doi.org/10.1207/s15327019eb0404_1>

**133.** Schwartz, D., and Proctor, L. J. (2000) Community violence exposure and children's social adjustment in the school peer group: The mediating roles of emotion regulation and social cognition. Journal of Consulting and Clinical Psychology, 68(4), 670–683. <https://doi.org/10.1037/0022-006x.68.4.670>

**134**. Langer Zarling, A., Taber-Thomas, S., Murray. A., Knuston, J.F., Lawrence, E., Valles, N.-L., DeGarmo, D.S. and Bank, L. Internalizing and externalizing symptoms in young children exposed to intimate partner violence: Examining intervening processes. Journal of Family Psychology, 27(6):945–955, 2013. <https://doi.org/10.1037/a0034804>

**135**. Kaszynski, K., Kallis, D. L., Karnik, N., Soller, M., Hunter, S., Haapanen, R, et al. (2014). Incarcerated youth with personality disorders: Prevalence, comorbidity and convergent validity. Personality and Mental Health, 8(1), 42–51. <https://doi.org/10.1002/pmh.1241>

**136**. Trent, E. S., Viana, A. G., Raines, E. M., Woodward, E. C., Michael J. Zvolensky, M. J., et al. (2019). Exposure to parental threatening behaviors and internalizing psychopathology in a trauma-exposed inpatient adolescent sample. Journal of Nervous & Mental Disease, Publish Ahead of Print. <https://doi.org/10.1097/nmd.0000000000001058>

**137**. Hatzis, D., Dawe, S., Harnett, P., and Loxton, N. (2019). An investigation of the impact of childhood trauma on quality of caregiving in high risk mothers: Does maternal substance misuse confer additional risk? Child Psychiatry & Human Development, 50(5), 835–845. <https://doi.org/10.1007/s10578-019-00886-5>

**138**. Ghorbani, F., Khosravani, V., Mohammadzadeh, A., and Shadnia, S. (2019). The role of emotion dysregulation in the relation of childhood trauma to heroin craving in individuals with heroin dependence. Drug and Alcohol Dependence, 195, 132–139. <https://doi.org/10.1016/j.drugalcdep.2018.12.008>

**139.** Mohammadzadeh, A., Ganji, Z., Khosravani, V., Mohammadpanah Ardakan, A., and

Amirinezhad, A. (2019). Direct and indirect associations between perception of childhood trauma and suicidal ideation through emotion dysregulation in males who use heroin. Addictive Behaviors, 98:106011. <https://doi.org/10.1016/j.addbeh.2019.05.035>

**140.** Shipman, K., Zeman, J., Penza, S., and Champion, K. (2000). Emotion management skills in sexually maltreated and nonmaltreated girls: A developmental psychopathology perspective. Development and Psychopathology, 12, 47–62. <https://doi.org/10.1017/s0954579400001036>

**141**. Shenk, C. E., Noll, J. G., and Cassarly, J. A. (2009). A multiple mediational test of the relationship between childhood maltreatment and non-suicidal self-injury. Journal of Youth and Adolescence, 39(4), 335–342. <https://doi.org/10.1007/s10964-009-9456-2>

**142**. Chaplo, S. D., Kerig, P. K, Bennett, D. C., and Modrowski, C. A. (2015). The roles of emotion dysregulation and dissociation in the association between sexual abuse and self- injury among juvenile justice–involved youth. Journal of Trauma & Dissociation, 16(3), 272–285. <https://doi.org/10.1080/15299732.2015.989647>

**143**. Bierman, K. L., Kalvin, C. B., and Heinrichs, B. S. (2014). Early childhood precursors and adolescent sequelae of grade school peer rejection and victimization. Journal of Clinical Child & Adolescent Psychology, 44(3), 367–379.

<https://doi.org/10.1080/15374416.2013.873983>

**144**. Hébert, M., Langevin, R., and Charest, F. (2020). Disorganized attachment and emotion dysregulation as mediators of the association between sexual abuse and dissociation in preschoolers. Journal of Affective Disorders, 267, 220–228. <https://doi.org/10.1016/j.jad.2020.02.032>

**145**. Josephine Tejada, A., and Linder, S. M. (2018). The influence of child sexual abuse on preschool-aged children. Early Child Development and Care, 1–11. <https://doi.org/10.1080/03004430.2018.1542384>

**146**. Girard, M., Dugal, C., Hébert, M., and Godbout, N. (2020) Is my sex life ok? The mediating role of sexual anxiety in the association between childhood sexual abuse and sexual coercion against women. Journal of Child Sexual Abuse, 29(6), 717–733. <https://doi.org/10.1080/10538712.2020.1774697>

**147**. Villalta, L., Khadr, S., Chua, K-C., Kramer, T., Clarke, V., and Viner, R. M. (2020). Complex post-traumatic stress symptoms in female adolescents: the role of emotion dysregulation in impairment and trauma exposure after an acute sexual assault. European Journal of Psychotraumatology, 11(1):1710400. <https://doi.org/10.1080/20008198.2019.1710400>

**148**. Greenbaum, V.J. (2014). Commercial Sexual Exploitation and Sex Trafficking of Children in the United States. Current Problems in Pediatric and Adolescent Health Care, 44(9), 245- 269. <https://doi.org/10.1016/j.cppeds.2014.07.001>

**149**. Hopper, E. K. Polyvictimization and developmental trauma adaptations in sex trafficked youth. Journal of Child & Adolescent Trauma, 10(2):161–173, 2016. <https://doi.org/10.1007/s40653-016-0114-z>

**150.** Ahmed, S. P., Bittencourt-Hewitt, A., and Sebastian, C. L. (2015). Neurocognitive bases of emotion regulation development in adolescence. Developmental Cognitive Neuroscience, 15, 11–25. <https://doi.org/10.1016/j.dcn.2015.07.006>

**151.** Malter Cohen, M., Jing, D., Yang, R. R., Tottenham, N., Lee, F. S., and Casey, B. J. (2013). Early-life stress has persistent effects on amygdala function and development in mice and humans. Proceedings of the National Academy of Sciences, 110(45), 18274– 18278. <https://doi.org/10.1073/pnas.1310163110>

**152**. Fishbein, D., Warner, T., Krebs, C., Trevarthen, N., Flannery, B., and Hammond, J. (2008). Differential relationships between personal and community stressors and children’s neurocognitive functioning. Child Maltreatment, 14(4), 299–315. <https://doi.org/10.1177/1077559508326355>

**153**. Cicchetti, D., Rogosch, F. A., Gunnar, M. R., and Toth, S. L. (2010). The differential impacts of early physical and sexual abuse and internalizing problems on daytime cortisol rhythm in school-aged children. Child Devlopment, 81(1), 252–269. <https://doi.org/10.1111/j.1467-8624.2009.01393.x>

**154.** Rees, C. A., and Selwyn, J. (2009). Non-infant adoption from care: lessons for safeguarding children. Child: Care, Health and Development, 35(4), 561–567. <https://doi.org/10.1111/j.1365-2214.2009.00978.x>

**155.** Panlilio, C. C., Harden, B. J., and Harring, J. (2018). School readiness of maltreated preschoolers and later school achievement: The role of emotion regulation, language, and context. Child Abuse & Neglect, 75:82–91, 2018.https://doi.org/10.1016/j.chiabu.2017.06.004

**156**. Glaser, D. (2000). Child abuse and neglect and the brain–a review. Journal of Child Psychology and Psychiatry, 41(1), 97–116. https://doi.org/ 10.1111/1469-7610.00551

**157**. Rutter, M. L. (1999). Psychosocial adversity and child psychopathology. British Journal of Psychiatry, 174(6), 480–493. <https://doi.org/10.1192/bjp.174.6.480>

**3.1.4 Posttraumatic Stress Disorder (PTSD)**

**2**. Gratz, K. L., and Roemer, L. (2004). Multidimensional Assessment of Emotion Regulation and Dysregulation: Development, Factor Structure, and Initial Validation of the Difficulties in Emotion Regulation Scale. Journal of Psychopathology and Behavioral Assessment, 26, 41–54. <https://doi.org/10.1023/B:JOBA.0000007455.08539.94>

**88**. Dvir, Y., Ford, J. D., Hill, M., and Frazier, J. A. (2014). Childhood maltreatment, emotional dysregulation, and psychiatric comorbidities. Harvard Review of Psychiatry, 22(3), 149–161. https://doi.org/ 10.1097/hrp.0000000000000014

**89**. van der Kolk, B. A. (2005). Developmental trauma disorder: Toward a rational diagnosis for children with complex trauma histories. Psychiatric Annals, 35(5), 401–408. <https://doi.org/10.3928/00485713-20050501-06>

**90**. McLaughlin, K.A., Hatzenbuehler, M.L., Mennin, D.S., and Nolen-Hoeksema, S. Emotion dysregulation and adolescent psychopathology: A prospective study. Behaviour Research and Therapy, 49(9):544–554, 2011. <https://doi.org/10.1016/j.brat.2011.06.003>

**93**. Spinazozola, J., van der Kolk, B., and Ford, J.D. (2018). When nowhere is safe: Interpersonal Trauma and attachment adversity as antecedents of Posttraumatic Stress Disorder and Developmental Trauma Disorder. Journal of Traumatic Stress, 31 (5), 631- 642. <https://doi.org/10.1002/jts.22320>

**94.** Ford, J. D., Spinazzola, J., van der Kolk, B., and Grasso, D. (2018). Toward an

empirically-based Developmental Trauma Disorder diagnosis for children: Factor structure,

item characteristics, reliability and validity of the Developmental Trauma Disorder Semi

Structured Interview (DTD-SI). Journal of Clinical Psychiatry, 79(5), e1-e9.

<https://doi.org/doi.org/10.4088/JCP.17m11675>

**95**. Powers, A., Stevens, J. S., O'Banion, D., Stenson, A. F., Kaslow, N., Jovanovic, T., et al. (2020). Intergenerational transmission of risk for PTSD symptoms in African American children: The roles of maternal and child emotion dysregulation. Psychological Trauma: Theory, Research, Practice, and Policy. <https://doi.org/10.1037/tra0000543>

**96**. Kim, J., and Cicchetti, D. (2009). Longitudinal pathways linking child maltreatment, emotion regulation, peer relations, and psychopathology. Journal of Child Psychology and Psychiatry, 51(6):706–716, 2009. <https://doi.org/10.1111/j.1469-7610.2009.02202.x>

**102**. Lehmann, S., Breivik, K., Monette, S., and Minnis. H. (2020). Potentially traumatic events in foster youth, and association with DSM-5 trauma- and stressor related symptoms. Child Abuse & Neglect, 101:104374. <https://doi.org/10.1016/j.chiabu.2020.104374>

**142**. Chaplo, S. D., Kerig, P. K, Bennett, D. C., and Modrowski, C. A. (2015). The roles of emotion dysregulation and dissociation in the association between sexual abuse and self- injury among juvenile justice–involved youth. Journal of Trauma & Dissociation, 16(3), 272–285. <https://doi.org/10.1080/15299732.2015.989647>

**147**. Villalta, L., Khadr, S., Chua, K-C., Kramer, T., Clarke, V., and Viner, R. M. (2020). Complex post-traumatic stress symptoms in female adolescents: the role of emotion dysregulation in impairment and trauma exposure after an acute sexual assault. European Journal of Psychotraumatology, 11(1):1710400. <https://doi.org/10.1080/20008198.2019.1710400>

**158**. Horowitz, M. J. (2011). Stress response syndromes. 5th edition, Northvale, NJ.

**159**. Lanius, R. A., Vermetten, E., Loewenstein, R. J., Brand, B., Schmahl, C., Douglas Bremner, J., and Spiegel, D. (2010). Emotion modulation in PTSD: Clinical and neurobiological evidence for a dissociative subtype. American Journal of Psychiatry, 167(6), 640–647. <https://doi.org/10.1176/appi.ajp.2009.09081168>

**160**. Tull, M. T., Barrett, H. M., McMillan, E. S., and Roemer, L. (2007) A preliminary investigation of the relationship between emotion regulation difficulties and posttraumatic stress symptoms. Behavior Therapy, 38(3), 303–313. <https://doi.org/10.1016/j.beth.2006.10.001>

**161**. Seligowski, A. V., Lee, D. J., Bardeen, J. R., and Orcutt, H. K. (2014). Emotion regulation and posttraumatic stress symptoms: A meta-analysis. Cognitive Behaviour Therapy, 44(2), 87–102. <https://doi.org/10.1080/16506073.2014.980753>

**162**. Kaczkurkin, A. N., Zang, Y., Gay, N. G., Peterson, A. L., Yarvis, J. S., Borah, E. V., et al. (2007). Cognitive emotion regulation strategies associated with the DSM-5 posttraumatic stress disorder criteria. Journal of Traumatic Stress, 30(4), 343–350. <https://doi.org/10.1002/jts.22202>

**163**. Weissman, D. G., Bitran, D., Bryant Miller, A., Schaefer, J. D., Sheridan, M. A., and McLaughlin, K A. (2019). Difficulties with emotion regulation as a transdiagnostic mechanism linking child maltreatment with the emergence of psychopathology. Development and Psychopathology, 31(3), 899–915. <https://doi.org/10.1017/s0954579419000348>

**164**. Wolf, R. C., and Herringa, R. J. (2015). Prefrontal–amygdala dysregulation to threat in pediatric posttraumatic stress disorder. Neuropsychopharmacology, 41(3), 822–831. <https://doi.org/10.1038/npp.2015.209>

**165**. McCauley, E., Berk, M. S., Asarnow, J. R., Adrian, M., Cohen, J., Korslund, K., et al. (2018). Efficacy of dialectical behavior therapy for adolescents at high risk for suicide. JAMA Psychiatry, 75(8):777, 2018. <https://doi.org/10.1001/jamapsychiatry.2018.1109>

**166**. Briggs-Gowan, M. J., Carter, A. S., Clark, R., Augustyn, M., McCarthy, K. J., and Ford, J. D. (2010). Exposure to potentially traumatic events in early childhood: differential links to emergent psychopathology. Journal of Child Psychology and Psychiatry, 51(10), 1132-1140. <https://doi.org/10.1111/j.14697610.2010.02256.x>

**167**. Grasso, D. J., Ford, J. D., and Briggs-Gowan, M. J. (2013). Early life trauma exposure and stress sensitivity in young children. Journal of Pediatric Psychology, 38(1), 94-103. <https://doi.org/10.1093/jpepsy/jss101>

**168**. Scheeringa, M. S., Zeanah, C. H., Myers, L., and Putnam, F. P. (2003). New findings

on alternative criteria for PTSD in preschool children. Journal of the American Academy of Child & Adolescent Psychiatry, 42(5), 561–570. <https://doi.org/10.1097/01.chi.0000046822.95464.14>

**169**. Mongillo, E. A., Briggs-Gowan, M., Ford, J., and Carter, A. S. (2008). Impact of traumatic life events in a community sample of toddlers. Journal of Abnormal Child Psychology, 37(4), 455–468. <https://doi.org/10.1007/s10802-008-9283-z>

**170**. Viana, A. G., Woodward, E. C., Raines, E. M., Hanna, A. E., and Zvolensky, M. J. (2018). The role of emotional clarity and distress tolerance in deliberate self-harm in a sample of trauma-exposed inpatient adolescents at risk for suicide. General Hospital Psychiatry, 50, 119–124. <https://doi.org/10.1016/j.genhosppsych.2017.10.009>

**171**. Viana, A. G., Raines, E. M., Woodward, E. C., Hanna, A. E., Walker, R., and Zvolensky, M. J. (2018). The relationship between emotional clarity and suicidal ideation among trauma-exposed adolescents in inpatient psychiatric care: does distress tolerance matter? Cognitive Behaviour Therapy, 48(5), 430–444. <https://doi.org/10.1016/j.genhosppsych.2017.10.009>

**172**. van der Kolk, B. A., Roth, S., Pelcovitz, D., Sunday, S., and Spinazzola, J. (2005). Disorders of extreme stress: The empirical foundation of a complex adaptation to trauma. Journal of Traumatic Stress, 18(5), 389–399. <https://doi.org/10.1002/jts.20047>

**173**. Buckholdt, K. E., Weiss, N. H., Young, J., and Gratz, K. L. (2014). Exposure to violence, posttraumatic stress symptoms, and borderline personality pathology among adolescents in residential psychiatric treatment: The influence of emotion dysregulation. Child Psychiatry & Human Development, 46(6), 884–892. [https://doi.org/10.1007/s10578-014- 0528-5](https://doi.org/10.1007/s10578-014-%200528-5)

**174**. Espil, F. M., Viana, A. G., and Dixon, L. J. (2016). Post-traumatic stress disorder and depressive symptoms among inpatient adolescents: The underlying role of emotion regulation. Residential Treatment for Children & Youth, 33(1), 51–68. <https://doi.org/10.1080/0886571X.2016.1159939>

**175**. Marsee, M. A. (2008) Reactive aggression and posttraumatic stress in adolescents affected by Hurricane Katrina. Journal of Clinical Child and Adolescent Psychology, 37(3), 519–529. <https://doi.org/10.1080/15374410802148152>

**176**. Bennett, D. C., Modrowski, C. A., Chaplo, S. D., and Kerig, P. K. (2016). Facets of emotion dysregulation as mediators of the association between trauma exposure and posttraumatic stress symptoms in justice-involved adolescents. Traumatology, 22(3), 174– 183. <https://doi.org/10.1037/trm0000085>

**177**. Miller, M. A., and Marsee, M. A. (2019). Emotional reactivity and antisocial behavior relative to posttraumatic stress symptom expression: a latent profile analysis. Journal of Abnormal Child Psychology, 47(8), 1339–1350. https://doi.org/10.1007/s10802-019-00514-9 **178**. Reich, W. A. (2014). Mental health screening outcomes among justice-involved youths under community supervision. Journal of Offender Rehabilitation, 53(3), 211–230. <https://doi.org/10.1080/10509674.2014.887607>

**179.** Kelley, L. P., Weathers, F. W., McDevitt-Murphy, M. E., Eakin, D. E., and Flood, A. M. (2009). A comparison of PTSD symptom patterns in three types of civilian trauma. Journal of Traumatic Stress, 22(3), 227–235. <https://doi.org/10.1002/jts.20406>

**180**. Ford, J. D., Gagnon, K., Connor, D. F., and Pearson, G. (2011). History of interpersonal violence, abuse, and nonvictimization trauma and severity of psychiatric symptoms among children in outpatient psychiatric treatment. Journal of Interpersonal Violence, 26(16), 3316–3337. <https://doi.org/10.1177/0886260510393009>

**181.** Naomi Breslau, N. (2009). The epidemiology of trauma, PTSD, and other posttrauma disorders. Trauma, Violence, & Abuse, 10(3), 198–210. <https://doi.org/10.1177/1524838009334448>

**182**. Salazar, A. M., Keller, T. E., Gowen, L. K., and Courtney, M. E. (2012). Trauma exposure and PTSD among older adolescents in foster care. Social Psychiatry and Psychiatric Epidemiology, 48(4), 545–551. <https://doi.org/10.1007/s00127-012-0563-0>

**183**. Valdez, C. E., Bailey, B. E., Santuzzi, A. M., and Lilly, M. M. (2014). Trajectories of depressive symptoms in foster youth transitioning into adulthood. Child Maltreatment, 19(3- 4), 209-218. <https://doi.org/10.1177/1077559514551945>

**184**. Kelly, N. R., Tanofsky-Kraff, M., Vannucci, A., Ranzenhofer, L. M., Altschul, A. M., Natasha A. Schvey, N. A., et al. (2016). Emotion dysregulation and loss-of-control eating in children and adolescents. Health Psychology, 35(10), 1110–1119. <https://doi.org/10.1037/hea0000389>

**185**. Weiss, N. H., Tull, M. T., Lavender, J., and Gratz, K. L. (2013). Role of emotion dysregulation in the relationship between childhood abuse and probable PTSD in a sample of substance abusers. Child Abuse & Neglect, 37(11), 944–954. <https://doi.org/10.1016/j.chiabu.2013.03.014>

**186**. Charak R., Byllesby, B. M., Fowler, J. C., Sharp, C., Elhai, J. D., and Frueh, B. C. (2019). Assessment of the revised difficulties in emotion regulation scales among adolescents and adults with severe mental illness. Psychiatry Research, 279, 278–283. <https://doi.org/10.1016/j.psychres.2019.04.010>

**187**. Charak, R., Ford, J. D., Modrowski, C. A., and Kerig, P. K. (2018). Polyvictimization, emotion dysregulation, symptoms of posttraumatic stress disorder, and behavioral health problems among justice-involved youth: a latent class analysis. Journal of Abnormal Child Psychology, 47(2), 287–298. <https://doi.org/10.1007/s10802-018-0431-9>

**188**. Rauch, S., and Foa, E. (2006). Emotional processing theory (EPT) and exposure therapy for PTSD. Journal of Contemporary Psychotherapy, 36(2):61–65.

https://doi.org/10.1007/s10879-006- 9008-y

**189**. Woodward, E. C., Viana, A. G., Trent, E. S, Raines, E. M., Zvolensky, M. J., and Storch, E. A. (2019). Emotional nonacceptance, distraction coping and PTSD symptoms in a trauma-exposed adolescent inpatient sample. Cognitive Therapy and Research, 44(2), 412– 419. <https://doi.org/10.1007/s10608-019-10065-4>

**190**. Biederman, J., Wozniak, J., Martelon, M. K., Spencer, T. J., Woodworth, Y., Joshi, G., et al. (2013). Can pediatric bipolar-I disorder be diagnosed in the context of posttraumatic stress disorder? A familial risk analysis. Psychiatry Research, 208(3), 215–224. <https://doi.org/10.1016/j.psychres.2013.05.011>

**191**. Liu, J., Subramaniam, M., Ann Chong, S., and Mahendran, R. (2019). A systematic examination of cognitive emotion regulation strategies, global emotion dysregulation, and cognitive insight in relation to posttraumatic stress disorder symptoms among trauma exposed patients with early nonaffective psychosis. Psychological Trauma: Theory, Research, Practice, and Policy. <https://doi.org/10.1037/tra0000531>

**192.** Ford, J. D., Grasso, D., Greene, C., Levine, J., Spinazzola, J., and van der Kolk, B. (2013). Clinical significance of a proposed developemntal trauma disorder diagnosis:

Results of an international survey of clinicians. Journal of Clinical Psychiatry, 74, 841–849. <https://doi.org/10.4088/JCP.12m08030>

**3.1.5 Non- Suicidal Self Injury (NSSI) and Suicidality**

**2**. Gratz, K. L., and Roemer, L. (2004). Multidimensional Assessment of Emotion Regulation and Dysregulation: Development, Factor Structure, and Initial Validation of the Difficulties in Emotion Regulation Scale. Journal of Psychopathology and Behavioral Assessment, 26, 41–54. <https://doi.org/10.1023/B:JOBA.0000007455.08539.94>

**31**. American Psychiatric Association. (2013). Diagnostic and Statistical Manual of Mental Disorders (DSM-5®), Fifth Edition. Washington, DC: American Psychiatric Association.

**81.** M. Linehan. (1993) Cognitive-behavioral treatment for borderline personality disorder. New York, NY: Guilford Press.

**82.** Crowell, S. E., Beauchaine, T. P., McCauley, E., Smith, C. J., Stevens, A. L., and Sylvers, P. (2005). Psychological, autonomic, and serotonergic correlates of parasuicide among adolescent girls. Development and Psychopathology, 17(04), 1105–1127. <https://doi.org/10.1017/s0954579405050522>

**83**. Yen, S., Weinstock, L. M., Andover, M. S., Sheets, E. S., Selby, E. A., and Spirito, A. (2012). Prospective predictors of adolescent suicidality: 6-month post-hospitalization follow-up. Psychological Medicine, 43(5), 983–993. <https://doi.org/10.1017/s0033291712001912>

**128.** Xu Peh, C., Shahwan, S., Fauziana, R., Mahesh, M. V., Sambasivam, R., Zhang, Y. (2017). Emotion dysregulation as a mechanism linking child maltreatment exposure and self- harm behaviors in adolescents. Child Abuse & Neglect, 67:383–390. <https://doi.org/10.1016/j.chiabu.2017.03.013>

**131.** Amelia Arria, Kevin O'Grady, Kimberly Caldeira, Kathryn Vincent, Holly Wilcox, and Eric Wish. Suicide ideation among college students: A multivariate analysis. Archives of Suicide Research, 13(3):230–246, 2009. <https://doi.org/10.1080/13811110903044351>

**142**. Chaplo, S. D., Kerig, P. K, Bennett, D. C., and Modrowski, C. A. (2015). The roles of emotion dysregulation and dissociation in the association between sexual abuse and self- injury among juvenile justice–involved youth. Journal of Trauma & Dissociation, 16(3), 272–285. https://doi.org/10.1080/15299732.2015.989647

**170**. Viana, A. G., Woodward, E. C., Raines, E. M., Hanna, A. E., and Zvolensky, M. J. (2018). The role of emotional clarity and distress tolerance in deliberate self-harm in a sample of trauma-exposed inpatient adolescents at risk for suicide. General Hospital Psychiatry, 50, 119–124. <https://doi.org/10.1016/j.genhosppsych.2017.10.009>

**193**. Nock, M. K. (2010). Self-injury. Annual Review of Clinical Psychology, 6(1):339–363, 2010. <https://doi.org/10.1146/annurev.clinpsy.121208.131258>

**194**. Glenn, C. R., and Klonsky, E. D. (2013). Nonsuicidal self-injury disorder: An empirical investigation in adolescent psychiatric patients. Journal of Clinical Child & Adolescent Psychology, 42(4), 496–507. <https://doi.org/10.1080/15374416.2013.794699>

**195**. Suyemoto, K. L. (1998). The functions of self-mutilation. Clinical Psychology Review, 18(5), 531–554. <https://doi.org/10.1016/s0272-7358(97)00105-0>

**196**. Gratz, K. L., and Roemer, L. (2008). The relationship between emotion dysregulation and deliberate self-harm among female undergraduate students at an urban commuter university. Cognitive Behaviour Therapy, 37(1), 14–25. <https://doi.org/10.1080/16506070701819524>

**197**. Nock, M. K., and Favazza, A. R. (2009). Nonsuicidal self-injury: Definition and classification. In Understanding nonsuicidal self-injury: Origins, assessment and treatment, ed. M. K. Nock, (American Psychological Association, Washington, DC), 9-18.

**198**. Nock, M. K., and Prinstein, M. J. (2005). Contextual features and behavioral functions of self-mutilation among adolescents. Journal of Abnormal Psychology, 114(1), 140–146. https://doi.org/ 10.1037/0021-843x.114.1.140

**199**. Chapman, A. L., Gratz, K. L., and Brown, M. Z. (2016). Solving the puzzle of deliberate self-harm: The experiential avoidance model. Behaviour Research and Therapy, 44(3), 371–394. <https://doi.org/10.1016/j.brat.2005.03.005>

**200**. Nock, M. K., Prinstein, M. J., and K, S. Sterba. (2009). Revealing the form and function of self-injurious thoughts and behaviors: A real-time ecological assessment study among adolescents and young adults. Journal of Abnormal Psychology, 118(4), 816–827. https://doi.org/ 10.1037/a0016948

**201**. Caro-Cañizares, I., Díaz de Neira-Hernando, M., Pfang, B., Baca-Garcia, E., and Carballo, J. J. (2018). The strengths and difficulties questionnaire -dysregulation profile, non-suicidal self-injury behaviors and the mediating role of stressful life events. The Spanish Journal of Psychology. <https://doi.org/10.1017/sjp.2018.23>

**202.** Nock, M. K., Joiner, Jr T. E., Gordon, K. H., Lloyd-Richardson, E., and Prinstein, M. J. (2006). Non-suicidal self-injury among adolescents: Diagnostic correlates and relation to suicide attempts. Psychiatry Research, 144(1), 65–72. <https://doi.org/10.1016/j.psychres.2006.05.010>

**203.** Somma, A., Sharp, C., Borroni, S., and Fossati, A. (2016). Borderline personality disorder features, emotion dysregulation and non-suicidal self-injury: Preliminary findings in a sample of community-dwelling Italian adolescents. Personality and Mental Health, 11(1), 23–32. <https://doi.org/10.1002/pmh.1353>

**204**. Andover, M. S., Blair W Morris, B. W., Wren, A., and Bruzzese, M. E. (2012). The co- occurrence of non-suicidal self-injury and attempted suicide among adolescents: distinguishing risk factors and psychosocial correlates. Child and Adolescent Psychiatry and Mental Health, 6 (1). <https://doi.org/10.1186/1753-2000-6-11>

**205**. Rajappa, K., Gallagher, M., and Miranda, R. (2011). Emotion dysregulation and vulnerability to suicidal ideation and attempts. Cognitive Therapy and Research, 36(6):833–839. <https://doi.org/10.1007/s10608-011-9419-2>

**206**. Perez, J., Venta, A., Garnaat, S., and Sharp, C. (2012). The difficulties in emotion regulation scale: Factor structure and association with nonsuicidal self-injury in adolescent inpatients. Journal of Psychopathology and Behavioral Assessment, 34(3):393–404. [https://doi.org/10.1007/s10862- 012-9292-7](https://doi.org/10.1007/s10862-%20012-9292-7)

**207**. Santangelo, P. S., Koenig, J., Funke, V., Parzer, P., Resch, F., Ebner-Priemer, U. W., and Kaess, M. (2016). Ecological momentary assessment of affective and interpersonal instability in adolescent non-suicidal self-injury. Journal of Abnormal Child Psychology, 45(7), 1429–1438. https://doi.org/ 10.1007/s10802-016-0249-2

**208**. Gratz, K. L., and Chapman, A. L. (2007). The role of emotional responding and childhood maltreatment in the development and maintenance of deliberate self-harm among male undergraduates. Psychology of Men & Masculinity, 8(1), 1–14. <https://doi.org/10.1037/1524-9220.8.1.1>

**209**. Adrian, M., Zeman, J., and Veits, G. (2011). Methodological implications of the affect revolution: a 35-year review of emotion regulation assessmentin children. Journal of Experimental Child Psychology, 110, 171–197. <https://doi.org/10.1016/j.jecp.2011.03.009>

**210**. Ann Emery, A., Heath, N. L., and Mills, D. J. (2015). Basic psychological need satisfaction, emotion dysregulation, and non-suicidal self-injury engagement in young

adults: An application of self-determination theory. Journal of Youth and Adolescence, 45(3), 612–623. <https://doi.org/10.1007/s10964-015-0405-y>

**211**. Bjureberg, J., Sahlin, H., Hedman-Lagerlöf, E., Gratz, K. L., T. Tull, M. T., Jokinen, J., et al. (2018). Extending research on emotion regulation individual therapy for adolescents (ERITA) with nonsuicidal self-injury disorder: open pilot trial and mediation analysis of a novel online version. BMC Psychiatry, 18(1). <https://doi.org/10.1186/s12888-018-1885-6>

**212**. Sadeh, N., Londahl-Shaller, E. A., Piatigorsky, A., Fordwood, S., Stuart, B. K., McNiel, D. E., et al. (2014). Functions of non-suicidal self-injury in adolescents and young adults with borderline personality disorder symptoms. Psychiatry Research, 216(2), 217–222. <https://doi.org/10.1016/j.psychres.2014.02.018>

**213**. Nakar, O., Brunner, R., Schilling, O., Chanen, A., Fischer, G., Parzer, P., et al. (2016). Developmental trajectories of self-injurious behavior, suicidal behavior and substance misuse and their association with adolescent borderline personality pathology. Journal of Affective Disorders, 197, 231–238. <https://doi.org/10.1016/j.jad.2016.03.029>

**214**. Muehlenkamp, J., Brausch, A., Quigley, K., and Whitlock, J. (2012). Interpersonal features and functions of nonsuicidal self-injury. Suicide and Life-Threatening Behavior, 43(1), 67–80. <https://doi.org/10.1111/j.1943-278x.2012.00128.x>

**215**. Klonsky, E. D., Glenn, C. R., Styer, D. M., Olino, T. M., and Washburn, J. J. (2015). The functions of nonsuicidal self-injury: converging evidence for a two-factor structure. Child and

Adolescent Psychiatry and Mental Health, 9(1). <https://doi.org/10.1186/s13034-015-0073-4>

**216**. Plener, P. L., Kapusta, N. D., Kölch, M.G., Kaess, M., and Brunner, R. (2012). Nicht- suizidale Selbstverletzung als eigenständige Diagnose. [Non-suicidal self-injury as autonomous diagnosis – implications for research and clinic of the DSM-5 proposal to establish the diagnosis of non-suicidal self-injury in adolescents]. Zeitschrift für Kinder- und Jugendpsychiatrie und Psychotherapie, 40(2), 113–120. [https://doi.org/10.1024/1422- 4917/a000158](https://doi.org/10.1024/1422-%204917/a000158)

**217**. Turner, B. J., Yiu, A., Layden, B. K., Claes, L., Zaitsoff, S., and Chapman, A. L. (2015). Temporal associations between disordered eating and nonsuicidal self-injury: Examining symptom overlap over 1 year. Behavior Therapy, 46(1), 125–138. <https://doi.org/10.1016/j.beth.2014.09.002>

**218.** Duggan, J., Heath, N., and Hu, T. (2015). Non-suicidal self-injury maintenance and cessation among adolescents: a one-year longitudinal investigation of the role of objectified body consciousness, depression and emotion dysregulation. Child and Adolescent

Psychiatry and Mental Health, 9(1). <https://doi.org/10.1186/s13034-015-0052-9>

**219**. Adrian, M., McCauley, E., Berk, M. S., Asarnow, J. R., Korslund, K., Avina, C., et al.

(2019). Predictors and moderators of recurring self-harm in adolescents participating in a comparative treatment trial of psychological interventions. The Journal of Child Psychology and Psychiatry, 60(10):1123-1132, 2019. [https://doi.org/10.1111/jcpp.13099. Epub 2019 Jul 30](https://doi.org/10.1111/jcpp.13099.%20Epub%202019%20Jul%2030)

**220**. Hilt, L. M., Cha, C. B., and Nolen-Hoeksema, S. (2008). Nonsuicidal self-injury in young adolescent girls: Moderators of the distress-function relationship. Journal of Consulting and Clinical Psychology, 76(1), 63–71. <https://doi.org/10.1037/0022-006x.76.1.63>

**221**. Crowell, S. E., Beauchaine, T. P., and Linehan, M M. (2009). A biosocial developmental model of borderline personality: Elaborating and extending Linehan’s theory. Psychological Bulletin, 135(3):495–510. <https://doi.org/10.1037/a0015616>

**222**. Sim, L., Adrian, M., Zeman, J., Cassano, M., and Friedrich, W. N. (2009). Adolescent deliberate self-harm: Linkages to emotion regulation and family emotional climate. Journal of Research on Adolescence, 19(1), 75–91. <https://doi.org/10.1111/j.1532-7795.2009.00582.x>

**223**. Kaess, M., Parzer, P., Mattern, M., Plener, P. L., Bifulco, A., Resch, F., and Brunner, R. (2013). Adverse childhood experiences and their impact on frequency, severity, and the individual function of nonsuicidal self-injury in youth. Psychiatry Research, 206(2-3), 265– 272. <https://doi.org/10.1016/j.psychres.2012.10.012>

**224**. Titelius, E. N., Cook, E., Spas, J., Orchowski, L., Kivisto, K., O’Brien, K., et al. (2017). Emotion dysregulation mediates the relationship between child maltreatment and non- suicidal self-injury. Journal of Aggression, Maltreatment & Trauma, 27(3), 323–331. <https://doi.org/10.1080/10926771.2017.1338814>

**225**. Fraser, G., Stewart Wilson, M., Anne Garisch, J., Robinson, K., Brocklesby, M., Kingi, T., et al. (2017). Non-suicidal self-injury, sexuality concerns, and emotion regulation among sexually diverse adolescents: A multiple mediation analysis. Archives of Suicide Research, 22(3), 432–452. <https://doi.org/10.1080/13811118.2017.1358224>

**226**. Ybarra, M. L., Mitchell, K. J., Kosciw, J. G., and Korchmaros, J. D. (2014). Understanding linkages between bullying and suicidal ideation in a national sample of LGB and heterosexual youth in the United States. Prevention Science, 16(3), 451–462. <https://doi.org/10.1007/s11121-014-0510-2>

**227**. Shneidman, E. S. (1993). Suicide as psychache. The Journal of Nervous and Mental Disease, 181(3), 145–147. <https://doi.org/10.1097/00005053-199303000-00001>

**228**. Ringel, E. (1993). Der Selbstmord [The Suicide]. Maudrich, 5th edition, Wien.

**229**. Gliatto, M. F. and Rai, A. R. (1999). Evaluation and treatment of patients with suicidal ideation. American Family Physician, 59(6), 1500–1506.

**230**. Borges, G., Nock, M. K., Haro Abad, J. M., Hwang, I., Sampson, N. A., Alonso, J., et al. (2010). Twelve-month prevalence of and risk factors for suicide attempts in the World Health Organization world mental health surveys. Journal of Clinical Psychiatry, 71(12), 1617–1628. <https://doi.org/10.4088/JCP.08m04967blu>. Epub 2010 Aug 24

**231**. Nock M. K., Borges, G., Bromet, E. J, Cha, C.B., Kessler, R. C., and Lee, S. (2008). Suicide and suicidal behavior. Epidemiologic Reviews, 30(1), 133–154. <https://doi.org/10.1093/epirev/mxn002>

**232**. Wolff, J. C, Davis, S., Liu, R. T., Cha, C. B., Cheek, S. M., Nestor, B. A., et al. (2017). Trajectories of suicidal ideation among adolescents following psychiatric hospitalization. Journal of Abnormal Child Psychology, 46(2), 355–363. [https://doi.org/10.1007/s10802-017- 0293-6](https://doi.org/10.1007/s10802-017-%200293-6)

**233**. Pan, L. A., Hassel, S., Segreti, A. M., Nau, S. A., Brent, D. A., and Phillips, M. L. (2013). Differential patterns of activity and functional connectivity in emotion processing neural circuitry to angry and happy faces in adolescents with and without suicide attempt. Psychological Medicine, 43(10), 2129–2142. <https://doi.org/10.1017/s0033291712002966>

**234**. Selby, E. A., Yen, S., and Spirito, A. (2013). Time varying prediction of thoughts of death and suicidal ideation in adolescents: Weekly ratings over 6-month follow-up. Journal of Clinical Child & Adolescent Psychology, 42(4), 481–495. <https://doi.org/10.1080/15374416.2012.736356>

**235**. Tamás, Z., Kovacs, M., Gentzler, A. L., Tepper, P., Gádoros, J., Kiss E., Kapornai, K., and Vetró, Á. (2007). The relations of temperament and emotion self-regulation with

suicidal behaviors in a clinical sample of depressed children in Hungary. Journal of Abnormal Child Psychology, 35(4), 640–652. <https://doi.org/10.1007/s10802-007-9119-2>

**236**. Saffer, B. Y., Glenn, C. R., and Klonsky, E. D. (2014). Clarifying the relationship of parental bonding to suicide ideation and attempts. Suicide and Life-Threatening Behavior, 45(4), 518–528. <https://doi.org/10.1111/sltb.12146>

**237**. Anestis, M. D., Kleiman, E. M., Lavender, J. M., Tull, M. T., and Gratz, K. L. (2014). The pursuit of death versus escape from negative affect: An examination of the nature of the relationship between emotion dysregulation and both suicidal behavior and non-suicidal self-injury. Comprehensive Psychiatry, 55(8):1820–1830. <https://doi.org/10.1016/j.comppsych.2014.07.007>

**238**. Esposito, C., Spirito, A., Boergers, J., and Donaldson, D. (2003). Affective, behavioral, and cognitive functioning in adolescents with multiple suicide attempts. Suicide and Life Threatening Behavior, 33(4):389–399. <https://doi.org/10.1521/suli.33.4.389.25231>

**3.1.6 Eating Disorder**

**2.** Gratz, K. L., and Roemer, L. (2004). Multidimensional Assessment of Emotion Regulation and Dysregulation: Development, Factor Structure, and Initial Validation of the Difficulties in Emotion Regulation Scale. Journal of Psychopathology and Behavioral Assessment, 26, 41–54. <https://doi.org/10.1023/B:JOBA.0000007455.08539.94>

**31**. American Psychiatric Association. (2013). Diagnostic and Statistical Manual of Mental Disorders (DSM-5®), Fifth Edition. Washington, DC: American Psychiatric Association.

**184**. Kelly, N. R., Tanofsky-Kraff, M., Vannucci, A., Ranzenhofer, L. M., Altschul, A. M., Natasha A. Schvey, N. A., et al. (2016). Emotion dysregulation and loss-of-control eating in children and adolescents. Health Psychology, 35(10), 1110–1119. <https://doi.org/10.1037/hea0000389>

**239**. World Health Organization (WHO). (2019). Icd-10 (International Statistical Classification of Diseases and Health Related Problems). <https://icd.who.int/browse10/2019/en>

**240**. Wisniewski, L., Safer, D., and Chen, E. (2007) Dialectical behavior therapy and eating disorders. In L. A. Dimeff and K. Koerner, editors, Dialectical behavior therapy in clinical practice: Applications across disorders and settings. New York, NY: The Guilford Press.

**241**. Knatz, S., Braden, A., and Kerri N. (2015). Boutelle. Parent coaching model for adolescents with emotional eating. Eating Disorders, 23(4), 377–386. <https://doi.org/10.1080/10640266.2015.1044352>

**242.** Racine, S. E., and Wildes, J. E. (2013). Emotion dysregulation and symptoms of anorexia nervosa: The unique roles of lack of emotional awareness and impulse control difficulties when upset. International Journal of Eating Disorders, 46, 713–720. [https://doi.org/10.1002/eat.22145. Epub 2013 Jun 11](https://doi.org/10.1002/eat.22145.%20Epub%202013%20Jun%2011)

**243**. Cimbolli, P., Quiñones, Á., Ugarte, C., and De Pascale, A. (2017). Studio pilota sui disturbi della nutrizione e dell’alimentazione in età pediatrica e i disturbi dell’umore: comorbilità o tratti prodromici? Rivista di Psichiatria, 52(1):32–39. <https://doi.org/10.1708/2631.27052>

**244**. Haynos, A. F., and Fruzzetti, A. E. (2011). Anorexia nervosa as a disorder of emotion dysregulation: Evidence and treatment implications. Clinical Psychology: Science and Practice, 18(3):183–202. <https://doi.org/10.1111/j.1468-2850.2011.01250>.x

**245.** Monell, E., Clinton, D., and Birgegård, A. (2018). Emotion dysregulation and eating disorders- associations with diagnostic presentation and key symptoms. International Journal of Eating Disorders, 51(8):921–930. <https://doi.org/10.1002/eat.22925>

**246.** Safer, D. L., Telch, C. G, and Chen, E. Y. (2009). Dialectical behavior therapy for binge eating and bulimia. New York, NY: The Guilford Press.

**247**. Lavender, J. M., Wonderlich, S. A., Peterson, C. B., Crosby, R. D., Engel, S. G., Mitchell, J. E., et al. (2014). Dimensions of emotion dysregulation in bulimia nervosa. European Eating Disorders Review, 22(3), 212–216. <https://doi.org/10.1002/erv.2288>

**248.** Hansson, E., Daukantaité, D., and Johnsson, P. (2017). Disordered eating and emotion dysregulation among adolescents and their parents. BMC Psychology, 5(1). <https://doi.org/10.1186/s40359-017-0180-5>

**249**. Hughes-Scalise, A., and Connell, A. (2014). The roles of adolescent attentional bias and parental invalidation of sadness in significant illness: A comparison between eating disorders and chronic pain. Eating Behaviors, 15(3), 493–501. <https://doi.org/10.1016/j.eatbeh.2014.06.007>

**250.** Gilboa-Schechtman, E., Avnon, L., Zubery, E., and Jeczmien, P. (2006). Emotional processing in eating disorders: specific impairment or general distress related deficiency? Depression and Anxiety, 23(6), 331–339. <https://doi.org/10.1002/da.20163>

**251**. Gilbert, K. E. (2012). The neglected role of positive emotion in adolescent psychopathology. Clinical Psychology Review, 32(6):467–481. <https://doi.org/10.1016/j.cpr.2012.05.005>

**252**. Racine, S. E., and Wildes, J. E. (2015). Dynamic longitudinal relations between emotion regulation difficulties and anorexia nervosa symptoms over the year following intensive treatment. Journal of Consulting and Clinical Psychology, 83(4), 785–795. <https://doi.org/10.1037/ccp0000011>

**253**. Mills, P., Frances Newman, E., Jill Cossar, J., and George Murray, G. (2015). Emotional maltreatment and disordered eating in adolescents: Testing the mediating role of emotion regulation. Child Abuse & Neglect, 39, 156–166. <https://doi.org/10.1016/j.chiabu.2014.05.011>

**254**. Hansson, E., Daukantaite, D., and Johnsson, P. (2016). Typical patterns of disordered eating among Swedish adolescents: associations with emotion dysregulation, depression, and self-esteem. Journal of Eating Disorders, 4(1). <https://doi.org/10.1186/s40337-016-0122-2>

**255.** Anderson, L. K., Claudat, K., Cusack, A., Brown, T. A., Trim, J., and Rockwell, R. (2018). Differences in emotion regulation difficulties among adults and adolescents across eating disorder diagnoses. Journal of Clinical Psychology, 74(10), 1867–1873. <https://doi.org/10.1002/jclp.22638>

**256**. Jakovina, T., Crnkovic Batista, M., Razic Pavicic, A., Zuric Jakovina, I., and Begovac, I. (2018). Emotional dysregulation and attachment dimensions in female patients with bulimia nervosa. Psychiatria Danubina, 30(1), 72–78. <https://doi.org/10.24869/psyd.2018.72>

**257**. Laghi, F., Bianchi, D., Pompili, S., Lonigro, A., and Baiocco, R. (2018). Metacognition, emotional functioning and binge eating in adolescence: the moderation role of need to control thoughts. Eating and Weight Disorders - Studies on Anorexia, Bulimia and Obesity, 23(6), 861–869. <https://doi.org/10.1007/s40519-018-0603-1>

**258**. Boutelle, K. N., Braden, A., Knatz-Peck, S, Anderson, L. K., and Rhee, K. E. (2018). An open trial targeting emotional eating among adolescents with overweight or obesity. Eating Disorders, 26(1), 79–91. <https://doi.org/10.1080/10640266.2018.1418252>

**259**. Wiser, S., and Telch, C. F. (1999). Dialectical behavior therapy for binge-eating disorder. Journal of Clinical Psychology, 55(6), 755–768. https://doi.org/10.1002/(sici)1097- 4679(199906)55:6<755::aid-jclp8>3.0.co;2-r

**260**. Segal, A. (2016). Differences in emotion regulation along the eating disorder spectrum: Cross sectional study in adolescents out patient care. Journal of Psychology & Clinical Psychiatry, 6(1). <https://doi.org/10.15406/jpcpy.2016.06.00314>

**261**. Rai, T., Mainali, P., Raza, A., Rashid, J., and Rutkofsky, I. (2019). Exploring the link between emotional child abuse and anorexia nervosa: A psychopathological correlation. Cureus. <https://doi.org/10.7759/cureus.5318>

**262.** McDonald, C. E., Rossell, S. L., and Phillipou, A. (2019). The comorbidity of eating disorders in bipolar disorder and associated clinical correlates characterised by emotion dysregulation and impulsivity: A systematic review. Journal of Affective Disorders, 259, 228–243. <https://doi.org/10.1016/j.jad.2019.08.070>

**263**. Slane, J. D., Klump, K. L., McGue, M., and Iacono, G. (2014). Genetic and environmental factors underlying comorbid bulimic behaviours and alcohol use disorders: A moderating role for the dysregulated personality cluster? European Eating Disorders Review, 22(3), 159–169. <https://doi.org/10.1002/erv.2284>

**3.1.7 Oppositional Defiant Disorder (ODD), Conduct Disorder (CD) and Disruptive Mood Dysregulation Disorder (DMDD)**

**31**. American Psychiatric Association. (2013). Diagnostic and Statistical Manual of Mental Disorders (DSM-5®), Fifth Edition. Washington, DC: American Psychiatric Association.

**78**. Kochman, F.J., Hantouche, E.G., Ferrari, P., Lancrenon, S., Bayart, D., and Akiskal, H.S. (2005). Cyclothymic temperament as a prospective predictor of bipolarity and suicidality in children and adolescents with major depressive disorder. Journal of Affective Disorders, 85 (1-2), 181-189. <https://doi.org/10.1016/j.jad.2003.09.009>

**112**. Sevecke, K., Franke, S., Kosson, D., and Krischer, M. (2016). Emotional dysregulation and trauma predicting psychopathy dimensions in female and male juvenile offenders. Child and Adolescent Psychiatry and Mental Health, 10(1). <https://doi.org/10.1186/s13034-016-01307>

**118**. Nederlof , E., Van der Ham, J. M., Dingemans, P. M. J. A., and Oei, T. I. (2010). The relation between dimensions of normal and pathological personality and childhood maltreatment in incarcerated boys. Journal of Personality Disorders, 24(6), 746–762. <https://doi.org/10.1521/pedi.2010.24.6.746>

**264**. Ford, J. D. (2002). Traumatic victimization in childhood and persistent problems with oppositional defiance. Journal of Aggression, Maltreatment & Trauma, 6(1), 25–58. <https://doi.org/10.1300/J146v06n01_03>

**265**. Steiner, H., and Remsing, L. (2007). Practice parameter for the assessment and treatment of children and adolescents with oppositional defiant disorder. Journal of the American Academy of Child & Adolescent Psychiatry, 46(1), 126–141. <https://doi.org/10.1097/01.chi.0000246060.62706.af>

**266.** Déry, M., Lapalme, M., Jagiellowicz, J., Poirier, M., Temcheff, C., and Toupin, J. (2016). Predicting depression and anxiety from oppositional defiant disorder symptoms in elementary school-age girls and boys with conduct problems. Child Psychiatry & Human Development, 48(1), 53–62. <https://doi.org/10.1007/s10578-016-0652-5>

**267**. Muratori, P., Pisano, S., Milone, A., and Masi, G. (2017). Is emotional dysregulation a risk indicator for auto-aggression behaviors in adolescents with oppositional defiant disorder? Journal of Affective Disorders, 208:110–112.

<https://doi.org/10.1016/j.jad.2016.08.052>

**268.** Bradshaw, C. P., Mitchell, M. M., and Leaf, P. J. (2009). Examining the effects of schoolwide positive behavioral interventions and supports on student outcomes. Journal of Positive Behavior Interventions, 12(3), 133–148. <https://doi.org/10.1177/1098300709334798>

**269**. Blandon, A. Y., Calkins, S. D., Grimm, K. J, Keane, S. P., and O'Brien, M. (2010). Testing a developmental cascade model of emotional and social competence and early peer acceptance. Development and Psychopathology, 22(4), 737–748. <https://doi.org/10.1017/s0954579410000428>

**270**. Martel, M. M., Gremillion, M. L., and Roberts, B. (2012). Temperament and common disruptive behavior problems in preschool. Personality and Individual Differences, 53(7), 874–879. <https://doi.org/10.1016/j.paid.2012.07.011>

**271.** Cavanagh, M., Quinn, D., Duncan, D., Graham, T., and Balbuena, L. (2016). Oppositional defiant disorder is better conceptualized as a disorder of emotional regulation. Journal of Attention Disorders, 21(5), 381–389. <https://doi.org/10.1177/1087054713520221>

**272**. Frick, M. A., Forslund, T., Fransson, M., Johansson, M., Bohlin, G., and Brocki, K. C. (2018). The role of sustained attention, maternal sensitivity, and infant temperament in the development of early self-regulation. British Journal of Psychology, 109(2), 277–298. <https://doi.org/10.1111/bjop.12266>

**273**. Marion Mitchison, G., Margo Liber, J., Kr. Hannesdottir; D., and Njardvik, U. (2019). Emotion dysregulation, ODD and conduct problems in a sample of five and six-year-old children. Child Psychiatry & Human Development, 51(1), 71–79. <https://doi.org/10.1007/s10578-019-00911-7>

**274**. Sagar-Ouriaghli, I., Lievesley, K., and Santosh, P. J. (2018). Propranolol for treating emotional, behavioural, autonomic dysregulation in children and adolescents with autism spectrum disorders. Journal of Psychopharmacology, 32(6), 641–653. <https://doi.org/10.1177/0269881118756245>

**275**. Fehlbaum, L. V., Raschle, N. M., Menks, W. M., Prätzlich, M., Flemming, E., et al. (2018). Altered neuronal responses during an affective stroop task in adolescents with conduct disorder. Frontiers in Psychology, 9. <https://doi.org/10.3389/fpsyg.2018.01961>

**276**. Schoorl, J., van Rijn, S., de Wied, M., van Goozen, S., and Swaab, H. (2016). Emotion regulation difficulties in boys with oppositional defiant disorder/conduct disorder and the relation with comorbid autism traits and attention deficit traits. PLOS ONE, 11(7):e0159323. <https://doi.org/10.1371/journal.pone.0159323>

**277**. Bauermeister, J. J., Shrout, P. E., Ramírez, R., Bravo, M., Alegría, M., Martínez-Taboas, A., Chávez, L., Rubio-Stipec, M., García, P., Ribera, J. C., and Canino, G. (2007). ADHD Correlates, Comorbidity, and Impairment in Community and Treated Samples of Children and Adolescents. Journal of Abnormal Child Psychology, 35(6), 883–898. <https://doi.org/10.1007/s10802-007-9141-4>

**278**. Leaberry, K. D., Rosen, P. J., Fogleman, N. D., Walerius, D. M., and Slaughter, K. E. (2020). Comorbid internalizing and externalizing disorders predict lability of negative emotions among children with ADHD. Journal of Attention Disorders, 24(14), 2001. <https://doi.org/10.1177/1087054717734647>

**279**. Ford, J. D., Connor, D. F., and Hawke, J. (2009). Complex trauma among psychiatrically impaired children: a cross-sectional, chart-review study. Journal of Clinical

Psychiatry, 70(8), 1155-1163. <https://doi.org/10.4088/JCP.08m04783>

**280**. Ford, J. D., Fraleigh, L. A., and Connor, D. F. (2010). Child abuse and aggression

among seriously emotionally disturbed children. Journal of Clinical Child and Adolescent Psychology, 39(1), 25-34. <https://doi.org/10.1080/15374410903401104>

**281**. Ford, J. D., Racusin, R., Daviss, W. B., Ellis, C. G., Thomas, J., Rogers, K., Reiser, J., Schiffman,J., and Sengupta, A. (1999). Trauma exposure among children with oppositional defiant disorder and attention deficit-hyperactivity disorder. Journal of Consulting and Clinical Psychology, 67(5), 786-789. <http://www.ncbi.nlm.nih.gov/pubmed/10535245>

**282**. Vasileva, M., Petermann, U., and Petermann, F. (2019). Traumatische Erfahrungen und Callous -unemotional Traits: Zusammenhang mit funktionalen und dysfunktionalen Emotionsregulationsstrategien. Zeitschrift für Psychiatrie, Psychologie und Psychotherapie, 67(2), 125–132. <https://doi.org/10.1024/1661-4747/a000380>

**283.** Hoskins, D., Marshall, B. D. L., Koinis-Mitchell, D., Galbraith, K., and Tolou-Shams, M. (2018). Latinx youth in first contact with the justice system: Trauma and associated behavioral health needs. Child Psychiatry & Human Development, 50(3), 459–472. <https://doi.org/10.1007/s10578-018-0855-z>

**284.** Cooley, J. L., Ritschel, L. A., Frazer, A. L., and Blossom, J. B. (2019). The influence of internalizing symptoms and emotion dysregulation on the association between witnessed community violence and aggression among urban adolescents. Child Psychiatry & Human Development, 50(6), 883–893. <https://doi.org/10.1007/s10578-019-00890-9>

**285**. Plattner, B., Karnik, N., Jo, B., Hall, R. E., Schallauer, A., Carrion, V., et al. (2007). State and trait emotions in delinquent adolescents. Child Psychiatry and Human Development, 38(2), 155–169. <https://doi.org/10.1007/s10578-007-0050-0>

**286**. Landis, T. D., Garcia, A. M., Hart, K. C., and Graziano, P. A. (2021). Differentiating Symptoms of ADHD in Preschoolers: The Role of Emotion Regulation and Executive Function. Journal of Attention Disorders, 25(9), 1260–1271. <https://doi.org/10.1177/1087054719896858>

**287**. Masi, G., Pisano, S., Milone, A., and Muratori, P. (2015). Child behavior checklist dysregulation profile in children with disruptive behavior disorders. A longitudinal study. Journal of Affective Disorders, 186, 249–253. <https://doi.org/10.1016/j.jad.2015.05.069>

**288**. Masi, G., Muratori, P., Manfredi, A., Pisano, S., and Milone, A. (2015). Child behaviour checklist emotional dysregulation profiles in youth with disruptive behaviour disorders: Clinical correlates and treatment implications. Psychiatry Research, 225(1-2), 191–196. <https://doi.org/10.1016/j.psychres.2014.11.019>

**289**. Tufan, E., Topal, Z., Demir, N., Taskiran, S., Savci, U., Akif Cansiz, M., and Semerci, B. (2016). Sociodemographic and clinical features of disruptive mood dysregulation disorder: A chart review. Journal of Child and Adolescent Psychopharmacology, 26(2), 94– 100. <https://doi.org/10.1089/cap.2015.0004>

**290**. Martin, S. E., Hunt, J. I., Mernick, L. R., DeMarco, M., Hunter, H. L., Coutinho, M. T, et al. (2016). Temper loss and persistent irritability in preschoolers: Implications for diagnosing disruptive mood dysregulation disorder in early childhood. Child Psychiatry & Human Development, 48(3):498–508. <https://doi.org/10.1007/s10578-016-0676-x>

**291**. Dickerson Mayes, S., Kokotovich, C., Mathiowetz, C., Baweja, R., Calhoun, S. L., and Waxmonsky, J. (2017). Disruptive mood dysregulation disorder symptoms by age in autism, ADHD, and general population samples. Journal of Mental Health Research in Intellectual Disabilities, 10(4):345–359. <https://doi.org/10.1080/19315864.2017.1338804>

**292**. Dougherty, L. R., Smith, V. C., Bufferd, S. J., Carlson, G. A, Stringaris, A., Leibenluft, E, et al. (2014). DSM-5 disruptive mood dysregulation disorder: correlates and predictors in young children. Psychological Medicine, 44(11):2339–2350. <https://doi.org/10.1017/s0033291713003115>

**293**. Zepf, F. D, Biskup, C. S., Holtmann, M., and Runions, K. (2016). Disruptive mood dysregulation disorder. In J.M. Rey, editor, IACAPAP e-Textbook of Child and Adolescent Mental Health. International

Association for Child and Adolescent Psychiatry and Allied Professions, Geneva, CH.

**294**. Adleman, N. E., Fromm, S. J., Razdan, V., Kayser, R., Dickstein, D. P, Brotman, M. A., et al. (2012). Cross-sectional and longitudinal abnormalities in brain structure in children with severe mood dysregulation or bipolar disorder. Journal of Child Psychology and Psychiatry, 53(11), 1149–1156. <https://doi.org/10.1111/j.1469-7610.2012.02568.x>

**295**. Deveney, C. M., Connolly, M. E., Jenkins, S. E., Kim, P., Fromm, S. J., and Pine, D. S. (2012). Neural recruitment during failed motor inhibition differentiates youths with bipolar disorder and severe mood dysregulation. Biological Psychology, 89(1), 148–155. <https://doi.org/10.1016/j.biopsycho.2011.10.003>

**3.1.8 Personality Disorder**

**66.** Fulford, D., Eisner, L. R., and Johnson, S. L. (2015). Differentiating risk for mania and borderline personality disorder: The nature of goal regulation and impulsivity. Psychiatry Research, 227(2-3), 347–352. <https://doi.org/10.1016/j.psychres.2015.02.001>

**81**. M. Linehan. (1993) Cognitive-behavioral treatment for borderline personality disorder. New York, NY: Guilford Press.

**88**. Dvir, Y., Ford, J. D., Hill, M., and Frazier, J. A. (2014). Childhood maltreatment, emotional dysregulation, and psychiatric comorbidities. Harvard Review of Psychiatry, 22(3), 149–161. https://doi.org/ 10.1097/hrp.0000000000000014

**185**. Weiss, N. H., Tull, M. T., Lavender, J., and Gratz, K. L. (2013). Role of emotion dysregulation in the relationship between childhood abuse and probable PTSD in a sample of substance abusers. Child Abuse & Neglect, 37(11), 944–954. <https://doi.org/10.1016/j.chiabu.2013.03.014>

**296**. El-Rasheed, A. H., ElAttar, K. S., Elrassas, H. H., Mahmoud, D. A. M., and Mohamed, S. Y. (2017). Mood regulation, alexithymia, and personality disorders in adolescent male addicts. Addictive Disorders & Their Treatment, 16(2), 49–58. <https://doi.org/10.1097/adt.0000000000000098>

**297**. Picardi, A., Toni, A., and Caroppo, E. (2005). Stability of alexithymia and its relationships with the ‘big five’ factors, temperament, character, and attachment style. Psychotherapy and Psychosomatics, 74(6):371–378. <https://doi.org/10.1159/000087785>

**298**. Narimani, M., Vahidi, Z., and Abolghasemi, A. (2013). Comparison alexithymia, impulsiovity and activation and inhibitiobn of the students with symptoms of obsessive - compulsive and paranoid personality disorder with normal individuals. Journal of Clinical Psychology, 5(2), 55–65. <https://doi.org/10.22075/jcp.2017.2127>

**299**. Reeves, M., James, L. M., Pizzarello, S. M., and Taylor, J. E. (2010). Support for Linehan's biosocial theory from a nonclinical sample. Journal of Personality Disorders, 24(3):312–326. <https://doi.org/10.1521/pedi.2010.24.3.312>

**300**. Sharp, C., Pane, H., Ha, C., Venta, A., Patel, A. B., Sturek, J., and Fonagy, P. (2011). Theory of mind and emotion regulation difficulties in adolescents with borderline traits. Journal of the American Academy of Child & Adolescent Psychiatry, 50(6):563–573.el. <https://doi.org/10.1016/j.jaac.2011.01.017>

**301.** Carpenter, R. W., and Trull, T. J. (2012). Components of emotion dysregulation in borderline personality disorder: A review. Current Psychiatry Reports, 15(1).

<https://doi.org/10.1007/s11920-012-0335-2>

**302.** Goodman, M., Hazlett, E. A., Avedon, J. B., Siever, D. R, Chu, K-W., and New, A. S, (2011). Anterior cingulate volume reduction in adolescents with borderline personality disorder and co-morbid major depression. Journal of Psychiatric Research, 45(6):803–807. <https://doi.org/10.1016/j.jpsychires.2010.11.011>

**303.** Xenaki, L-A., and Pehlivanidis, A. (2015). Clinical, neuropsychological and structural convergences and divergences between attention deficit/hyperactivity disorder and borderline personality disorder: A systematic review. Personality and Individual Differences, 86, 438–449. <https://doi.org/10.1016/j.paid.2015.06.049>

**304.** Krauch, M., Ueltzhöffer, K., Brunner, R., Kaess, M., Hensel, S., Herpertz, S. C., and Bertsch, K. (2018). Heightened salience of anger and aggression in female adolescents with borderline personality disorder—a script-based fMRI study. Frontiers in Behavioral Neuroscience, 12. <https://doi.org/10.3389/fnbeh.2018.00057>

**305**. Fonagy, P., and Luyten, P. (2009). A developmental, mentalization-based approach to the understanding and treatment of borderline personality disorder. Development and Psychopathology, 21(4):1355–1381. <https://doi.org/10.1017/s0954579409990198>

**306.** Selby, E. A., Anestis, M. D., Bender, T. W., and Joiner, T. E. (2009). An exploration of the emotional cascade model in borderline personality disorder. Journal of Abnormal Psychology, 118(2):375–387. <https://doi.org/10.1037/a0015711>

**307.** Fonagy, P., Target, M., Gergely, G., Allen, J. G., and Bateman, A. W. (2003). The developmental roots of borderline personality disorder in early attachment relationships: A theory and some evidence. Psychoanalytic Inquiry, 23(3), 412–459. <https://doi.org/10.1080/07351692309349042>

**308**. Kernberg, O. (1967). Borderline personality organization. Journal of the American Psychoanalytic Association, 15(3), 641–685. <https://doi.org/10.1177/000306516701500309>

**309**. Gratz, K. L., Kiel, E. J., Latzman, R. D., Elkin, T. D, Anne Moore, S., and Tull, M. T. (2014). Emotion: Empirical contribution: Maternal borderline personality pathology and infant emotion regulation: Examining the influence of maternal emotion-related difficulties and infant attachment. Journal of Personality Disorders, 28(1), 52–69. <https://doi.org/10.1521/pedi.2014.28.1.52>

**310**. Kim, S., Sharp, C., and Carbone, C. (2014). The protective role of attachment security for adolescent borderline personality disorder features via enhanced positive emotion regulation strategies. Personality Disorders: Theory, Research, and Treatment, 5(2), 125–136. <https://doi.org/10.1037/per0000038>

**311**. Crawford, T. N., Livesley, W. J., Jang, K. L., Shaver, P. R., Cohen, P., and Ganiban, J. (2007). Insecure attachment and personality disorder: a twin study of adults. European Journal of Personality, 21(2), 191–208. <https://doi.org/10.1002/per.602>

**312**. Sharp, C., Venta, A., Vanwoerden, S., Schramm, A., Ha, C., and Newlin, E. (2016). First empirical evaluation of the link between attachment, social cognition and borderline features in adolescents. Comprehensive Psychiatry, 64, 4–11. <https://doi.org/10.1016/j.comppsych.2015.07.008>

**313**. Kalpakci, A., Vanwoerden, S., Elhai, J. D., and Sharp, C. (2016). The independent contributions of emotion dysregulation and hypermentalization to the “double dissociation” of affective and cognitive empathy in female adolescent inpatients with BPD. Journal of Personality Disorders, 30(2), 242–260. <https://doi.org/10.1521/bumc.2016.80.3.255>

**314**. Lenzenweger, M. F., Clarkin, J. F., Fertuck, E. A., and Kernberg, O. F. (2004). Executive neurocognitive functioning and neurobehavioral systems indicators in borderline personality disorder: A preliminary study. Journal of Personality Disorders, 18(5), 421–438. <https://doi.org/10.1521/pedi.18.5.421.51323>

**315**. Jayaro, C., De La Vega, I., Bayon-Palomino, C., Díaz-Marsá, M., Montes, A., Tajima,

K., López-Ibor, J. J., and Carrasco, J. L. (2011). Depressive-type emotional response pattern in impulsive-aggressive patients with borderline personality disorder. Journal of Affective Disorders, 135(1-3):37–42. <https://doi.org/10.1016/j.jad.2011.06.040>

**316**. Schramm, A. T., Venta, A., and Sharp, C. (2013). The role of experiential avoidance in the association between borderline features and emotion regulation in adolescents. Personality Disorders: Theory, Research, and Treatment, 4(2):138–144. <https://doi.org/10.1037/a0031389>

**317**. Fossati, A., Gratz, K. L., Maffei, C., and Borroni, S. (2014). Impulsivity dimensions, emotion dysregulation, and borderline personality disorder featuresamong Italian nonclinical adolescents. Borderline Personality Disorder and Emotion Dysregulation, 1(5), 1–11. <https://doi.org/10.1186/2051-6673-1-5>

**318**. Yen, S., Gagnon, K., and Spirito, A. (2012). Borderline personality disorder in suicidal adolescents. Personality and Mental Health, 7(2):89–101. <https://doi.org/10.1002/pmh.1216>

**319**. Mancke, F., Herpertz, S. C., and Bertsch, K. (2015). Aggression in borderline personality disorder: A multidimensional model. Personality Disorders: Theory, Research, and Treatment, 6(3), 278–291. <https://doi.org/10.1037/per0000098>

**320**. Mancke, I., Herpertz, S. C., Kleindienst, N., and Bertsch, K. (2017). Emotion dysregulation and trait anger sequentially mediate the association between borderline personality disorder and aggression. Journal of Personality Disorders, 31(2), 256–272. <https://doi.org/10.1521/pedi_2016_30_247>

**321**. Banny, A. M., Tseng, W-L., Murray-Close, D., Pitula, C. E., and Crick, N. R. (2014). Borderline personality features as a predictor of forms and functions of aggression during middle child-hood: Examining the roles of gender and physiological reactivity. Development and Psychopathology, 26(3), 789–804. <https://doi.org/10.1017/s095457941400039x>

**322**. Gaher, R. M., Hofman, N. L., Simons, J. S., and Hunsaker, R. (2013). Emotion regulation deficits as mediators between trauma exposure and borderline symptoms. Cognitive Therapy and Research, 37(3), 466–475. <https://doi.org/10.1007/s10608-012-9515-y>

**323**. van Dijke, A., Ford, J. D., van der Hart, L., van Son, J. D., and Maarten, F. (2013). Association of childhood-trauma-by-primary caregiver and affect dysregulation with borderline personality disorder symptoms in adulthood. Psychological Trauma Theory Research Practice and Policy, 5(3), 217–224. <https://doi.org/10.1037/a0027256>

**324**. Ford, J. D., and Courtois, C. A. (2021). Complex PTSD and Borderline Personality Disorder. Borderline Personal Disorder and Emotion Dysregulation, 8(1), 16. <https://doi.org/10.1186/s40479-021-00155-9>

**325**. Yen, S., Frazier, E., Hower, H., Weinstock, L. M., Topor, D. R., Hunt, J., Goldstein, T. R., et al. (2015). Borderline personality disorder in transition age youth with bipolar disorder. Acta Psychiatrica Scandinavica, 132(4):270–280. <https://doi.org/10.1111/acps.12415>

**326.** Winsper, C., and Tang, N. K. Y. (2014). Linkages between insomnia and suicidality: Prospective associations, high-risk subgroups and possible psychological mechanisms. International Review of Psychiatry, 26(2), 189-204. <https://doi.org/10.3109/09540261.2014.881330>

**327**. Marco, J. H., Pérez, S., García-Alandete, J, and Moliner, R. (2015). Meaning in life in people with Borderline Personality Disorder. Clinical Psychology & Psychotherapy, 24(1), 162–170. <https://doi.org/10.1002/cpp.1991>

**328**. Stanton, K., Rozek, D. C., Stasik-O'Brien, S. M., Ellickson-Larew, S., and Watson, D. A. (2016). Transdiagnostic approach to examining the incremental predictive power of emotion regulation and basic personality dimensions. Journal of Abnormal Psychology, 125(7), 960–975. <https://doi.org/10.1037/abn0000208>

**329**. Cheshure, A., Zeigler-Hill, V., Sauls, D., Vrabel, J. K., and Lehtman, M. J. (2020). Narcissism and emotion dysregulation: Narcissistic admiration and narcissistic rivalry have divergent associations with emotion regulation difficulties. Personality and Individual Differences, 154:109679. <https://doi.org/10.1016/j.paid.2019.109679>

**330**. Back, M. D., Küfner, A. C. P., Dufner, M., Gerlach, T. M., Rauthmann, J. F, and Denissen, J. J. A. (2013). Narcissistic admiration and rivalry: Disentangling the bright and dark sides of narcissism. Journal of Personality and Social Psychology, 105(6), 1013–1037. <https://doi.org/10.1037/a0034431>

**331**. Yang, Y., Narr K. L., Baker, L. A., Joshi, S. H., Jahanshad, N., Raine, A., and Thompson, P. M. (2015). Frontal and striatal alterations associated with psychopathic traits in adolescents. Psychiatry

**3.1.9 Substance Use Disorder**

**114**. McLaughlin, K. A., Hatzenbuehler, M. L., and Hilt, L. M. (2009). Emotion dysregulation as a mechanism linking peer victimization to internalizing symptoms in adolescents. Journal of Consulting and Clinical Psychology, 77(5), 894–904. <https://doi.org/10.1037/a0015760>

**138**. Ghorbani, F., Khosravani, V., Mohammadzadeh, A., and Shadnia, S. (2019). The role of emotion dysregulation in the relation of childhood trauma to heroin craving in individuals with heroin dependence. Drug and Alcohol Dependence, 195, 132–139. <https://doi.org/10.1016/j.drugalcdep.2018.12.008>

**185**. Weiss, N. H., Tull, M. T., Lavender, J., and Gratz, K. L. (2013). Role of emotion dysregulation in the relationship between childhood abuse and probable PTSD in a sample of substance abusers. Child Abuse & Neglect, 37(11), 944–954. <https://doi.org/10.1016/j.chiabu.2013.03.014>

**332**. Wills. T. A., Simons, J. S, Sussman, S., and Knight, R. (2016). Emotional self-control and dysregulation: A dual-process analysis of pathways to externalizing/internalizing symptomatology and positive well-being in younger adolescents. Drug and Alcohol Dependence, 163:S37–S45. <https://doi.org/10.1016/j.drugalcdep.2015.08.039>

**333**. Englund, M. M, Egeland, B., Oliva, E. M, and Collins, W. A. (2008). Childhood and adolescent predictors of heavy drinking and alcohol use disorders in early adulthood: a

longitudinal developmental analysis. Addiction, 103(s1), 23–35. [https://doi.org/10.1111/j.1360- 0443.2008.02174.x](https://doi.org/10.1111/j.1360-%200443.2008.02174.x)

**334**. Boulos, P. K., Dalwani, M. S., Tanabe, J., Mikulich-Gilbertson, S. K, Banich, M. T., Crowley, T. J., and Sakai, J. T. (2016). Brain cortical thickness differences in adolescent females with substance use disorders. PLOS ONE, 11(4):e0152983. <https://doi.org/10.1371/journal.pone.0152983>

**335**. Tan, H., Ahmad, T., Loureiro, M., Zunder, J., and Laviolette, S. R. (2014). The role of cannabinoid transmission in emotional memory formation: Implications for addiction and schizophrenia. Frontiers in Psychiatry, 5. <https://doi.org/10.3389/fpsyt.2014.00073>

**336**. Ilbegi, S., Groenman, A. P., Schellekens, A., Hartman, C. A., Hoekstra, P. J., and Franke, B. (2018). Substance use and nicotine dependence in persistent, remittent, and late- onset ADHD: a 10-year longitudinal study from childhood to young adulthood. Journal of Neurodevelopmental Disorders, 10(1). <https://doi.org/10.1186/s11689-018-9260-y>

**337**. Hasler, B. P., Soehner, A. M., and Clark, D. B. (2015). Sleep and circadian contributions to adolescent alcohol use disorder. Alcohol, 49(4), 377–387. <https://doi.org/10.1016/j.alcohol.2014.06.010>

**338**. Cheetham, A., Allen, N. B., Yücel, M., and Lubman, D. I. (2020). The role of affective dysregulation in drug addiction. Clinical Psychology Review, 30(6), 621–634.

<https://doi.org/10.1016/j.cpr.2010.04.005>

**339**. Simons, J. S., and Carey, K. B. (2002). Risk and vulnerability for marijuana use problems: The role of affect dysregulation. Psychology of Addictive Behaviors, 16(1), 72– 75. <https://doi.org/10.1037/0893-164x.16.1.72>

**340**. Brown, L. K., Houck, C., Lescano, C., Donenberg, G., Tolou-Shams, M., and Mello, J. (2012). Affect regulation and HIV risk among youth in therapeutic schools. AIDS and Behavior, 16(8), 2272–2278. <https://doi.org/10.1007/s10461-012-0220-3>

**341.** Brumback, T., Worley, M., Nguyen-Louie, T. T., Squeglia, L. M., Jacobus, J., and Tapert, S. F. (2016). Neural predictors of alcohol use and psychopathology symptoms in adolescents. Development and Psychopathology, 28(4pt1):1209–1216. <https://doi.org/10.1017/s0954579416000766>

**342**. Weinstein, S. M., Mermelstein, R., Shiffman, S., and Flay, B. (2008). Mood variability and cigarette smoking escalation among adolescents. Psychology of Addictive Behaviors, 22(4):504–513. <https://doi.org/10.1037/0893-164x.22.4.504>

**343**. Khantzian, E. J. (1985). The self-medication hypothesis of addictive disorders: Focus on heroin and cocaine dependence. The American Journal of Psychiatry, 142, 1259–1264. <https://doi.org/10.1176/ajp.142.11.1259>

**344**. Brook, J. S., Zhang, C., Leukefeld, C. G, and Brook, D. W. (2016). Marijuana use from adolescence to adulthood: developmental trajectories and their outcomes. Social Psychiatry and Psychiatric Epidemiology, 51(10):1405–1415. <https://doi.org/10.1007/s00127-016-1229-0>

**345**. Bonn-Miller, M. O., Vujanovic, A. A., and Zvolensky, M. J. (2008). Emotional dysregulation: Association with coping-oriented marijuana use motives among current marijuana users. Substance Use & Misuse, 43(11), 1653–1665. <https://doi.org/10.1080/10826080802241292>

**346**. Dorard, G., Berthoz, S., Phan, O., Corcos, M., and Bungener, C. (2008). Affect dysregulation in cannabis abusers. European Child & Adolescent Psychiatry, 17(5):274– 282. <https://doi.org/10.1007/s00787-007-0663-7>

**347**. Clark, D. B., Chung, T., Thatcher, D. L., Pajtek, S., and Long, E. C. (2011). Psychological dysregulation, white matter disorganization and substance use disorders in adolescence. Addiction, 107(1):206–214. <https://doi.org/10.1111/j.1360-0443.2011.03566.x>

**348**. Bava, S, Jacobus, J., Thayer, R. E., and Tapert, S. F. (2012). Longitudinal changes in white matter integrity among adolescent substance users. Alcoholism: Clinical and Experimental Research, 37:E181–E189. <https://doi.org/10.1111/j.1530-0277.2012.01920.x>

**349**. Coban, F. R., Kunst, A. E., Van Stralen, M. M., Richter, M., Rathmann, K., Perelman, J., et al. (2018). Nicotine dependence among adolescents in the European Union: How many and who are affected? Journal of Public Health, 41(3), 447–455. <https://doi.org/10.1093/pubmed/fdy136>

**350**. Novak, S. P., and Clayton, R. R. (2001). The influence of school environment and self- regulation on transitions between stages of cigarette smoking: A multilevel analysis. Health Psychology, 20(3), 196–207. <https://doi.org/10.1037/0278-6133.20.3.196>

**351**. Wilens, T. E., Martelon, M. K., Anderson, J. P., Shelley-Abrahamson, R., and Biederman, J. (2013). Difficulties in emotional regulation and substance use disorders: A controlled family study of bipolar adolescents. Drug and Alcohol Dependence, 132(1-2), 114–121. <https://doi.org/10.1016/j.drugalcdep.2013.01.015>

**352**. Jessica L. Combs, J. L., Nichea S. Spillane, N. S, Leann Caudill, L., Brittany Stark, B., and Gregory T. Smith, G. T. (2012). The acquired preparedness risk model applied to smoking in 5th grade children. Addictive Behaviors, 37(3), 331–334. <https://doi.org/10.1016/j.addbeh.2011.11.005>

**353**. Dir, A. L., Banks, D. E., Zapolski, T. C. B., McIntyre, E., and Hulvershorn, L. A. (2016). Negative urgency and emotion regulation predict positive smoking expectancies in non-

smoking youth. Addictive Behaviors, 58, 47–52. <https://doi.org/10.1016/j.addbeh.2016.02.014>

**354**. Colder, C. R., and Stice, E. (1998). A longitudinal study of the interactive effects of impulsivity and anger on adolescent problem behavior. Journal of Youth and Adolescence, 27(3), 255–274. <https://doi.org/10.1023/A:1022889202419>

**355.** Mischel, E. R., Leen-Feldner, E. W., Knapp, A. A., Bilsky, S. A, Hama, L., and Lewis, S. (2014). Indirect effects of smoking motives on adolescent anger dysregulation and smoking. Addictive Behaviors, 39, 1831–1838. <https://doi.org/10.1016/j.addbeh.2014.07.029>

**356**. Kassel, J. D., Stroud, L. R., and Paronis, C. A. (2003). Smoking, stress, and negative affect: Correlation, causation, and context across stages of smoking. Psychological Bulletin, 129(2), 270–304. https://doi.org/10.1037/0033-2909.129.2.270

**357**. Mermelstein, R., Hedeker, D., Flay, B. R., and Saul Shiffman, S. (2007). Real-time data capture and adolescent cigarette smoking. <https://www.researchgate.net/publication/265530314> [Accessed July 26, 2020]

**358**. Whalen, C. K., Jamner, L. D., Henker, B., and Delfino, R. J. (2001). Smoking and moods in adolescents with depressive and aggressive dispositions: Evidence from surveys and electronic diaries.

Health Psychology, 20(2), 99–111. <https://doi.org/10.1037/0278-6133.20.2.99>

**359**. Weinstein, S. M., and Mermelstein, R. J. (2013). Dynamic associations of negative mood and smoking across the development of smoking in adolescence. Journal of Clinical Child & Adolescent Psychology, 42(5), 629–642. <https://doi.org/10.1080/15374416.2013.794698>

**360**. Treloar Padovano, H., Merrill, J. E., Colby, S. M., Kahler, C. W, and Gwaltney, C. J. (2019). Affective and situational precipitants of smoking lapses among adolescents. Nicotine & Tobacco Research, 22(4), 492–497. <https://doi.org/10.1093/ntr/ntz002>

**361**. Kovacs, M., Sherrill, J., George, C. J, Pollock, M., Tumuluru, R. V., and Ho, V. (2006). Contextual emotion-regulation therapy for childhood depression: Description and pilot testing of a new intervention. Journal of the American Academy of Child & Adolescent Psychiatry, 45(8), 892–903. <https://doi.org/10.1097/01.chi.0000222878.74162.5a>

**362**. Banducci, A. N., Hoffman, E. M., Lejuez, C. W., and Koenen, K. C. (2014). The impact of childhood abuse on inpatient substance users: Specific links with risky sex, aggression, and emotion dysregulation. Child Abuse & Neglect, 38(5), 928–938. <https://doi.org/10.1016/j.chiabu.2013.12.007>

**363.** Barahmand, U., Khazaee, A., and Sadeghi Hashjin, G. (2016). Emotion dysregulation mediates between childhood emotional abuse and motives for substance use. Archives of Psychiatric Nursing, 30(6), 653–659. <https://doi.org/10.1016/j.apnu.2016.02.007>

**364**. Li. D., Li, D., Wu, N., and Wang, Z. (2019). Intergenerational transmission of emotion regulation through parents' reactions to children's negative emotions: Tests of unique, actor, partner, and mediating effects. Children and Youth Services Review, 101, 113–122. <https://doi.org/10.1016/j.childyouth.2019.03.038>

**365**. Li, Z., Coles, C. D., Lynch, M. E., Hamann, S., Peltier, S., LaConte, S., and Hu, X. (2009). Prenatal cocaine exposure alters emotional arousal regulation and its effects on working memory. Neurotoxicology and Teratology, 31(6), 342–348. <https://doi.org/10.1016/j.childyouth.2019.03.038>

**3.1.10 Developmental Disorder including ASD**

**31**. American Psychiatric Association. (2013). Diagnostic and Statistical Manual of Mental Disorders (DSM-5®), Fifth Edition. Washington, DC: American Psychiatric Association.

**99**. Samson, A. C., Huber, O., and Gross, J. J. (2012). Emotion regulation in Asperger's syndrome and high-functioning autism. Emotion, 12(4), 659–665. <https://doi.org/10.1037/a0027975>

**274.** Sagar-Ouriaghli, I., Lievesley, K., and Santosh, P. J. (2018). Propranolol for treating emotional, behavioural, autonomic dysregulation in children and adolescents with autism spectrum disorders. Journal of Psychopharmacology, 32(6), 641–653. <https://doi.org/10.1177/0269881118756245>

**291**. Dickerson Mayes, S., Kokotovich, C., Mathiowetz, C., Baweja, R., Calhoun, S. L., and Waxmonsky, J. (2017). Disruptive mood dysregulation disorder symptoms by age in autism, ADHD, and general population samples. Journal of Mental Health Research in Intellectual Disabilities, 10(4):345–359. <https://doi.org/10.1080/19315864.2017.1338804>

**366**. Baker, J. K., Fenning, R. M., and Moffitt, J. (2019). A cross-sectional examination of the internalization of emotion co-regulatory support in children with ASD. Journal of

Autism and Developmental Disorders, 49(10), 4332–4338. [https://doi.org/10.1007/s10803-019- 04091-0](https://doi.org/10.1007/s10803-019-%2004091-0)

**367.** Brereton, A. V., Tonge, B. J, and Einfeld, S. L. (2006). Psychopathology in children and adolescents with autism compared to young people with intellectual disability. Journal of Autism and Developmental Disorders, 36(7), 863–870. <https://doi.org/10.1007/s10803-006-0125-y>

**368**. Lecavalier, L., Leone, S., and Wiltz, J. (2006). The impact of behaviour problems on caregiver stress in young people with autism spectrum disorders. Journal of Intellectual Disability Research, 50(3):172–183. <https://doi.org/10.1111/j.1365-2788.2005.00732.x>

**369**. Mazefsky, C. A., and White, S. W. (2014). Emotion regulation. Child and Adolescent Psychiatric

Clinics of North America, 23(1), 15–24. <https://doi.org/10.1016/j.chc.2013.07.002>

**370**. Samson, A. C., Hardan, A. Y., Podell, R. W., Phillips, J. M, and Gross, J. J. (2014). Emotion regulation in children and adolescents with autism spectrum disorder. Autism Research, 8(1), 9–18. <https://doi.org/10.1002/aur.1387>

**371**. Berkovits, L., Eisenhower, A., and Blacher, J. (2016). Emotion regulation in young children with autism spectrum disorders. Journal of Autism and Developmental Disorders, 47(1), 68–79. <https://doi.org/10.1007/s10803-016-2922-2>

**372**. Jahromi, L. B., Meek, S. E., and Ober-Reynolds, S. (2012). Emotion regulation in the context of frustration in children with high functioning autism and their typical peers. Journal of Child Psychology and Psychiatry, 53(12), 1250–1258. <https://doi.org/10.1111/j.1469-7610.2012.02560.x>

**373**. Jahromi, L. B., Bryce, C. I., and Swanson, J. (2013). The importance of self-regulation for the school and peer engagement of children with high-functioning autism. Research in Autism Spectrum Disorders, 7(2), 235–246. <https://doi.org/10.1016/j.rasd.2012.08.012>

**374**. Joshi, G., Wozniak, J., Fitzgerald, M., Faraone, S., Fried, R., Galdo, M., et al. (2018). High risk for severe emotional dysregulation in psychiatrically referred youth with autism spectrum disorder: A controlled study. Journal of Autism and Developmental Disorders, 48(9), 3101–3115. <https://doi.org/10.1007/s10803-018-3542-9>

**375**. Pitskel, N. B., Bolling, D. Z., Kaiser, M. D, Pelphrey, K. A., and Crowley, M. J. (2014). Neural systems for cognitive reappraisal in children and adolescents with autism spectrum disorder. Developmental Cognitive Neuroscience, 10, 117–128. <https://doi.org/10.1016/j.dcn.2014.08.007>

**376**. Stark, K. H., Barnes, J. C., Young, N. D., and Gabriels, R. L. (2015). Brief report: Understanding crisis behaviors in hospitalized psychiatric patients with autism spectrum disorder—Iceberg Assessment Interview. Journal of Autism and Developmental Disorders, 45(11), 3468–3474. <https://doi.org/10.1007/s10803-015-2552-0>

**377**. López-Pérez, B., Ambrona, T., and Gummerum, M. (2018). Emotional preferences and goals and emotion dysregulation in children with Asperger's syndrome and typically developing children. British Journal of Clinical Psychology, 57(3), 274–290. <https://doi.org/10.1111/bjc.12173>

**378**. Jane E. Gillham, Alice S. Carter, Fred R. Volkmar, and Sara S. S p a r r o w . Toward a developmental operational definition of autism. Journal of Autism and Developmental Disorders, 30(4), 269–278. <https://doi.org/10.1023/A:1005571115268>

**379**. Samson, A. C.,Wells, W. M., Phillips, J. M., Hardan, A. Y., and Gross, J. J. (2014). Emotion regulation in autism spectrum disorder: evidence from parent interviews and children’s daily diaries. Journal of Child Psychology and Psychiatry, 56(8), 903–913. <https://doi.org/10.1111/jcpp.12370>

**380**. Gadow, K. D., Pinsonneault, J. K., Perlman, G., and Sadee, W. (2014). Association of dopamine gene variants, emotion dysregulation and ADHD in autism spectrum disorder. Research in Developmental Disabilities, 35(7):1658–1665. <https://doi.org/10.1016/j.ridd.2014.04.007>

**381**. Rieffe, C., Camodeca, M., Pouw, L. B. C., Lange, A. M. C., and Stockmann, L. (2012). Don't anger me! Bullying, victimization, and emotion dysregulation in young adolescents with ASD. European Journal of Developmental Psychology, 9(3), 351–370. <https://doi.org/10.1080/17405629.2012.680302>

**382**. Rieffe, C., Oosterveld, P., Meerum Terwogt, M., Mootz, S., van Leeuwen, E., and Stockmann, L. (2011). Emotion regulation and internalizing symptoms in children with autism spectrum disorders. Autism, 15(6), 655–670. <https://doi.org/10.1177/1362361310366571>

**383**. Swain, D., Scarpa, A., White, S., and Laugeson, E. (2015). Emotion dysregulation and anxiety in adults with ASD: Does social motivation play a role? Journal of Autism and Developmental Disorders, 45(12), 3971–3977. <https://doi.org/10.1007/s10803-015-2567-6>

**384**. Vasa, R. A., Kreiser, N. L, Keefer, A., Singh, V., and Mostofsky, S. H. (2018). Relationships between autism spectrum disorder and intolerance of uncertainty. Autism Research, 11(4), 636–644. <https://doi.org/10.1002/aur.1916>

**385**. Richdale, A. L., and Kimberly A, K. (2009). Schreck. Sleep problems in autism spectrum disorders: Prevalence, nature, & possible biopsychosocial aetiologies. Sleep Medicine Reviews, 13(6), 403–411. <https://doi.org/10.1016/j.smrv.2009.02.003>

**386.** Taylor, M. A., Schreck, K. A., and Mulick, J. A. (2012). Sleep disruption as a correlate to cognitive and adaptive behavior problems in autism spectrum disorders. Research in Developmental Disabilities, 33(5), 1408–1417. <https://doi.org/10.1016/j.ridd.2012.03.013>

**387**. Zaidman-Zait, A., Zwaigenbaum, L., Duku, E., Bennett, T., Szatmari, P., Mirenda, P., et al. (2020). Factor analysis of the children’s sleep habits questionnaire among preschool children with autism spectrum disorder. Research in Developmental Disabilities, 97, 103548. <https://doi.org/10.1016/j.ridd.2019.103548>

**388**. Fenning, R. M., Baker, J. K., and Moffitt, J. (2018). Intrinsic and extrinsic predictors of emotion regulation in children with autism spectrum disorder. Journal of Autism and Developmental Disorders, 48(11), 3858–3870. <https://doi.org/10.1007/s10803-018-3647-1>

**389**. Simonoff, E., Jones, C. R. G., Pickles, A., Happé, F., Baird, G., and Charman, T. (2012). Severe mood problems in adolescents with autism spectrum disorder. Journal of Child Psychology and Psychiatry, 53(11), 1157–1166. [https://doi.org/10.1111/j.1469- 7610.2012.02600.x](https://doi.org/10.1111/j.1469-%207610.2012.02600.x)

**390**. Uljarevic ́, M., Hedley, D., Nevill, R., Evans, D. W., Ying Cai, R., Butter, E., and Mulick, J. A. (2018). Brief report: Poor self-regulation as a predictor of individual differences in adaptive functioning in young children with autism spectrum disorder. Autism Research, 11(8), 1157–1165. <https://doi.org/10.1002/aur.1953>

**391**. Mazefsky, C. A., Day, T. N., Siegel, M., White, S. W., Yu, L., and Pilkonis, P. A. (2016). Development of the emotion dysregulation inventory: A PROMIS®ing method for creating sensitive and unbiased questionnaires for autism spectrum disorder. Journal of Autism and Developmental Disorders, 48(11), 3736–3746. <https://doi.org/10.1007/s10803-016-2907-1>

**392**. Mazefsky, C. A., Yu, L., White, S. W., Siegel, M., and Pilkonis, P. A. (2018). The emotion dysregulation inventory: Psychometric properties and item response theory

calibration in an autism spectrum disorder sample. Autism Research, 11(6), 928–941. <https://doi.org/10.1002/aur.1947>

**3.1.11 Psychosis and Schizophrenia**

**98**. Conley, R. R., Ascher-Svanum, H., Zhu, B., Faries, D., and Kinon, B. J. (2007). The burden of depressive symptoms in the long-term treatment of patients with schizophrenia. Schizophrenia Research, 90(1-3), 186–197. <https://doi.org/10.1016/j.schres.2006.09.027>

**393**. Jerrell, J. M., McIntyre, R. S., and Deroche, C. B. (2017). Diagnostic clusters associated with an early onset schizophrenia diagnosis among children and adolescents. Human Psychopharmacology: Clinical and Experimental, 32(2), e2589. <https://doi.org/10.1002/hup.2589>

**394**. Kelleher, I., Connor, D., Clarke, M. C, Devlin, N., Harley, M., and Cannon, M. (2012). Prevalence of psychotic symptoms in childhood and adolescence: a systematic review and meta-analysis of population-based studies. Psychological Medicine, 42(9), 1857–1863. <https://doi.org/10.1017/s0033291711002960>

**395**. Okkels, N., Vernal, D. L., Jensen, S. O. W., McGrath, J. J., and Nielsen, R. E. (2012). Changes in the diagnosed incidence of early onset schizophrenia over four decades. Acta Psychiatrica Scandinavica, 127(1), 62–68. <https://doi.org/10.1111/j.1600-0447.2012.01913.x>

**396**. Millier, A., Schmidt, U., Angermeyer, M. C., Chauhan, D., Murthy, V., Toumi, M., and Cadi-Soussi, N. (2014). Humanistic burden in schizophrenia: A literature review. Journal of Psychiatric Research, 54, 85 93. <https://doi.org/10.1016/j.jpsychires.2014.03.021>

**397**. Tandon, R., Gaebel, W., Barch, D. M., Bustillo, J., Gur, R. E., Heckers, S., et al. (2013). Definition and description of schizophrenia in the DSM-5. Schizophrenia Research, 150(1), 3–10. <https://doi.org/10.1016/j.schres.2013.05.028>

398. DeLoore, E., Gunther, N., Drukker, M., Feron, F., Sabbe, B., Deboutte, D., et al. (2011). Persistence and outcome of auditory hallucinations in adolescence: A longitudinal general population study of 1800 individuals. Schizophrenia Research, 127(1-3), 252–256. <https://doi.org/10.1016/j.schres.2011.01.015>

**399**. Morrison, A. P. (2001). The interpretation of intrusions in psychosis: An integrative cognitive approach to hallucinations and delusions. Behavioural and Cognitive Psychotherapy, 29(3), 257–276. <https://doi.org/10.1017/s1352465801003010>

**400**. Scott, J., Martin, G., Bor, W., Sawyer, M., Clark, J., and McGrath, J. (2009). The prevalence and correlates of hallucinations in Australian adolescents: Results from a national survey. Schizophrenia Research, 107(2-3), 179–185. <https://doi.org/10.1016/j.schres.2008.11.002>

**401**. Fonseca-Pedrero, E., Paino, M., Lemos-Giráldez, S., and Muñiz, J. (2011). Schizotypal traits and depressive symptoms in nonclinical adolescents. Comprehensive Psychiatry, 52(3), 293–300. <https://doi.org/10.1016/j.comppsych.2010.07.001>

**402**. Fonseca-Pedrero, E., Lemos-Giráldez, S., Paino, M., and Muñiz, J. (2011). Schizotypy, emotional–behavioural problems and personality disorder traits in a non-clinical adolescent population. Psychiatry Research, 190(2-3), 316–321. <https://doi.org/10.1016/j.psychres.2011.07.007>

**403**. Gooding, D. C., Tallent, K. A., and Matts, C. W. (2005). Clinical status of at-risk individuals 5 years later: Further validation of the psychometric high-risk strategy. Journal of Abnormal Psychology, 114(1), 170–175. <https://doi.org/10.1037/0021-843x.114.1.170>

**404**. Kwapil, T. R., Barrantes-Vidal, N., and Silvia, P. J. (2007). The dimensional structure of the Wisconsin schizotypy scales: Factor identification and construct validity. Schizophrenia Bulletin, 34(3), 444–457. <https://doi.org/10.1093/schbul/sbm098>

**405**. Raine, A. (2006). Schizotypal personality: Neurodevelopmental and psychosocial trajectories. Annual Review of Clinical Psychology, 2(1), 291–326. <https://doi.org/10.1146/annurev.clinpsy.2.022305.095318>

**3.1.12 Gaming Disorder**

**406**. Rideout, V. J., Foehr, U. G., and Roberts, D. F. (2010). Generation m2. Media in the lives of 8- to 18-year-olds. A Kaiser Family Foundation study. <https://files.eric.ed.gov/fulltext/ED527859.pdf>. [Accessed September 7, 2020]

**407**. Mihara, S., and Higuchi, S. (2017). Cross-sectional and longitudinal epidemiological studies of Internet Gaming Disorder: A systematic review of the literature. Psychiatry and Clinical Neurosciences, 71(7), 425–444. <https://doi.org/10.1111/pcn.12532>

**408**. Amendola, S., Spensieri, V., Guidetti, V., and Cerutti, R. (2018). The relationship between difficulties in emotion regulation and dysfunctional technology use among adolescents. Journal of Psychopathology, 25, 10–17. <https://doi.org/10.1111/sjop.12664>

**409**. Mo. P. K. H., Chan, V. W. Y., Chan, S. W, and Lau, J. T. F. (2018). The role of social support on emotion dysregulation and internet addiction among Chinese adolescents: A structural equation model. Addictive Behaviors, 82, 86–93. <https://doi.org/10.1016/j.addbeh.2018.01.027>

**410.** Casale, S., Caplan, S. E., and Fioravanti, G. (2016). Positive metacognitions about internet use: The mediating role in the relationship between emotional dysregulation and problematic use. Addictive Behaviors, 59, 84–88. <https://doi.org/10.1016/j.addbeh.2016.03.014>

**411**. Donald, J. N., Ciarrochi, J., and Sahdra, B. K. (2020). The consequences of compulsion: A 4-year longitudinal study of compulsive internet use and emotion regulation difficulties. Emotion. https://doi.org/10.1037/emo0000769

International Journal of Mental Health and Addiction

<https://doi.org/10.1007/s11469-011-9318-5>

**412** Kuss, D. J., and Griffiths, M. D. (2012). Internet gaming addiction: A systematic review

of empirical research. International Journal of Mental Health and Addiction, 10, 278-296.

<https://doi.org/10.1007/s11469-011-9318-5>

**413**. Paulus, F. W., Ohmann, S., von Gontard, A., and Popow, C. (2018). Internet gaming disorder in children and adolescents: a systematic review. Developmental Medicine & Child Neurology, 60(7), 645–659. <https://doi.org/10.1111/dmcn.13754>

**414**. Paulus, F. W., Hübler, K., Mink, F., and Möhler, E. (2021). Emotional dysregulation in

preschool age predicts later media use and Gaming Disorder symptoms in childhood. Frontiers in Psychiatry,12, 626387. <https://doi.org/10.3389/fpsyt.2021.626387>

**415**. Gaetan, S., Bréjard, V., and Bonnet, A. (2016). Video games in adolescence and emotional functioning: Emotion regulation, emotion intensity, emotion expression, and alexithymia. Computers in Human Behavior, 61, 344–349. <https://doi.org/10.1016/j.chb.2016.03.027>

**416**. Hollett, K. B., and Harris, N. (2019). Dimensions of emotion dysregulation associated with problem video gaming. Addiction Research & Theory, 28(1), 38–45. <https://doi.org/10.1080/16066359.2019.1579801>

**417**. Wichstrøm, L., Stenseng, F., Belsky, J., von Soest, T., and Wold Hygen, B. (2018). Symptoms of Internet Gaming Disorder in youth: Predictors and comorbidity. Journal of Abnormal Child Psychology, 47(1), 71–83. <https://doi.org/10.1007/s10802-018-0422-x>
